# Supplementary material for: Functional contribution of mesencephalic locomotor region nuclei to locomotor recovery after spinal cord injury
Source: Cell Rep Med. 2023 Feb 21;4(2):100946. doi: 10.1016/j.xcrm.2023.100946 (PMC9975330; doi:10.1016/j.xcrm.2023.100946)
Supplement: Document S1. Figures S1–S13 [file mmc1.pdf]

Cell Reports Medicine, Volume *4*

# Supplemental information

# Functional contribution of mesencephalic locomotor region nuclei to locomotor recovery after spinal cord injury

**Marie Roussel, David Lafrance-Zoubga, Nicolas Josset, Maxime Lemieux, and Frederic Bretzner**

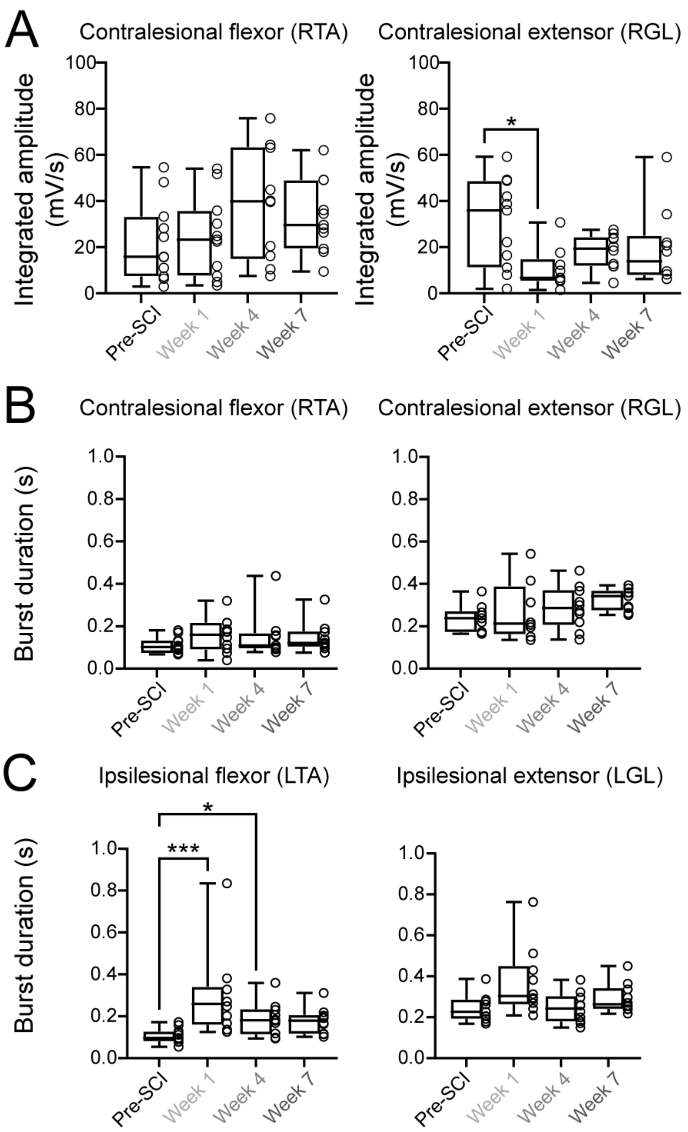


**Fig. S1: Background EMG activity in the contralesional flexor and extensor muscles after SCI. Related to Figure 1.**

(A) Integrated amplitude of the contralesional right flexor and extensor (n = 11 mice, Kruskal-Wallis test [$p = 0.049$] with Dunn's multiple comparisons test, *$p < 0.05$).

(B-C) Burst duration of the contra- (B) and ipsilesional (C) flexor and extensor muscles (n = 11 mice, LTA Kruskal-Wallis test [$p = 0.0007$] with Dunn's multiple comparisons test, *$p < 0.05$, ***$p < 0.001$; LGL Kruskal-Wallis test [$p = 0.0428$] with Dunn's multiple comparisons test).

Abbreviations: LTA /RTA, left/right tibialis anterior, LGL/RGL, left/right gastrocnemius lateralis.

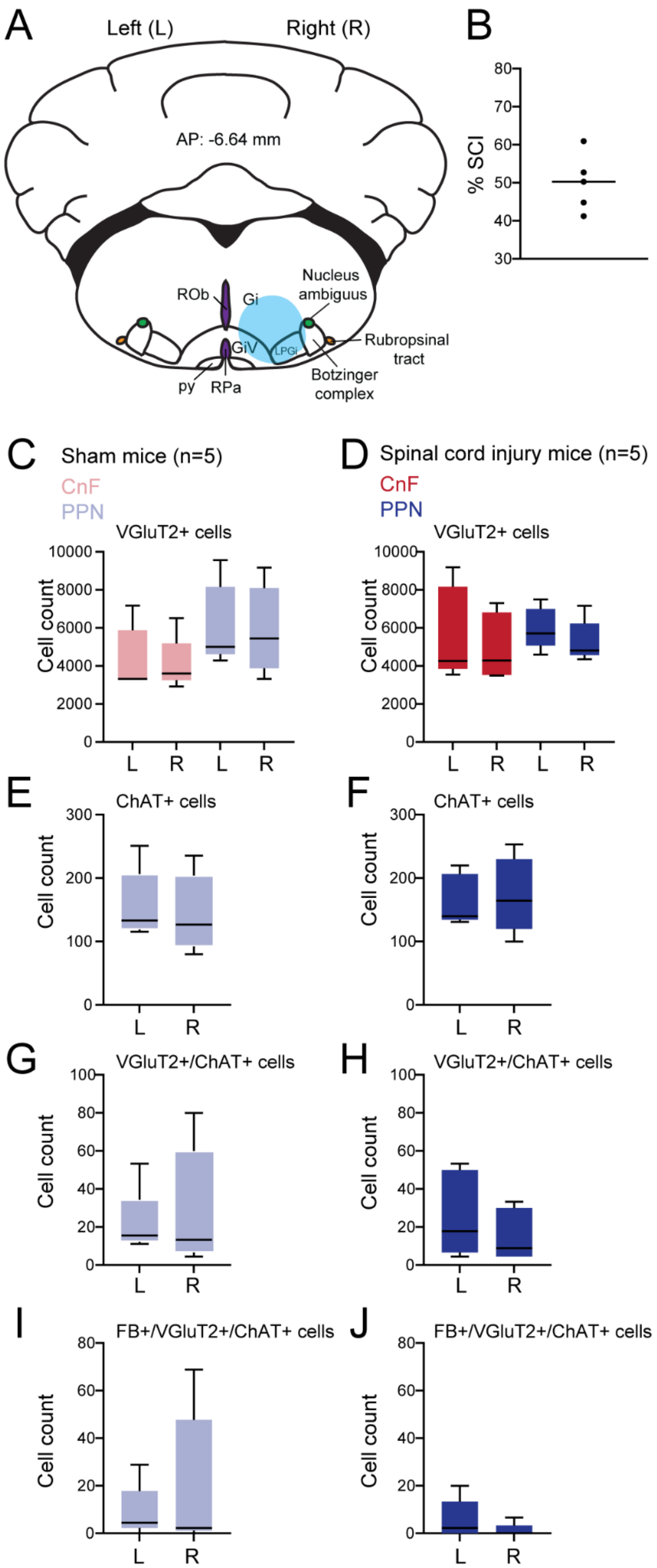

A
Left (L)
Right (R)
AP: -6.64 mm
ROb
Gi
Nucleus ambiguus
GiV
LPGi
Rubropsinal tract
py
RPa
Botzinger complex
B
% SCI
C
Sham mice (n=5)
CnF
PPN
VGluT2+ cells
Cell count
D
Spinal cord injury mice (n=5)
CnF
PPN
VGluT2+ cells
E
ChAT+ cells
F
ChAT+ cells
G
VGluT2+/ChAT+ cells
H
VGluT2+/ChAT+ cells
I
FB+/VGluT2+/ChAT+ cells
J
FB+/VGluT2+/ChAT+ cells
L
R

**Fig. S2: Cell counts of glutamatergic, cholinergic, and glutamatergic/cholinergic CnF and PPN neurons after chronic SCI. Related to Figure 2.**

(A) Fast Blue injections in the gigantocellular reticular nucleus (Gi), the lateral paragigantocellular reticular nucleus (LPGi), and the alpha and ventral pars of the gigantocellular reticular nucleus (GiV/α).

(B) Extent of the spinal cord injury.

(C-D) Number of VGluT2:cre+ neurons in left (L) ipsi- and right (R) contralesional CnF and PPN of Sham (C) and SCI (D) mice.

(E-F) Number of ChAT+ neurons in left (L) ipsi- and right (R) contralesional PPN of Sham (E) and SCI (F) mice.

(G-H) Number of VGluT2:cre+/ChAT+ neurons in left (L) ipsi- and right (R) contralesional PPN of Sham (G) and SCI (H) mice.

(I-J) Number of Fast-Blue+/VGluT2:cre+/ChAT+ neurons in left (L) ipsi- and right (R) contralesional PPN of Sham (I) and SCI (J) mice.

Mann-Whitney tests (C-J), L (left) vs R (right), $p > 0.05$.

Abbreviations: Gi: gigantocellular reticular nucleus, GiV: gigantocellular reticular nucleus ventral part, LPGi: lateral paragigantocellular nucleus, L: left, R: right, py: pyramidal tract, ROb: raphe obscurus nucleus, RPa: raphe pallidus nucleus.

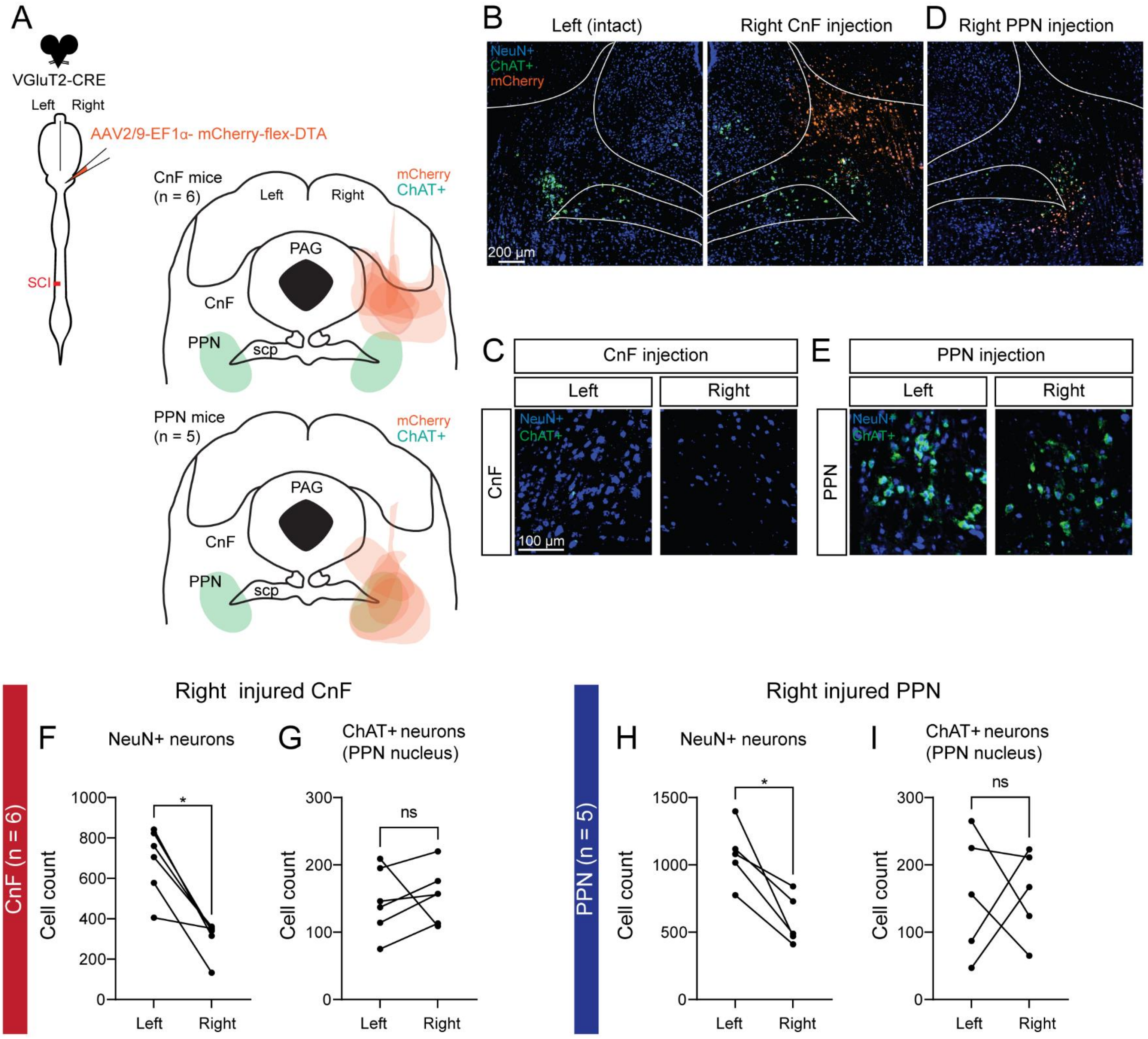


**Fig. S3: Genetic ablation of glutamatergic CnF or PPN neurons. Related to Figure 3.**

(A) Injection of AAV-EF1a-mCherry-flex-DTA in the right CnF or PPN of VGlut2-cre mice 9 weeks after SCI. Overlap of mCherry expression (orange), neuronal nuclear protein (NeuN in blue), and Choline AcetylTransferase (ChAT in green) staining.

(B) Low magnification images illustrating sections at the level of the left and right MLR nuclei upon genetic ablation of glutamatergic neurons of the right CnF. mCherry expression (orange) illustrates the extent of the AAV injection, NeuN (blue) staining identifies all neurons, and ChAT (green) staining identifies cholinergic PPN neurons.
(C) High magnification images of B with immunostaining against NeuN (blue) and ChAT (cholinergic neurons, green) at the level of the CnF.
(D) Low magnification image illustrating a section of right MLR nuclei upon genetic ablation of glutamatergic neurons of the right PPN.
(E) High magnification images of D with immunostaining against NeuN (blue) and ChAT (cholinergic neurons, green) at the level of the PPN.
(F) Number of NeuN positive neurons in the left and right CnF upon injection of the right CnF ($n = 6$ mice, one-tailed test, Wilcoxon matched-pairs signed rank test, $p = 0.02$).
(G) Number of cholinergic neurons in the left and right PPN upon injection of the right CnF ($n = 6$ mice, one-tailed test, Wilcoxon matched-pairs signed rank test, $p = 0.22$).
(H) Number of NeuN positive neurons in the left and right PPN upon injection of the right PPN ($n = 5$ mice, one-tailed test, Wilcoxon matched-pairs signed rank test, $p = 0.03$).
(I) Number of cholinergic neurons in the left and right PPN upon injection of the right PPN ($n = 5$ mice, one-tailed test, Wilcoxon matched-pairs signed rank test, $p = 0.5$).
Abbreviations: ChAT: Choline Acetyltransferase, CnF: Cuneiform nucleus, PPN: Pedunculopontine nucleus, PAG: Periaqueductal Gray, SCP: Superior Cerebellar Peduncle.

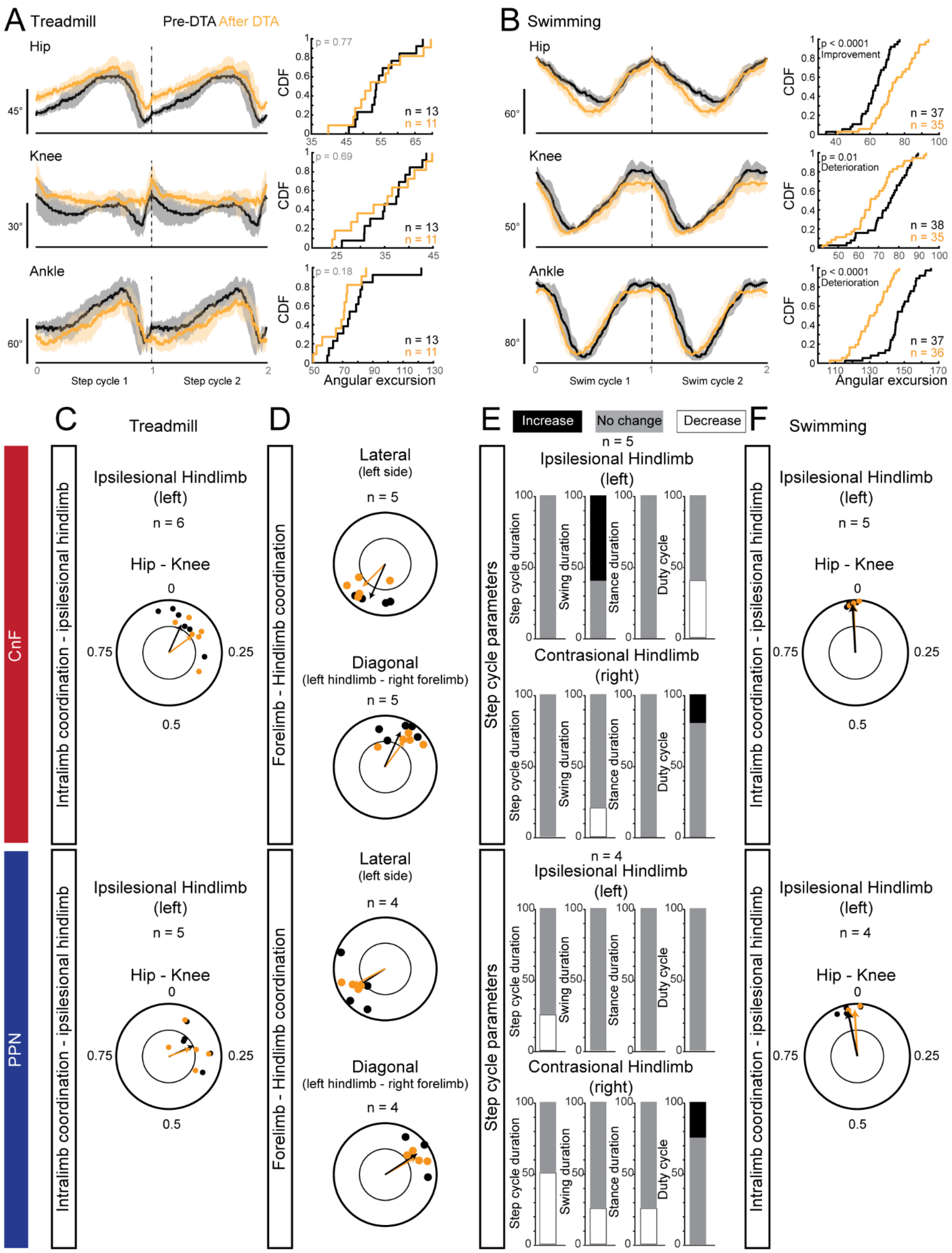

A Treadmill
Pre-DTA After DTA
Hip
Knee
Ankle
45°
30°
60°
Step cycle 1
Step cycle 2
CDF
p = 0.77
p = 0.69
p = 0.18
n = 13
n = 11
Angular excursion
B Swimming
60°
50°
80°
Swim cycle 1
Swim cycle 2
p < 0.0001
Improvement
n = 37
n = 35
p = 0.01
Deterioration
n = 38
n = 35
p < 0.0001
Deterioration
n = 37
n = 36
C Treadmill
CnF
PPN
Intralimb coordination - ipsilesional hindlimb
Ipsilesional Hindlimb (left)
n = 6
n = 5
Hip - Knee
0
0.25
0.5
0.75
D
Forelimb - Hindlimb coordination
Lateral (left side)
Diagonal (left hindlimb - right forelimb)
n = 5
n = 4
E
Increase
No change
Decrease
Step cycle parameters
Ipsilesional Hindlimb (left)
Contrasional Hindlimb (right)
n = 5
n = 4
Step cycle duration
Swing duration
Stance duration
Duty cycle
F Swimming

**Fig. S4: Intralimb coordination during locomotion upon genetic ablation. Related to Figure 3.**

(A) Mean and SD of hip, knee, and ankle joint angles of the ipsilesional hindlimb during treadmill locomotion before and after genetic ablation of glutamatergic PPN neurons in a mouse. Cumulative distribution function (CDF) of the angular excursion of hindlimb joints (n = step cycles, Mann-Whitney test or unpaired t-test according to the normality of the distribution).

(B) Mean and SD of hip, knee, and ankle joint angles of the ipsilesional hindlimb during swimming before and after genetic ablation of glutamatergic PPN neurons (same mouse as in A). Cumulative distribution function (CDF) of the angular excursion of hindlimb joints (n = swim cycles, Mann-Whitney test or unpaired t-test according to the normality of the distribution).

(C) Intralimb coupling between the hip and knee during treadmill locomotion before and after genetic ablation of glutamatergic neurons of the CnF or PPN. Each circle in polar plots represents a mouse.

(D) Lateral coupling between left fore-and hindlimb (anchored on the left forelimb) and diagonal coupling between left hindlimb and right forelimb (anchored on the right forelimb). Each circle in polar plots represents a mouse.

(E) Percentage of mice exhibiting a significant increase, decrease, or absence of change in step cycle duration, swing and stance phase duration, and duty cycle of the stance phase (Mann-Whitney test or unpaired t-test according to the normality of the distribution).

(F) Intralimb coupling between the hip and knee during swimming. Each circle in polar plots represents a mouse.

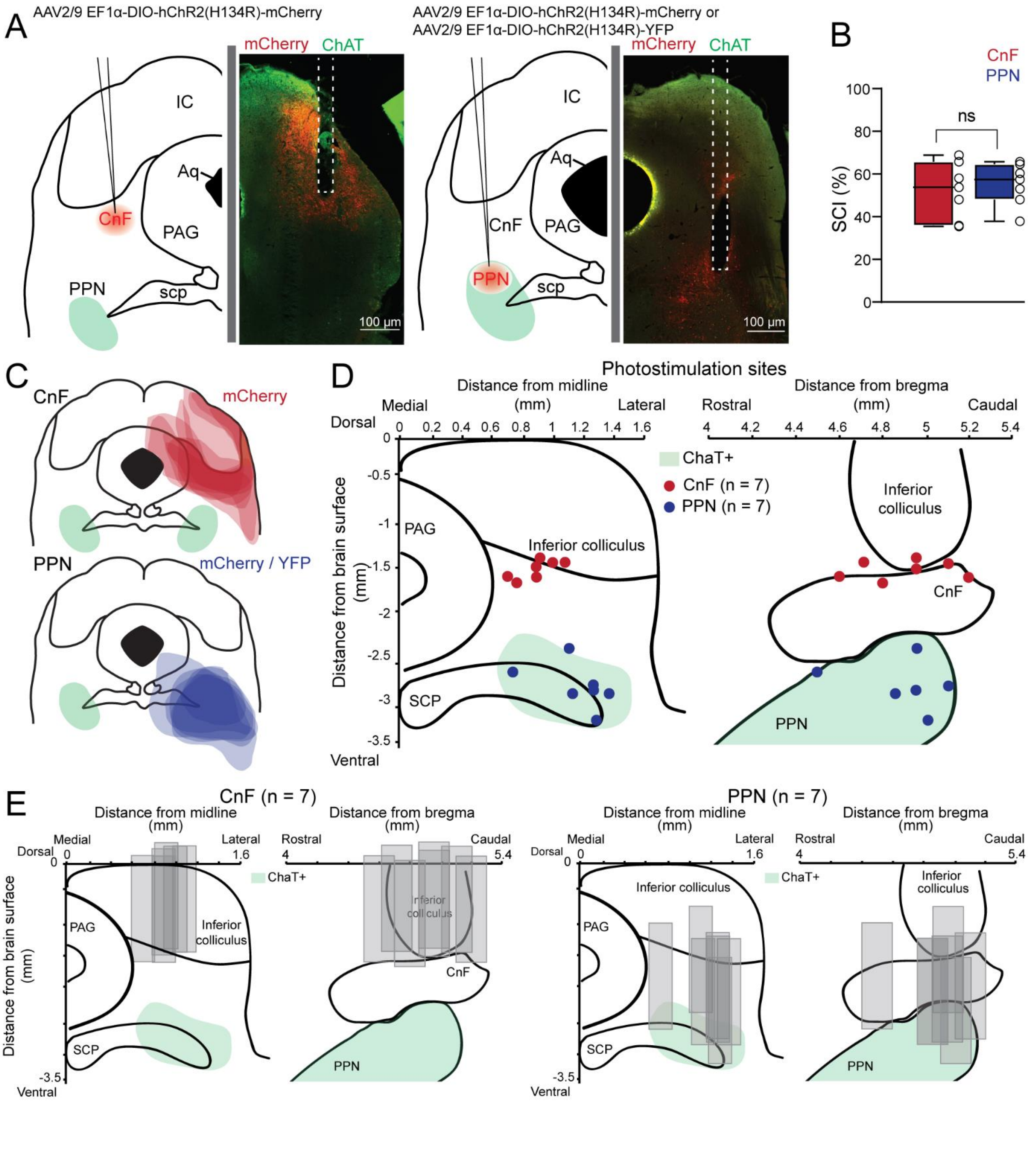

A
AAV2/9 EF1α-DIO-hChR2(H134R)-mCherry
mCherry ChAT
IC
Aq
CnF
PAG
PPN
scp
100 µm
AAV2/9 EF1α-DIO-hChR2(H134R)-mCherry or
AAV2/9 EF1α-DIO-hChR2(H134R)-YFP
B
CnF
PPN
SCI (%)
ns
C
mCherry
mCherry / YFP
D
Photostimulation sites
Distance from midline (mm)
Medial
Lateral
Dorsal
Ventral
Distance from brain surface (mm)
ChaT+
CnF (n = 7)
PPN (n = 7)
Inferior colliculus
SCP
Distance from bregma (mm)
Rostral
Caudal
E
CnF (n = 7)
PPN (n = 7)

**Fig. S5: Extent of the cre-lox recombination, site of optical cannulas, and extent of the lesion site. Related to Figure 4.**

(A) Examples of injection sites with AAV-EF1a-DiO-ChR2-mCherry/YFP in the CnF or PPN showing the extent of the cre-lox recombination with mCherry or YFP reporter expression.

(B) Lesion as percentage of the surface of the transverse section at the thoracic spinal level (n = 7 CnF and 7 PPN, unpaired t-test, p = 0.64).

(C) Mapping of injection spread. For each mouse (n = 7 CnF and 7 PPN), the infection site was identified and segmented as a series of coordinates forming contour maps. Maps were superimposed with overlapping areas showing a darker shade. Mice were injected with AAV-EF1-DiO-ChR2-mCherry or AAV-EF1-DiO-ChR2-YFP to target the ventral PPN (blue), and the CnF (red).

(D) Anatomical localization of the tip of the optical fibers at the level of the CnF and the caudal PPN.

(E) Anatomical fiber localization for the cohort presented in D. Fiber tips are represented for each mouse.

Abbreviations: Aq: Aqueduct, ChAT: Choline Acetyltransferase, CnF: Cuneiform nucleus, C: Inferior Colliculus, PPN: Pedunculopontine nucleus, PAG: Periaqueductal Gray, SCP: Superior Cerebellar Peduncle, YFP: Yellow Fluorescent Protein.

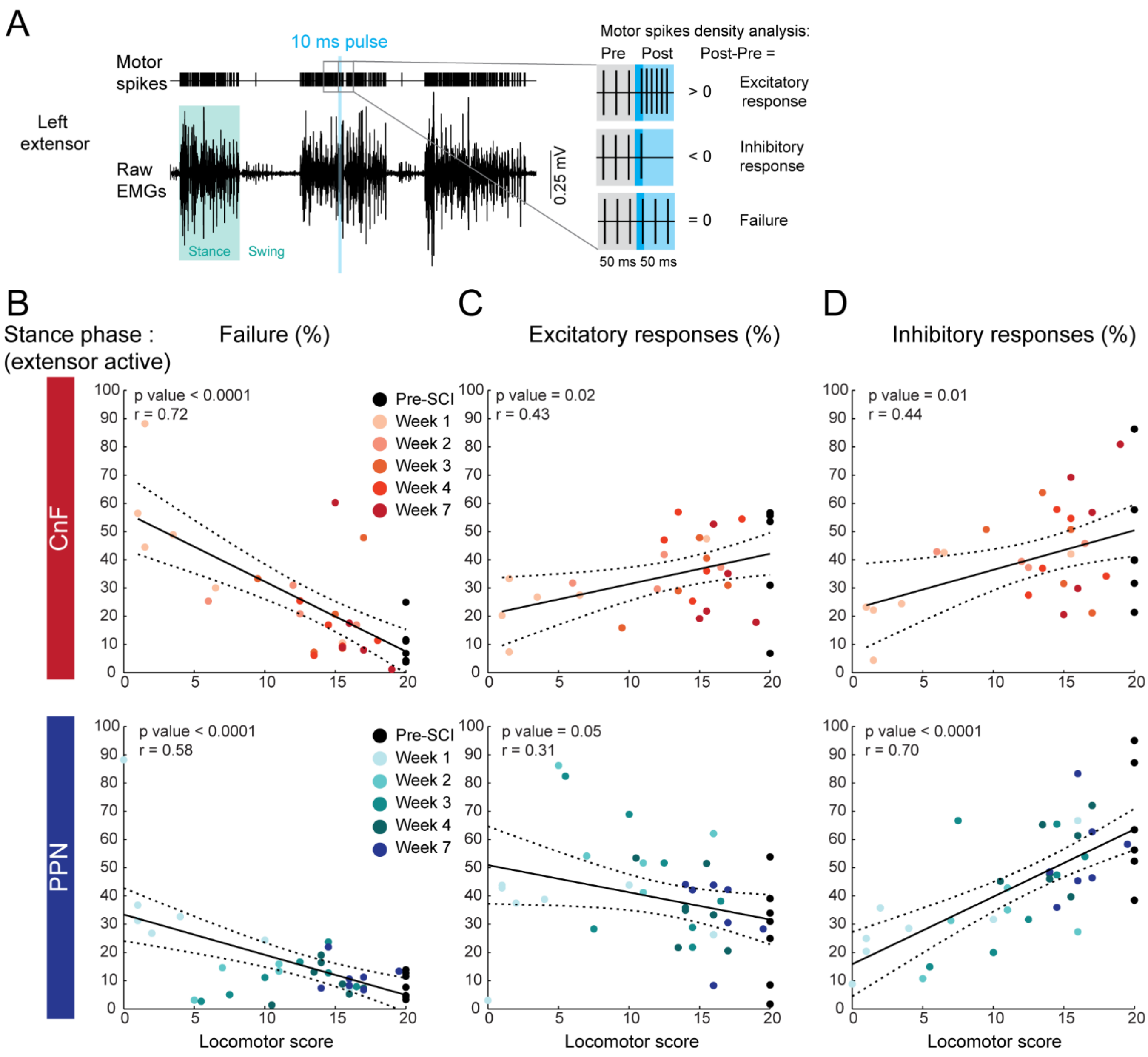


**Fig. S6: Changes in the proportion of failure, excitatory, and inhibitory motor responses in the ipsilesional extensor muscle during the stance phase after SCI. Related to Figure 4.**

(A) Example of changes in motor spike density upon photostimulation.

(B-D) Proportion of failure (B), excitatory (C), and inhibitory (D) motor responses evoked in the ipsilesional extensor during the stance phase as a function of the locomotor score of the ipsilesional hindlimb over the course of recovery after SCI (n = 6 CnF and n = 7 PPN per week, Linear regressions (solid lines) and 95% confidence interval (dashed lines)).

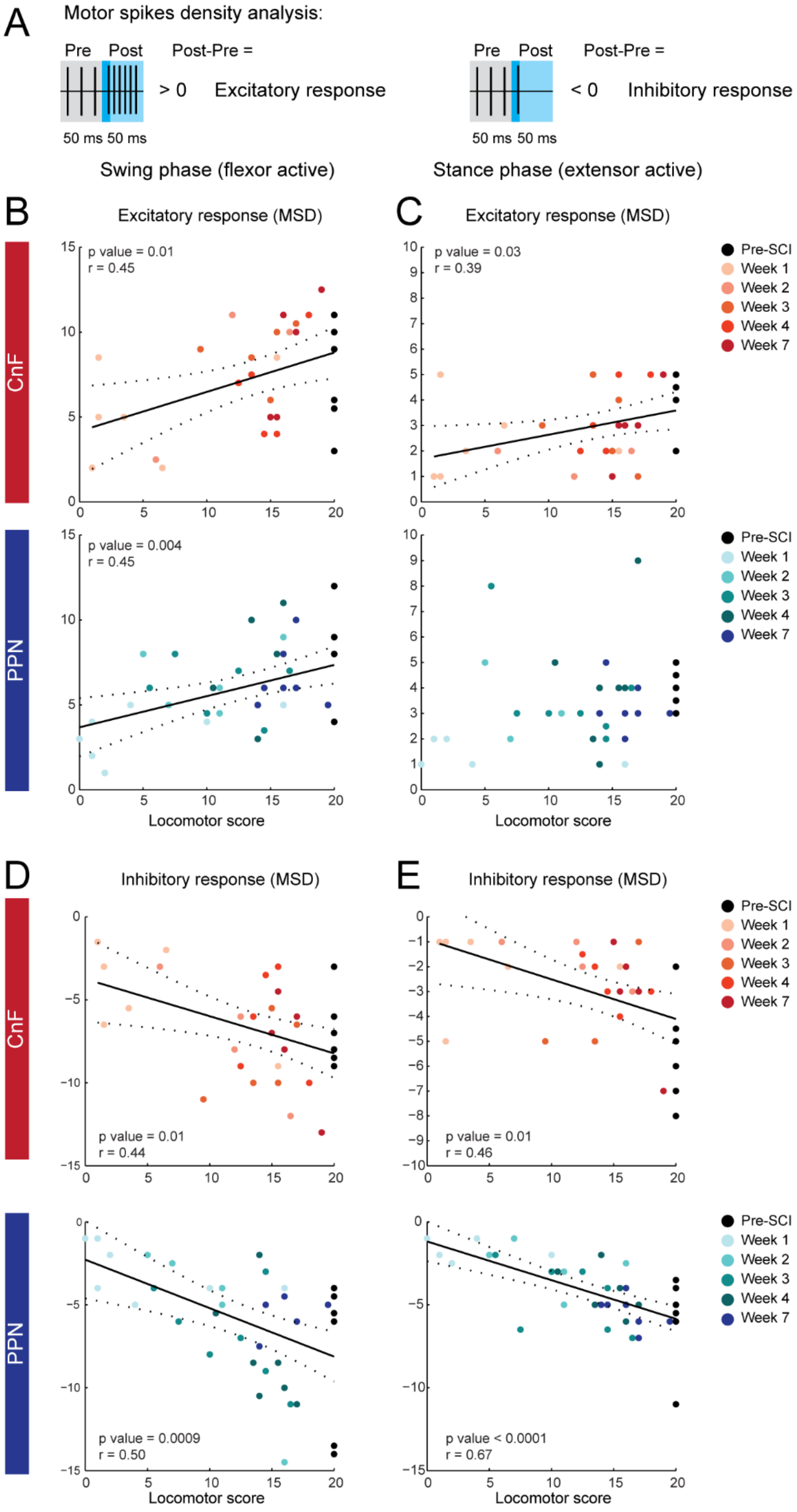

A
Motor spikes density analysis:
Pre Post Post-Pre =
> 0 Excitatory response
< 0 Inhibitory response
50 ms 50 ms
Swing phase (flexor active)
Stance phase (extensor active)
B
C
Excitatory response (MSD)
CnF
PPN
p value = 0.01
r = 0.45
p value = 0.03
r = 0.39
p value = 0.004
r = 0.45
Pre-SCI
Week 1
Week 2
Week 3
Week 4
Week 7
Locomotor score
D
E
Inhibitory response (MSD)
p value = 0.01
r = 0.44
p value = 0.01
r = 0.46
p value = 0.0009
r = 0.50
p value < 0.0001
r = 0.67

**Fig. S7: Changes in motor spike density of excitatory and inhibitory motor responses evoked in the ipsilesional hindlimb before and after SCI. Related to Figure 4.**

(A) Example of changes in motor spike density upon photostimulation.

(B) Motor spike density of excitatory motor responses evoked in the ipsilesional flexor muscle during the swing phase upon short pulse photostimulation delivered above glutamatergic CnF or PPN neurons as a function of the locomotor score of the ipsilesional hindlimb over the course of recovery after SCI (n = 6 CnF and n = 7 PPN per week, Linear regressions (solid lines) and 95% confidence interval (dashed lines)).

(C) Motor spike density of excitatory motor responses evoked in the ipsilesional extensor muscle during the stance phase upon short pulse photostimulation delivered above glutamatergic CnF or PPN neurons as a function of the locomotor score of the ipsilesional hindlimb over the course of recovery (n = 6 CnF and n = 7 PPN per week).

(D) Motor spike density of inhibitory responses evoked in the ipsilesional flexor muscle during the swing phase upon short-pulse photostimulation of glutamatergic CnF or PPN neurons as a function of locomotor score (n = 6 CnF and n = 7 PPN per week).

(E) Motor spike density of inhibitory responses evoked in the ipsilesional extensor muscle during the stance phase upon short-pulse photostimulation of glutamatergic CnF or PPN neurons as a function of locomotor score (n = 6 CnF and n = 7 PPN per week).

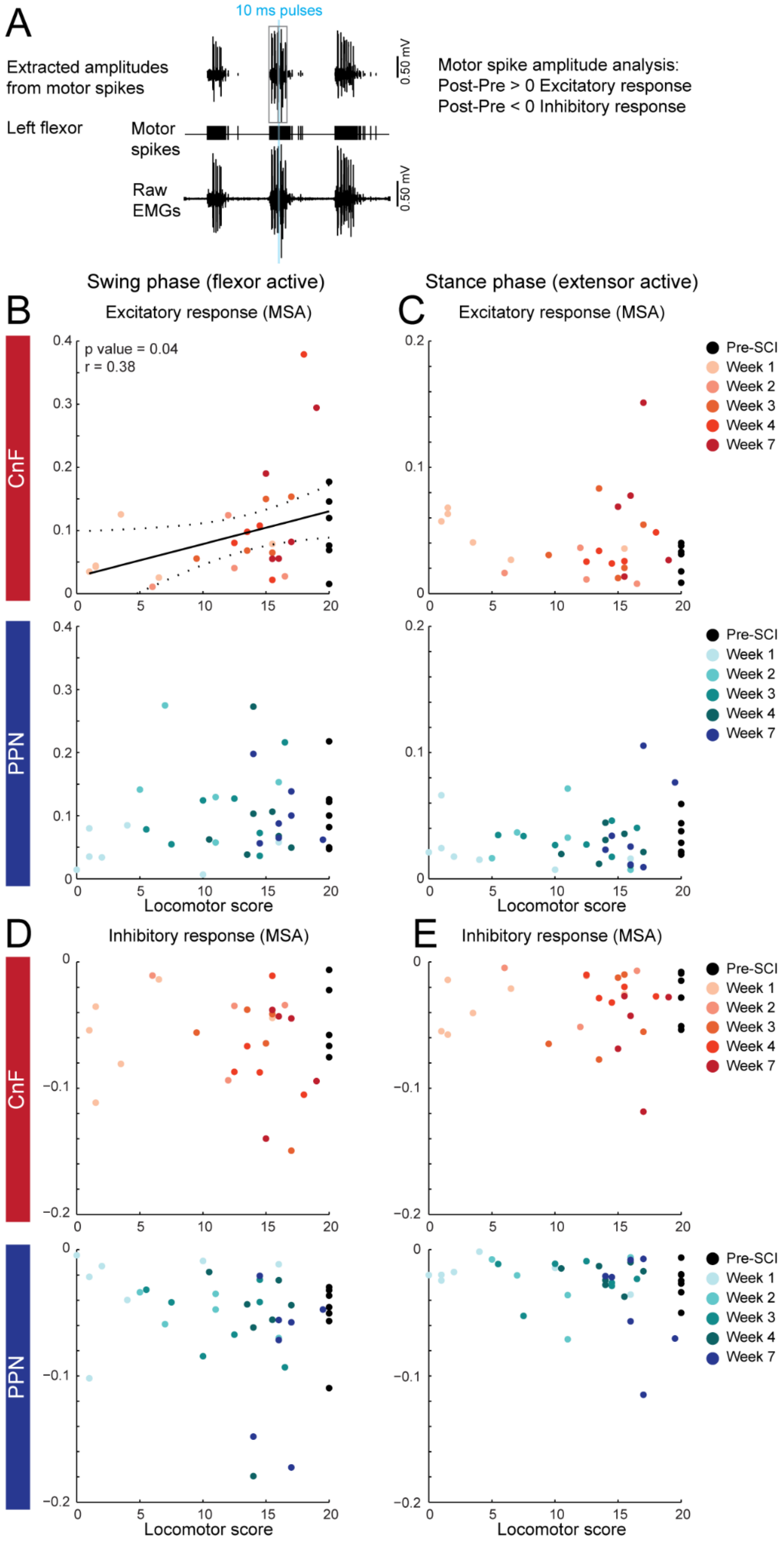
A
10 ms pulses
Extracted amplitudes from motor spikes
0.50 mV
Motor spike amplitude analysis:
Post-Pre > 0 Excitatory response
Post-Pre < 0 Inhibitory response
Left flexor
Motor spikes
Raw EMGs
Swing phase (flexor active)
Stance phase (extensor active)
B
Excitatory response (MSA)
C
Excitatory response (MSA)
p value = 0.04
r = 0.38
CnF
PPN
Pre-SCI
Week 1
Week 2
Week 3
Week 4
Week 7
Locomotor score
D
Inhibitory response (MSA)
E
Inhibitory response (MSA)

**Fig. S8: Changes in motor spike amplitude of excitatory and inhibitory motor responses evoked in the ipsilesional hindlimb before and after SCI. Related to Figure 4.**

(A) Example of changes in motor spike amplitude upon photostimulation.

(B) Motor spike amplitude of excitatory responses evoked in the ipsilesional flexor muscle during the swing phase upon short-pulse photostimulation of glutamatergic CnF or PPN neurons as a function of locomotor score (n = 6 CnF and n = 7 PPN per week, linear regression (solid line) and 95% confidence interval (dashed lines)).

(C) Motor spike amplitude of excitatory responses evoked in the ipsilesional extensor muscle during the stance phase upon short-pulse photostimulation of glutamatergic CnF or PPN neurons as a function of locomotor score (n = 6 CnF and n = 7 PPN per week).

(D) Motor spike amplitude of inhibitory responses evoked in the ipsilesional flexor muscle during the swing phase upon short-pulse photostimulation of glutamatergic CnF or PPN neurons as a function of locomotor score (n = 6 CnF and n = 7 PPN per week).

(E) Motor spike amplitude of inhibitory responses evoked in the ipsilesional extensor muscle during the stance phase upon short-pulse photostimulation of glutamatergic CnF or PPN neurons as a function of locomotor score (n = 6 CnF and n = 7 PPN per week).

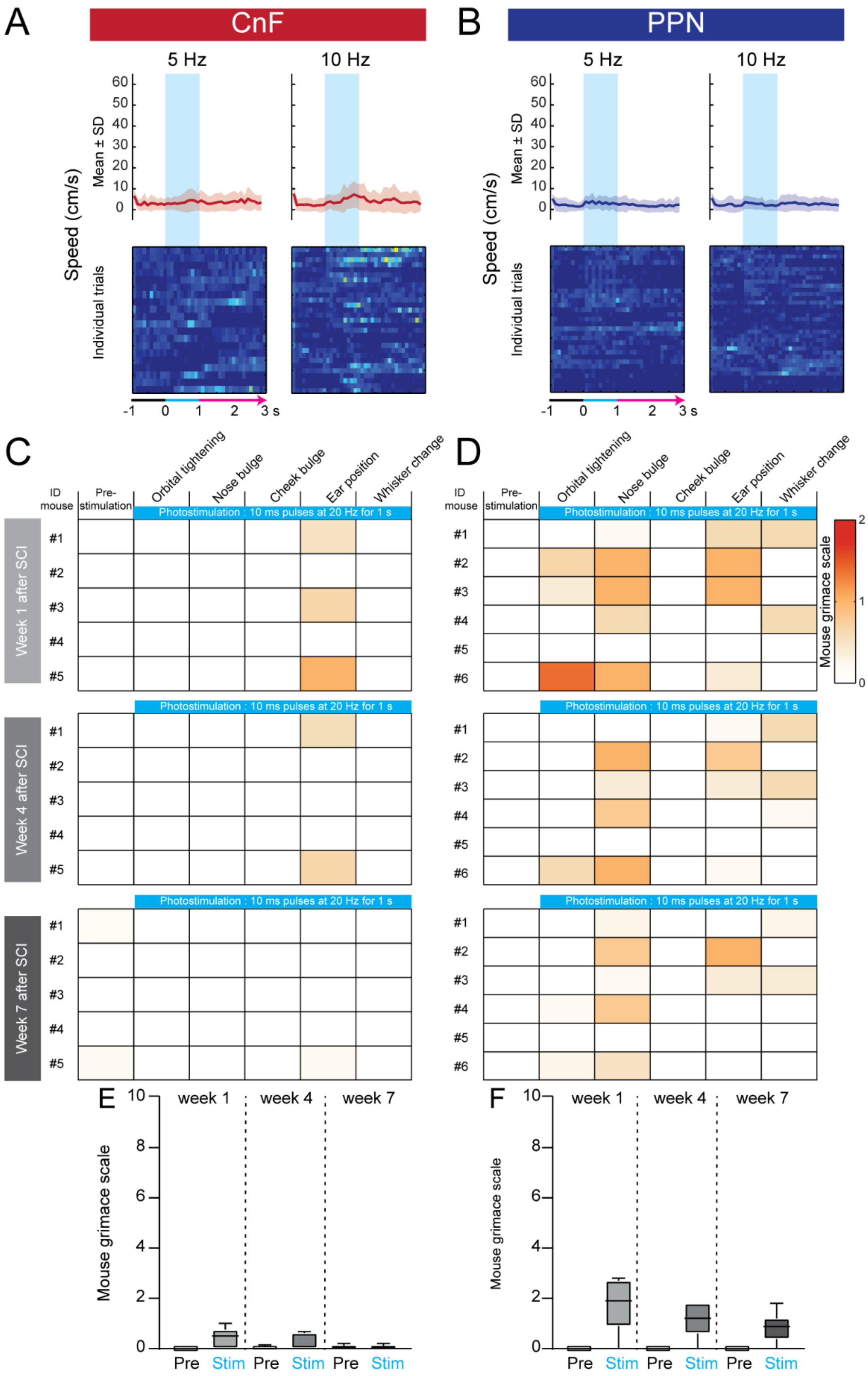
A
CnF
5 Hz
10 Hz
Speed (cm/s)
Mean ± SD
Individual trials
B
PPN
C
D
ID mouse
Pre-stimulation
Orbital tightening
Nose bulge
Cheek bulge
Ear position
Whisker change
Photostimulation : 10 ms pulses at 20 Hz for 1 s
Week 1 after SCI
Week 4 after SCI
Week 7 after SCI
Mouse grimace scale
E
F
week 1
week 4
week 7
Pre
Stim

**Fig. S9: No locomotor initiation upon low frequency photostimulation after chronic SCI and no reliable signs of stress or pain upon 20 Hz photostimulation after SCI. Related to Figure 5.**

(A-B) Mean and SD of locomotor speed evoked upon 1 s trains of photostimulation of glutamatergic neurons of the CnF (A, n = 5) or PPN (B, n = 7) at 5 and 10 Hz 8 weeks after SCI. Color-coded matrices represent individual trials (A, 5 Hz: 20 trials, 10 Hz: 29 trials; B, 5 Hz: 31 trials, 10 Hz: 35 trials; 100 ms bins). Unlike 20 and 50 Hz, 5 and 10 Hz photostimulation of glutamatergic CnF neurons failed to initiate locomotor bouts.

(C-D) Grimace scale features scored prior and during photostimulation of glutamatergic neurons of the CnF (C) or PPN (D) at 20 Hz at 1, 4, and 7 weeks after SCI at rest.

(E-F) Average mouse grimace scale evoked by stimulation of the CnF (E) and PPN (F) (Pre versus stim / week / group, Wilcoxon matched-pairs signed rank tests, $p > 0.05$).

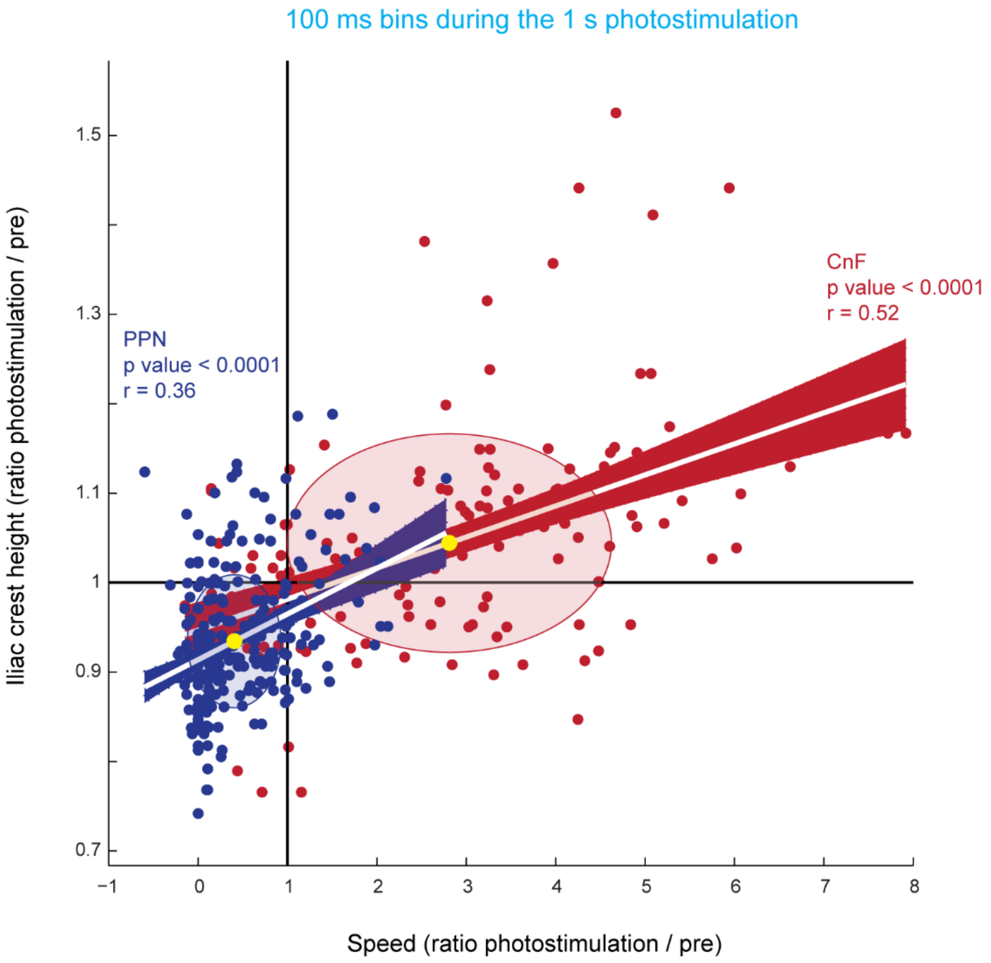


**Fig. S10: Speed and posture upon 1 s trains of photostimulation of glutamatergic CnF versus PPN neurons after chronic SCI. Related to Figure 6.**

Iliac crest height as a function of speed normalized on pre-stimulation data. 1 s photostimulation period was divided in 100 ms bins (10 bins). Each circle represents a bin, a tenth of trial. Significant linear regressions (white line, 95% confidence interval in red or blue) between both parameters upon photostimulation of the CnF or PPN (n = 150 bins for 4 CnF mice and 280 bins for 7 PPN mice, 100 ms bins). Yellow circles represent the mean of the iliac crest height and speed ratios; ellipses represent interpolation of the SD in the 2-dimensions. Negative ratios for speed represent rare cases of backward locomotion.

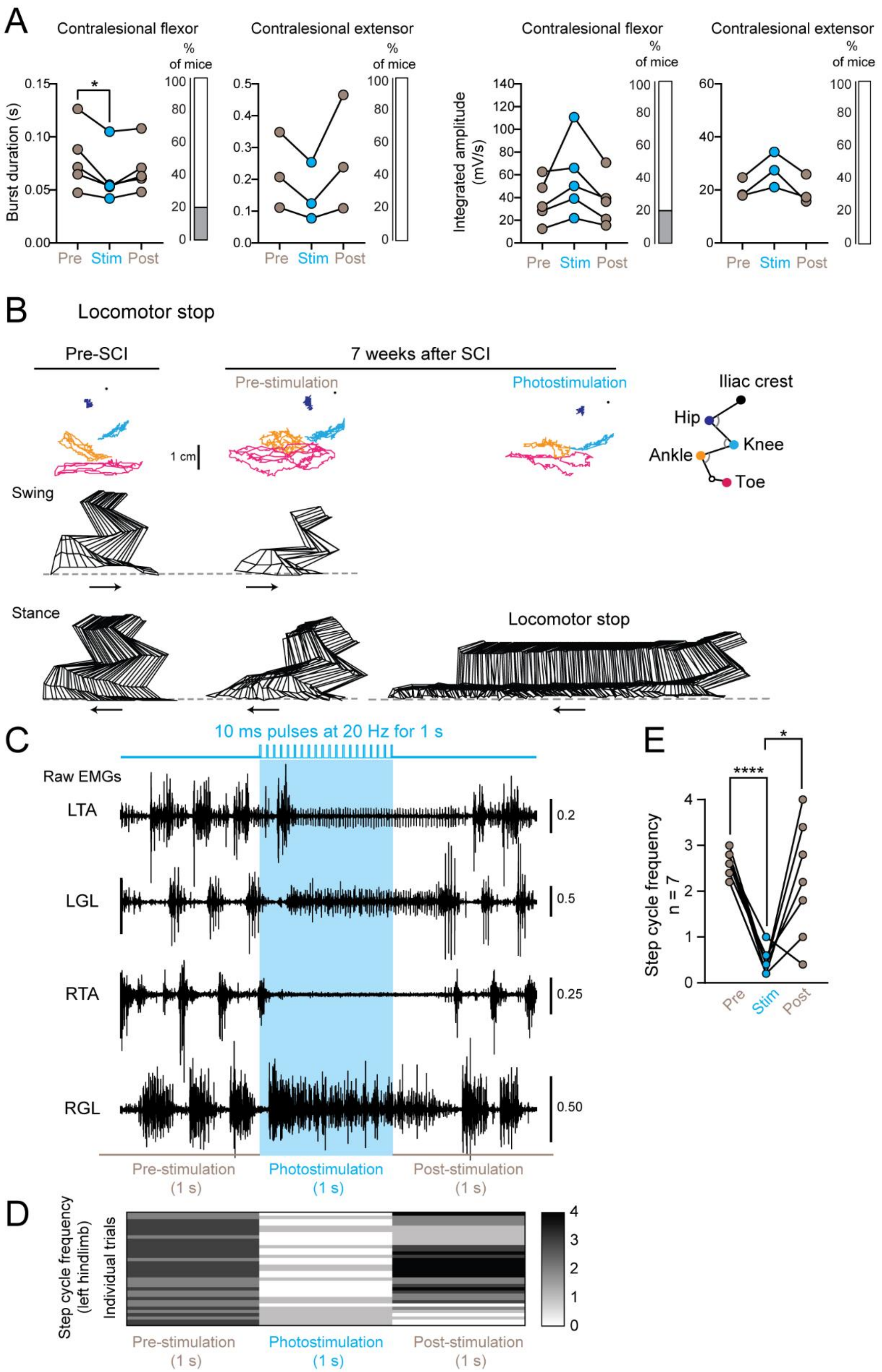
A
Contralesional flexor
Contralesional extensor
Burst duration (s)
Integrated amplitude (mV/s)
% of mice
Pre
Stim
Post
B
Locomotor stop
Pre-SCI
7 weeks after SCI
Pre-stimulation
Photostimulation
1 cm
Iliac crest
Hip
Knee
Ankle
Toe
Swing
Stance
C
10 ms pulses at 20 Hz for 1 s
Raw EMGs
LTA
LGL
RTA
RGL
0.2
0.5
0.25
0.50
Pre-stimulation (1 s)
Photostimulation (1 s)
Post-stimulation (1 s)
D
Step cycle frequency (left hindlimb)
Individual trials
E
Step cycle frequency n = 7
****
*

**Fig. S11: 1 s trains of photostimulation of glutamatergic CnF or PPN neurons after chronic SCI during treadmill locomotion. Related to Figure 7.**

(A) 1 s trains of photostimulation of glutamatergic CnF neurons after chronic SCI. Burst duration and integrated burst amplitude of background EMG activity in contralesional right flexor and extensor muscles (n = 5 for the contralesional flexor and n = 3 for the contralesional extensor, Friedman test [p = 0.009] with Dunn's multiple comparison post hoc test, *p < 0.05). Percentage of mice showing a significant decrease (i.e., improvement in white) or no change (gray) in the burst duration and percentage of mice showing a significant increase (i.e., improvement in white) or no change (gray) in the integrated amplitude (n = 5 for the contralesional flexor and n = 3 for the contralesional extensor, Mann-Whitney test or unpaired t-test according to the normality of the distribution).

(B-E) 1 s trains of photostimulation of glutamatergic PPN neurons after chronic SCI. (B) Cyclic trajectories of hindlimb joints (anchored on the iliac crest) during locomotion before and after chronic SCI (same PPN mouse). Stick diagrams illustrate the swing and stance phases (arrows show the direction of movement). (C) Raw EMG recordings upon a 1 s train of photostimulation. (D) Step cycle frequency colormap of pre/stim/post periods of 35 trials (7 mice, 5 trials/mouse). (E) Mean step cycle frequency of each mouse (n = 7, repeated measures one-way ANOVA [p = 0.0065] with Tukey's multiple comparisons test, ****p < 0.0001, *p < 0.05).

Abbreviations: see Figure 1 legend.

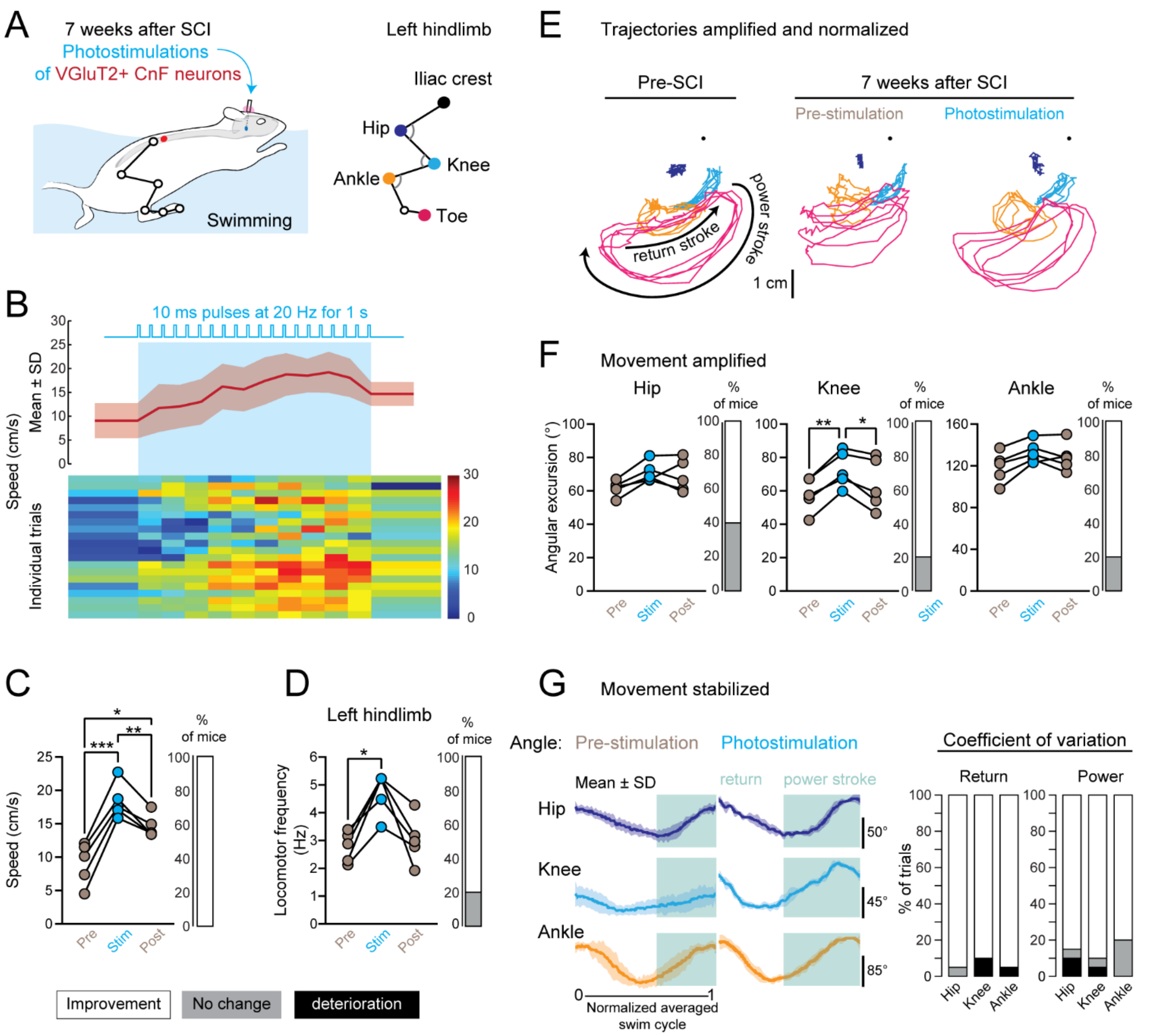


**Fig. S12: Functional improvement of swimming upon long trains of photostimulation of glutamatergic CnF neurons after chronic SCI. Related to Figure 7.**

(A) Kinematic analysis of the ipsilesional hindlimb evoked upon long trains of photostimulation (10 ms at 20 Hz for 1 s) of glutamatergic CnF neurons during swimming 7 weeks after SCI (n = 5 mice, 4 trials per mouse).

(B) Mean and SD of the swimming and individual trials color-coded as a function of speed (100 ms bins, 4 trials per mouse).

(C) Mean swimming speed (500 ms pre/stim/post periods, last 500 ms of the 1 s stimulation; repeated measures one-way ANOVA [$p = 0.0004$] with Tukey's multiple comparisons test, *$p < 0.05$, **$p < 0.01$, ***$p < 0.001$). 100% of mice exhibited increased speed upon photostimulation (Mann-Whitney test or unpaired t-test according to the normality of the distribution).

(D) Mean swimming frequency upon photostimulation (Friedman test [$p = 0.02$] with Dunn's multiple comparisons test, *$p < 0.05$). Percentage of mice exhibiting increased locomotor frequency upon photostimulation (80%, Mann-Whitney test or unpaired t-test according to the normality of the distribution).

(E) Trajectories of the hip, knee, ankle, and toe anchored to the iliac crest during a swimming bout before and 7 weeks after SCI.

(F) Mean angular excursion of the ipsilesional hip, knee, and ankle (knee: repeated measures one-way ANOVA [$p = 0.004$] with Tukey's multiple comparisons test, *$p < 0.05$, **$p < 0.01$). Percentage of mice exhibiting a significant increase in their angular excursion upon photostimulation (60% for the hip and 80% for the knee and ankle; Mann-Whitney test or unpaired t-test according to the normality of the distribution).

(G) Mean and SD of joint angles during normalized swim cycles upon photostimulation (512 bins). Percentage of trials ($n = 20$) exhibiting a significant decrease in coefficients of variation (i.e., improvement) of joint angles during return and power strokes upon photostimulation (pre bins vs. stim, Mann-Whitney test or unpaired t-test according to the normality of the distribution).

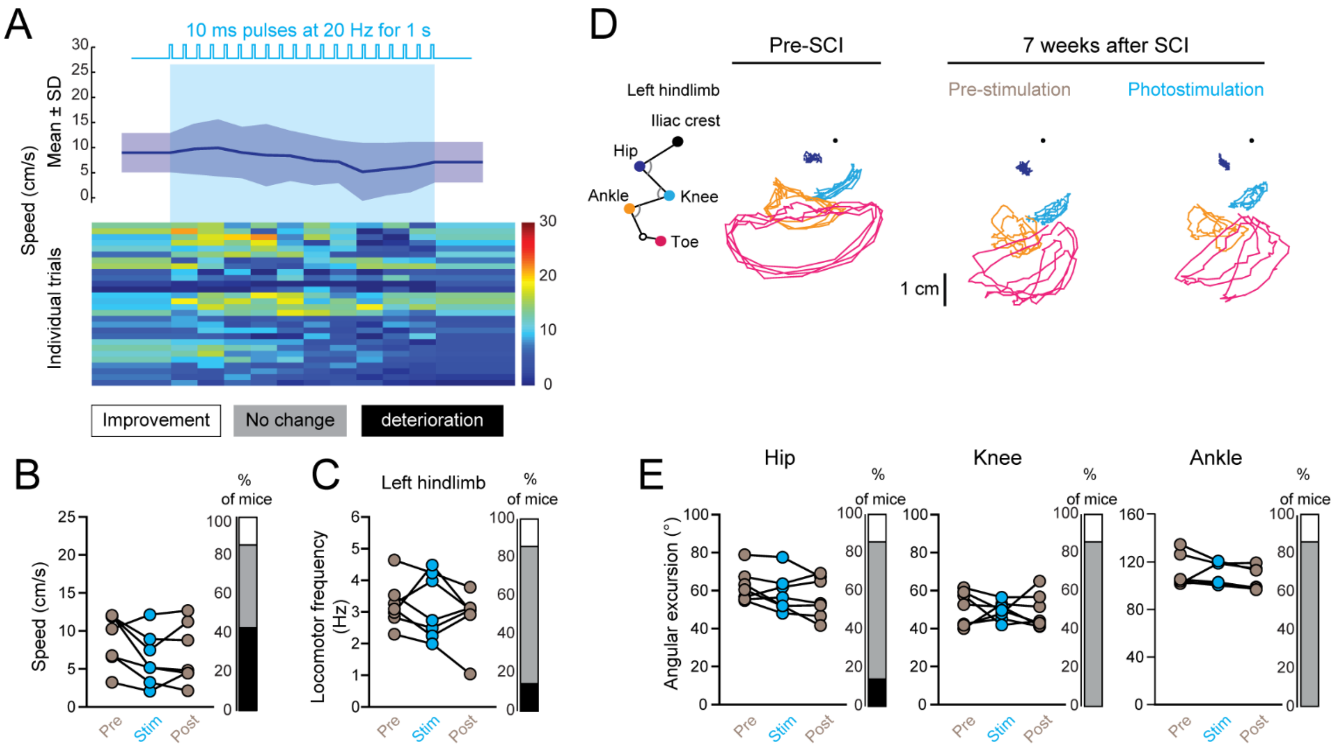


**Fig. S13: 1 s trains of photostimulation of glutamatergic PPN neurons failed to improve swimming after chronic SCI. Related to Figure 7.**

(A) Mean and SD of the swimming speed and individual trials color-coded as a function of speed (n = 7 mice, 100 ms bins, 4 trials per mouse).

(B) Mean swimming speed (500 ms pre/stim/post periods, last 500 ms of the 1 s stimulation. Percentage of mice exhibiting an increase (i.e., improvement) or decrease (i.e., deterioration) in speed upon photostimulation (Mann-Whitney test or unpaired t-test according to the normality of the distribution).

(C) Mean swimming frequency upon photostimulation. Percentage of mice exhibiting an increase (i.e., improvement) or decrease (i.e., deterioration) in locomotor frequency upon photostimulation (Mann-Whitney test or unpaired t-test according to the normality of the distribution).

(D) Trajectories of the hip, knee, ankle, and toe anchored to the iliac crest during a swimming bout before and 7 weeks after SCI.

(E) Mean angular excursion of the ipsilesional hip, knee, and ankle. Percentage of mice exhibiting a significant increase (i.e., improvement) or decrease (i.e., deterioration), or absence of changes in their angular excursion upon photostimulation (Mann-Whitney test or unpaired t-test according to the normality of the distribution).
