# Supplementary material for: Functional contribution of mesencephalic locomotor region nuclei to locomotor recovery after spinal cord injury
Source: Cell Rep Med. 2023 Feb 21;4(2):100946. doi: 10.1016/j.xcrm.2023.100946 (PMC9975330; doi:10.1016/j.xcrm.2023.100946)
Supplement: Document S2. Article plus supplemental information [file mmc2.pdf]

Cell Reports Medicine

Article

Functional contribution of mesencephalic locomotor region nuclei to locomotor recovery after spinal cord injury

Graphical abstract

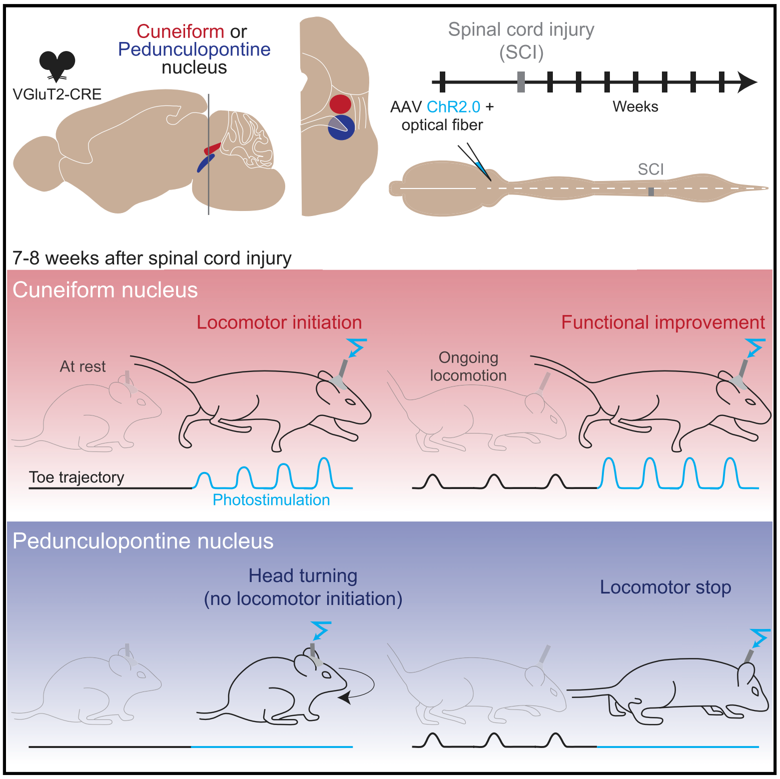

Highlights

- Glutamatergic CnF is more important than PPN to spontaneous motor recovery
- Glutamatergic CnF contributes more efficiently than PPN to spontaneous recovery
- Only activation of glutamatergic CnF initiates locomotion after chronic SCI
- Only activation of glutamatergic CnF improves stepping ability after chronic SCI

Authors

Marie Roussel, David Lafrance-Zoubga, Nicolas Josset, Maxime Lemieux, Frederic Bretzner

Correspondence

frederic.bretzner.1@ulaval.ca

In brief

Roussel et al. characterize the distinct contribution of midbrain neurons to locomotor recovery after spinal cord injury (SCI). Although both glutamatergic cuneiform nucleus (CnF) and pedunculopontine nucleus contribute to spontaneous motor recovery, only activation of glutamatergic CnF initiates locomotion and improves stepping ability after chronic SCI.

Roussel et al., 2023, Cell Reports Medicine *4*, 100946
February 21, 2023 © 2023 The Author(s).
https://doi.org/10.1016/j.xcrm.2023.100946

CellPress

Cell Reports Medicine

CellPress
OPEN ACCESS

Article

Functional contribution of mesencephalic locomotor region nuclei to locomotor recovery after spinal cord injury

Marie Roussel,[1] David Lafrance-Zoubga,[1] Nicolas Josset,[1] Maxime Lemieux,[1] and Frederic Bretzner[1,2,3,*]
[1]Centre de Recherche du CHU de Québec, CHUL-Neurosciences, 2705 Boul. Laurier, Québec, QC G1V 4G2, Canada
[2]Faculty of Medicine, Department of Psychiatry and Neurosciences, Université Laval, Québec, QC G1V 4G2, Canada
[3]Lead contact
*Correspondence: frederic.bretzner.1@ulaval.ca
https://doi.org/10.1016/j.xcrm.2023.100946

SUMMARY

Spinal cord injury (SCI) results in a disruption of information between the brain and the spinal circuit. Electrical stimulation of the mesencephalic locomotor region (MLR) can promote locomotor recovery in acute and chronic SCI rodent models. Although clinical trials are currently under way, there is still debate about the organization of this supraspinal center and which anatomic correlate of the MLR should be targeted to promote recovery. Combining kinematics, electromyographic recordings, anatomic analysis, and mouse genetics, our study reveals that glutamatergic neurons of the cuneiform nucleus contribute to locomotor recovery by enhancing motor efficacy in hindlimb muscles, and by increasing locomotor rhythm and speed on a treadmill, over ground, and during swimming in chronic SCI mice. In contrast, glutamatergic neurons of the pedunculopontine nucleus slow down locomotion. Therefore, our study identifies the cuneiform nucleus and its glutamatergic neurons as a therapeutical target to improve locomotor recovery in patients living with SCI.

INTRODUCTION

Although the spinal cord contains all the circuitry necessary for locomotion, people with spinal cord injury (SCI) are unable to walk due to the absence of commands from the brain. Motor recovery can be partially achieved by rehabilitative training and neuromodulatory therapies intended to promote the descending motor command from the brain to the spinal cord after SCI.[1–7] Recently, deep brain stimulation of the mesencephalic locomotor region (MLR), a supraspinal locomotor center, has been shown to improve locomotor functions in rats with chronic but incomplete SCI with even a few spared axonal fibers.[6–8] Interestingly, these functional changes come with an extensive reorganization in the brainstem region after SCI,[9] thus supporting the important contribution of the MLR to locomotor recovery after incomplete SCI.

The anatomic correlate of this functional region has been initially identified as the cuneiform nucleus (CnF), a cluster of glutamatergic neurons, and the pedunculopontine nucleus (PPN), a cluster of glutamatergic and cholinergic neurons. Despite a growing body of evidence from mouse genetics studies,[10–18] there is still debate about the exact anatomic correlate of this supraspinal locomotor center. Previously, using a head-restrained mouse on an air-lifted ball,[19,20] it was shown that optogenetic stimulation of glutamatergic neurons of the MLR (including the CnF and PPN) can generate locomotion in contrast to cholinergic or GABAergic MLR neurons. More recently, using smaller volumes of adeno-associated virus to circumscribe photostimulation to a nucleus of interest, it was shown that glutamatergic CnF neurons can initiate locomotion in freely behaving mice,[10,11,15] whereas glutamatergic PPN neurons exhibit higher variability in generating locomotion.[10,11,14–16] Furthermore, glutamatergic CnF neurons accelerate locomotor rhythm and speed during ongoing locomotion, whereas glutamatergic PPN neurons only prolong the stance phase, contributing to postural adjustments and slowing locomotor rhythm in normal conditions.[10,15] Although cholinergic PPN neurons were initially reported to increase speed during head-restrained locomotion,[20] more recent studies suggest that they do not actually modulate locomotor speed in freely behaving mice.[10,11] Although deep brain stimulation (DBS) in the PPN[7] or the CnF[8] can improve locomotor recovery in animal models of SCI, there are still questions about which neuronal population is the most efficient. With ongoing clinical trials aiming to assess DBS in the vicinity of the MLR of patients with incomplete SCI,[21] it is now urgent to gain a better understanding of how these distinct neuronal populations of the midbrain can contribute to and promote functional locomotor recovery after SCI.

We hypothesize here that glutamatergic neurons of the CnF and PPN contribute to spontaneous locomotor recovery after SCI and that selective activation of glutamatergic CnF, but not glutamatergic PPN neurons, can further improve locomotor functions. Combining detailed kinematics, electromyographic (EMG) recordings, anatomic analyses, and mouse genetics, we found that glutamatergic CnF neurons modulate locomotor pattern and rhythm and enhance motor efficacy in ipsilesional hindlimb muscles after SCI, in contrast to glutamatergic neurons of the PPN. As a therapeutical approach, we also found that 1-s

This is an open access article under the CC BY license (http://creativecommons.org/licenses/by/4.0/).

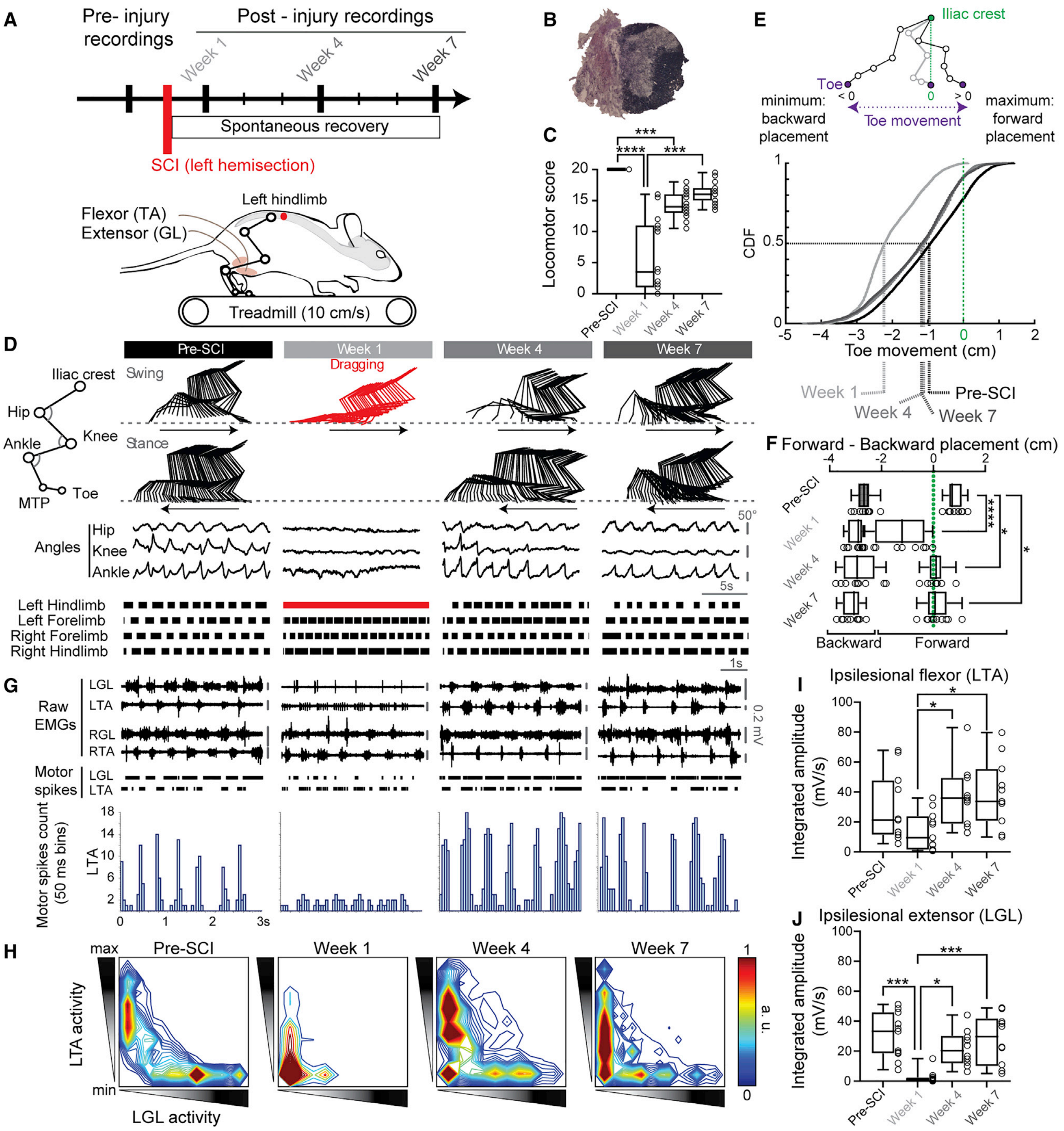

Figure 1. Spontaneous locomotor recovery after spinal cord injury (SCI)

(A) Kinematics and electromyographic (EMG) recordings during treadmill locomotion before and after SCI. GL, gastrocnemius lateralis; TA, tibialis anterior.

(B) Example of a hemisection at the left thoracic level (T8-T9) with cresyl violet and luxol Fast Blue staining.

(C) Locomotor score of the ipsilesional (left) hindlimb before and after SCI (n = 15 mice, Friedman test [$p < 0.0001$] with Dunn's multiple comparisons test, ****$p < 0.0001$; ***$p < 0.001$).

(D) Stick diagrams of the ipsilesional hindlimb during the swing and stance phase (arrows indicate movement direction), joint angles, and gait diagrams (bars represent the stance).

(E) Cumulative distribution function (CDF) of paw movement in reference to the iliac crest before and after SCI (n = 10 mice).

(F) Forward and backward paw placement in reference to the iliac crest (Pre-SCI vs. Week 1–7, n = 10 mice, one-way ANOVA [$p < 0.0001$] with Dunnett's multiple comparisons test, ****$p < 0.0001$, *$p < 0.05$).

(legend continued on next page)

trains of photostimulation delivered above glutamatergic CnF neurons promote functional recovery of initiation and locomotion after SCI, whereas glutamatergic PPN neurons slow down locomotion, thus identifying the CnF and its glutamatergic neuronal population as a neurosurgical target to promote functional locomotor recovery in patients with SCI.

RESULTS

Mice exhibit asymmetrical locomotor pattern following incomplete SCI

Adult mice underwent a lateral hemisection at the low thoracic level, abolishing supraspinal inputs from the brain on the left side of the lumbar spinal cord controlling hindlimb locomotion. Using kinematic and electromyographic recordings, we assessed spontaneous locomotor recovery after SCI (Figures 1 and S1). As previously reported in other animal species,[22–28] mice initially displayed a transient paralysis on the side of the SCI over the first week post-SCI (Figures 1C, 1D, 1G and 1H) with a decrease in toe movement and forward foot placement (Figures 1E and 1F) and in the motor activity of the flexor, which were co-activated with otherwise weak extensors (Figures 1G–1J). This likely contributed to hind-paw dragging during swing and a very short stance. Although this limb displayed some locomotor-like movements, there was loss of weight support and no plantar stepping. Eventually, within a few weeks after SCI, the ipsilesional limb (i.e., on the lesion side) increased its stance duration and extensor activity, in addition to exhibiting a better coordination between its flexor and extensor locomotor activities (Figures 1I and 1J). This in turn reduced hind-paw dragging during swing and improved plantar stepping ability (Figures 1D and 1G), thus contributing to locomotor recovery of the ipsilesional limb over time.

No anatomic reorganization of medullar projecting MLR neurons after SCI

Given that the medullary reticular formation relays MLR inputs during locomotion[29–31] and that SCI leads to an extensive reorganization of projections between midbrain and brainstem nuclei after SCI,[9] we hypothesized an asymmetrical reorganization of the connectivity between glutamatergic neurons of MLR nuclei and their postsynaptic medullary targets following incomplete SCI. To test this hypothesis, a retrograde tracer Fast Blue was injected stereotaxically in the contralesional medullary reticular formation of adult transgenic VGlut2-Cre mice 7 weeks after SCI or sham surgery (Figure 2A; mice with Fast Blue injections leaking in the ipsilesional brainstem were excluded from our analysis, Figure S2). We specifically targeted the gigantocellular reticular nucleus, the alpha and ventral portion of the gigantocellular reticular nucleus, and the lateral paragigantocellular nucleus, which are important to the motor command.[32–35] Combining immunohistochemistry and stereological techniques (Figure 2B), we identified and quantified the neurotransmitter phenotype (e.g., glutamatergic, cholinergic, or both glutamatergic/cholinergic) of retrogradely labeled neurons in ipsilesional (left) and contralesional (right) CnF and PPN nuclei.

Using the most ventral part of the fourth ventricle as a reference, our 3D and 2D reconstructions illustrate a bilateral and symmetrical organization of CnF and PPN nuclei according to their neurotransmitter phenotype (e.g., glutamatergic, cholinergic, or double) and their unilateral projection in the contralesional medullary reticular formation (Figures 2C–2E). We found a high density of medullary-projecting glutamatergic neurons in both left and right CnF and PPN, but very few cholinergic cells within the PPN (Figures 2C–2E), suggesting a bilateral organization of brainstem-projecting MLR populations in both sham and SCI mice. As previously reported,[36–39] we also found a few double glutamatergic/cholinergic neurons in the PPN (Figure S2). Nevertheless, in contrast to a previous SCI study,[9] the cell density profile and count of glutamatergic and cholinergic neurons of CnF or PPN nuclei of SCI animals were not significantly different from those of sham mice (Figures 2D–2G), suggesting that the organization of glutamatergic and cholinergic MLR neurons projecting to brainstem locomotor circuits was maintained after SCI.

Genetic deletion of glutamatergic neurons of the contralesional CnF or PPN impairs spontaneous locomotor recovery in chronic SCI mice

Given the functional role of the MLR to locomotor recovery after SCI,[6,8,9] we next evaluated the requirement of glutamatergic neurons of specific MLR nuclei to functional recovery (Figures 3, S3, and S4). To test this requirement, locomotor functions were assessed before and after conditional genetic ablation of glutamatergic neurons of the contralesional (right) CnF or PPN in chronic mice that had spontaneously recovered locomotor functions 8 weeks after left SCI (Figure 3A). We ensured that both CnF and PPN groups exhibited a similar behavioral recovery 8 weeks after SCI,[40] prior to genetic ablation of their glutamatergic neurons, and both groups showed the same extent of SCI on postmortem analysis (Figure 3B). As previously reported,[41,42] the cre-dependent virus (AAV-mCherry-flex-DTA) allowed us to visualize the extent of the injection site by mCherry expression in all neurons and to restrict the genetic ablation to Cre-expressing neurons (Figures S3A–S3E). In comparison with their contralateral control nuclei, there was a significant decrease of about 50% in NeuN-expressing neurons with no difference in the number of ChAT-expressing cholinergic neurons of the PPN (Figures S3F–S3I). During treadmill locomotion (Figures 3C–3E), genetic ablation of glutamatergic CnF neurons decreased the angular excursion of the ipsilesional hip in 60% of mice

(G) Raw EMGs and raster of motor spikes of flexor and extensor muscles. LGL, left gastrocnemius lateralis; LTA, left tibialis anterior; RGL: right gastrocnemius lateralis; RTA, right tibialis anterior.

(H) Flexor activity as a function of extensor activity of the ipsilesional hindlimb (n = 9 mice). Note the loss of alternation between flexor and extensor 1 week after SCI and its recovery associated with a longer flexor activity at 4 and 7 weeks after SCI.

(I) Integrated amplitude of the ipsilesional left flexor (n = 11 mice, Kruskal-Wallis test [$p = 0.0095$] with Dunn's multiple comparisons test, *$p < 0.05$).

(J) Integrated amplitude of the ipsilesional left extensor (n = 11 mice, Kruskal-Wallis test [$p < 0.0001$] with Dunn's multiple comparisons test, *$p < 0.05$, ***$p < 0.001$). See also Figure S1.

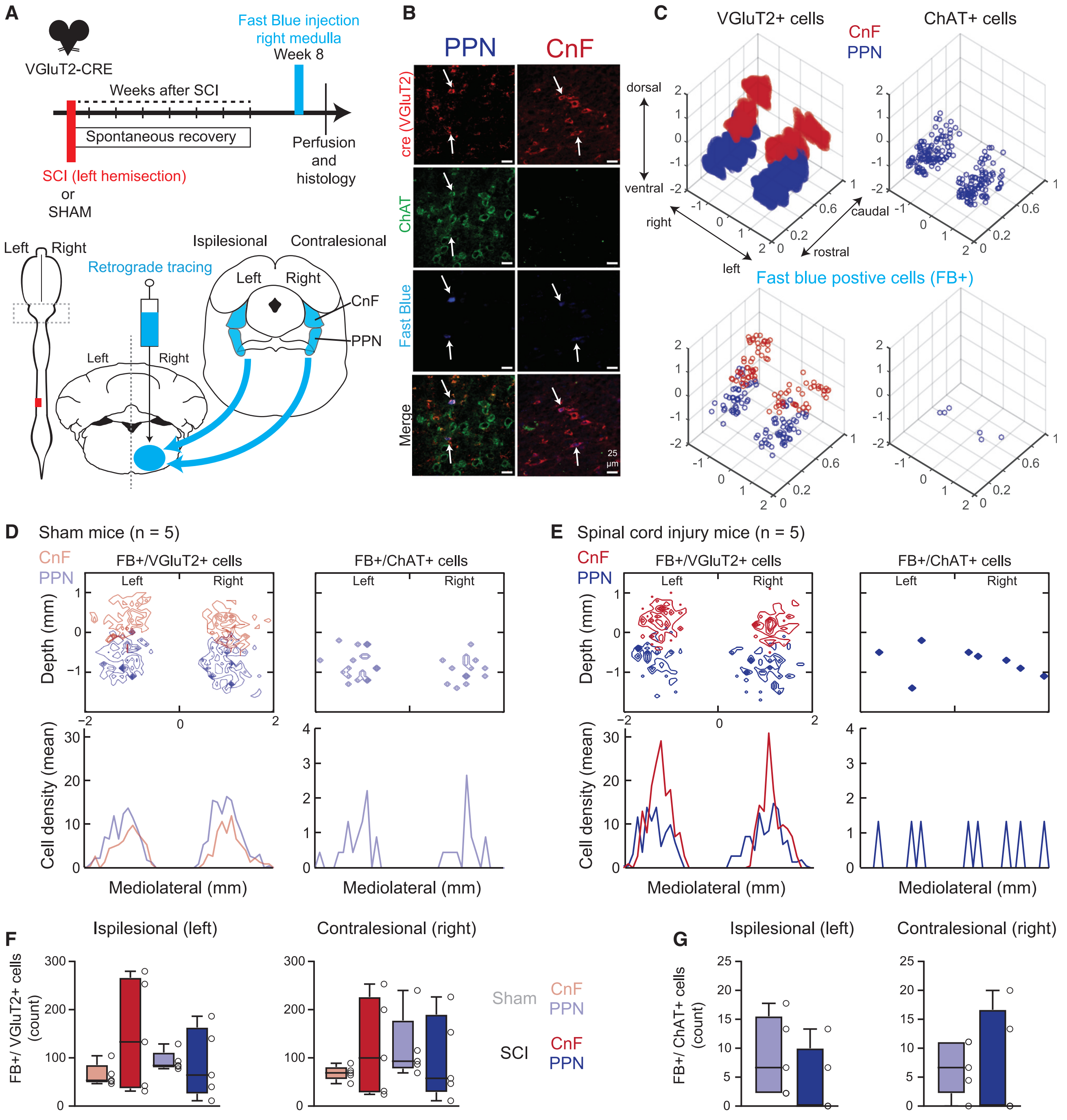

Figure 2. No anatomic change in the number of glutamatergic and cholinergic CnF and PPN neurons projecting to the motor medulla after chronic SCI

(A) Injection of a retrograde tracer, Fast Blue, in the contralesional medulla 8 weeks after SCI or after laminectomy without SCI (n = 5 SCI and 5 sham mice).

(B) Examples of immunostaining of glutamatergic (anti-Cre antibody in red) and cholinergic (anti-ChAT antibody in green) neurons with retrograde labeling (Fast Blue in blue) in the cuneiform nucleus (CnF) and pedunculopontine nucleus (PPN).

(C) Example of a spatial representation of glutamatergic neurons (VGlut2+), cholinergic neurons (ChAT+), and retrogradely labeled glutamatergic (VGlut2+/FB+) and cholinergic (ChAT+/FB+) neurons in the ipsi- and contralesional CnF and PPN of a chronic mouse. Coordinates based on the floor of the fourth ventricle, scale in mm.

(D and E) Two-dimensional mediolateral and dorsoventral distributions (top) and mediolateral projection (bottom) of retrogradely labeled glutamatergic and cholinergic mesencephalic neurons from sham (D) and SCI (E) mice.

(legend continued on next page)

(Figures 3C and 3D) but did not impair their intralimb and interlimb coordination overall (Figures 3E and S4C–S4E). In contrast, genetic ablation of glutamatergic PPN neurons had almost no significant locomotor effects, except for a decrease in the strength of coupling between the hip and the ankle (Figures 3E and S4A).

During swimming (Figures 3G–3I), however, genetic ablation of glutamatergic CnF neurons significantly decreased the angular excursion of the hip in 60% of mice, the knee in 80% of mice, and the ankle in 80% of mice, whereas ablation of glutamatergic PPN neurons significantly decreased the angular excursion of the hip in 20% of mice, the knee in 80% of mice, and the ankle in 50% of mice (Figure 3H). Genetic deletion of glutamatergic CnF neurons also decreased the swim cycle frequency and power stroke in 80% of CnF mice (Figure 3H) and swimming speed in all CnF mice (Figure 3J), whereas fewer mice were impaired upon genetic ablation of glutamatergic PPN neurons. These findings argue that glutamatergic neurons of the contralesional CnF are more important than their PPN counterparts to spontaneous locomotor recovery after SCI.

Glutamatergic CnF neurons contribute to spontaneous locomotor recovery after SCI

Having shown that glutamatergic neurons of the contralesional (right) CnF or PPN are important to spontaneous locomotor recovery after left SCI, we next assessed their functional contribution by evaluating changes in motor efficacy throughout the step cycle upon photostimulation (Figures 4A and S5 for the extent of the cre-lox recombination and location of optical fibers). The extent of lesion size and changes over time in locomotor score were similar for both CnF and PPN groups (Figure 4B).[40]

To assess changes in motor efficacy as a proxy of dynamic changes in polysynaptic connectivity between the MLR and the motoneuronal pools, the percentage of failure, excitatory, and inhibitory phase-dependent EMG responses were measured in the ipsilesional flexor and extensor muscles over a 50-ms time window upon photostimulation of 10 ms pulse duration delivered during locomotion at steady and comfortable speed before and after SCI (Figure 4C). An increase in the number of motor spikes indicated excitatory motor responses, whereas a decrease indicated inhibitory motor responses, and an absence of change indicated a failure. We quantified changes over time in the proportion of motor responses in the flexor muscle during the swing phase (Figures 4D–4F) and in the extensor during the stance phase (Figure S7) as function of the locomotor score of the ipsilesional hindlimb. There was a high failure rate in motor responses in both flexor and extensor muscles 1 week after SCI (Figures 4D and S6B), which returned eventually toward pre-injury levels over time while the animals resumed spontaneous locomotor recovery. Indeed, changes in the failure rate in both muscles correlated negatively with the locomotor score of the ipsilesional hindlimb (Figures 4D and S6B): the lower the failure rate, the higher the locomotor score, thus suggesting a transient interruption of the descending motor drive after SCI.

Regarding the flexor muscle during the swing phase (Figures 4E and 4F), changes in the proportion of excitatory motor responses correlated significantly only with changes in locomotor score upon photostimulation of glutamatergic CnF neurons (Figure 4E). In contrast, changes in the proportion of inhibitory motor responses correlated only with changes in the locomotor score upon photostimulation of glutamatergic PPN neurons (Figure 4F), suggesting that glutamatergic CnF neurons by their excitatory drive contribute more efficiently than the PPN to motor recovery of the flexor muscle during the swing phase after SCI.

Regarding the extensor muscle during the stance phase (Figures S6C and S6D), correlations between changes in the proportion of inhibitory motor responses and locomotor scores were positive upon activation of either the CnF or PPN (Figure S6D). However, changes in the proportion of excitatory responses correlated significantly and positively with changes in the locomotor score upon photostimulation of glutamatergic CnF neurons, whereas the correlation was negative upon photostimulation of glutamatergic PPN neurons (Figure S6C), thus supporting overall a higher excitatory efficiency of the CnF over the PPN in spontaneous recovery of the extensor muscle during the stance phase after SCI.

Overall, changes in the density of motor spikes in excitatory and inhibitory motor responses were positively correlated with changes in locomotor score in both flexor and extensor muscles upon photostimulation of the CnF or PPN (Figure S7). However, changes in the amplitude of motor spikes were only correlated with the locomotor score in excitatory responses of the flexor evoked upon glutamatergic CnF neurons (Figure S8). Taken together, these changes in the excitatory motor drive and behavior suggest that glutamatergic CnF neurons by their action on some hindlimb muscles might contribute more efficiently than PPN neurons to spontaneous recovery of stepping ability after SCI.

Glutamatergic neurons of the CnF promote initiation of locomotion in chronic SCI mice

Having shown that glutamatergic neurons of both the CnF and PPN are necessary and contribute to some extent to spontaneous recovery after SCI, we next investigated whether stimulation of one of these neuronal populations would be more efficient in promoting initiation of locomotion after chronic SCI (Figures 5 and S9). As illustrated by their trajectory in open field (Figures 5A), 1-s trains of photostimulation delivered above glutamatergic neurons of the right CnF generated consistent episodes of locomotion (Figures 5B and 5D: 100% of trials at 20 Hz and 84% at 50 Hz) with long-distance displacement (Figures 5A and 5D). As illustrated by body direction (Figure 5C), the first 500 ms of stimulation generated already straight locomotion (Figure 5C) at very short latency (Figure 5E). In contrast, 1-s trains of photostimulation delivered above right glutamatergic PPN neurons generated inconsistent and unreliable bouts of locomotion (Figures 5I–5K: 23% of trials at 20 Hz and 34% at 50Hz to evoke locomotion) and always occurred with a long latency after the end of the photostimulation

(F) Distribution of cell counts of glutamatergic retrogradely labeled neurons in the CnF and PPN of sham and SCI mice (Mann-Whitney tests, left CnF sham vs. SCI, $p = 0.69$; right CnF sham vs. SCI, $p = 0.69$; left PPN sham vs. SCI, $p = 0.69$; right PPN sham vs. SCI, $p = 0.42$).
(G) Distribution of cell counts of cholinergic retrogradely labeled neurons in the CnF and PPN of sham and SCI mice (Mann-Whitney tests, left PPN sham vs. SCI, $p = 0.17$; right PPN sham vs. SCI, $p = 0.90$). See also Figure S2.

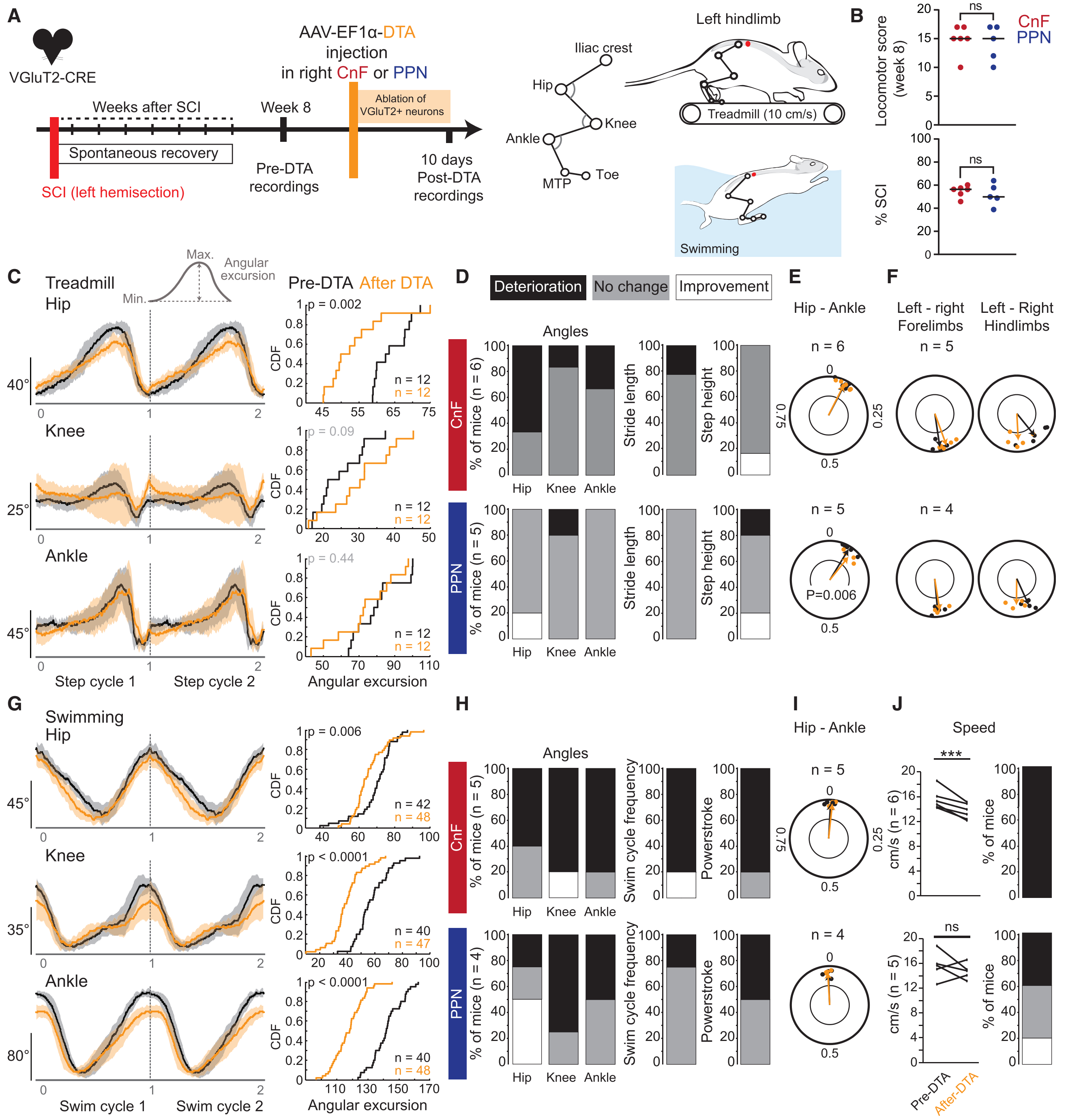

Figure 3. Genetic ablation of contralesional glutamatergic CnF or PPN neurons impairs spontaneous locomotor recovery after chronic SCI

(A) Kinematic analysis before and after genetic ablation of glutamatergic neurons of the contralesional right CnF or PPN in chronic SCI mice.

(B) Locomotor score and extent of the SCI in both experimental groups (n = 6 CnF and n = 5 PPN, unpaired t test, p = 0.53 for the locomotor score; Mann-Whitney test, p > 0.9999 for the extent of SCI).

(C) Example from one mouse of the mean and SD of hip, knee, and ankle joint angles of the ipsilesional hindlimb during treadmill locomotion before and after genetic ablation of glutamatergic CnF neurons. Cumulative distribution function (CDF) of the angular excursion of hindlimb joints (n = step cycles, Mann-Whitney test or unpaired t test according to the normality of the distribution).

(D) Percentage of mice with significant decrease (detoriation), increase (improvement), or absence of change in the angular excursion of the joints, stride length, and step height. (Each mouse was compared with its pre-DTA level using a Mann-Whitney test or unpaired t test according to the normality of the distribution).

(E) Coupling between the angle of the ipsilesional hip and ankle (each circle represents a mouse, strength of the coupling for the PPN group, paired t test, p = 0.006).

(legend continued on next page)

(Figure 5L), as previously reported.[11] In contrast to 20 Hz or 50 Hz, 5 Hz and 10 Hz trains of photostimulation of glutamatergic CnF or PPN neurons failed to evoke locomotor bouts (Figures S9A and S9B). Regarding body direction, whereas glutamatergic CnF neurons generated straight locomotion with body displacement over the first 500 ms of photostimulation (Figures 5C and 5F: 100% of trials at 20 Hz and 84% at 50 Hz), glutamatergic PPN neurons in contrast failed to initiate straight locomotion (Figures 5J and 5M: 3% of trials at 20 Hz and 5% at 50 Hz) but systematically evoked head rotation ipsilaterally to the stimulation without body displacement, as illustrated by shorter arrows (Figure 5J, 100% of trials). Although electrical stimulation in the PPN has recently been shown to induce stress,[7] we did not find any signs of discomfort upon optogenetic stimulation (Figures S9C–S9F). In summary, sole activation of glutamatergic neurons of the CnF generates straight locomotion in chronic SCI mice.

Activation of glutamatergic CnF neurons improves posture and recovery of basic and voluntary stepping in chronic SCI mice

Having shown that glutamatergic neurons of the CnF initiate episodes of locomotion in animals at rest, in contrast to the PPN (Figure 5), we next investigated how activation of these neurons can modulate posture and voluntary locomotion in a corridor. One-second train of photostimulation of glutamatergic neurons of the right CnF increased the posture (i.e., iliac crest height), step height, and step frequency, and enhanced the locomotor speed of chronic SCI mice during ongoing locomotion (Figures 6A–6C), whereas neurons of the PPN decreased locomotion and postural tone (Figures 6D and 6E). Similar observations were also reported during treadmill locomotion at a comfortable steady speed (Figure 7B for the CnF and Figure S11B for the PPN). Moreover, changes in postural tone increased linearly as a function of changes in locomotor speed: photostimulation of glutamatergic CnF neurons increased speed and posture, whereas glutamatergic PPN neurons decreased these parameters (Figure S10), thus suggesting that these two neuronal populations could modulate gait and posture.

Patients with SCI exhibit higher variability in their intralimb coordination that impedes their complete recovery.[43,44] We therefore hypothesize that by decreasing this variability, photostimulation of the glutamatergic neurons of the CnF might improve stepping ability. To further investigate that possibility, we analyzed kinematic changes of the intralimb coordination during treadmill locomotion at steady and comfortable speed of chronic SCI mice (Figure 7). As a proxy of variability, we measured the coefficient of variation of joints' angular excursion, which was especially high during the swing phase of locomotion prior to stimulation (black traces in Figure 7C). Photoactivation of the glutamatergic CnF neurons significantly decreased the variability in hindlimb joints (blue traces in Figure 7C), with the strongest changes in the hip and ankle followed by the knee (blue traces in Figures 7C, 60% of trials in the hip, 50% in the ankle, and 25% in the knee). Interestingly, this decreased variability during the swing phase co-varied according to the coefficient of variation of the hip. Indeed, when the coefficient of variation of the hip was significantly reduced, the variability of the ankle joint decreased significantly in 87% of trials and that of the knee in 47% of trials, thus supporting smoother and steadier stepping. Furthermore, analyses of EMG recordings also showed that activation of glutamatergic neurons of the CnF decreased the burst duration of flexor and extensor muscles of both ipsi- and contralesional hindlimbs (Figures 7D and 7E for the ipsilesional hindlimb and Figure S11A for the contralesional hindlimb), thus contributing to an increased locomotor speed. Stimulation also increased the burst amplitude of the ankle dorsiflexor activity (e.g., tibialis anterior), contributing to increasing the step height and toe clearance during the swing phase of locomotion. Taken together, these results suggest that stimulation of glutamatergic neurons of the CnF can efficiently modulate the spatiotemporal recruitment of muscles, smoothen and stabilize the intralimb joint coordination of the ipsilesional hindlimb, and enhance speed, thus improving overall locomotor recovery after chronic SCI.

Unloading the body weight improves locomotor functions driven by glutamatergic CnF neurons in chronic SCI mice

Although sensory feedback participates in functional locomotor recovery after SCI, unloading the body weight (even partially) is often combined with physical training to promote motor recovery in incomplete SCI patients and animal models.[45,46] Therefore, we tested whether 1-s trains of photostimulation delivered above glutamatergic neurons of either the contralesional (right) CnF or PPN can promote locomotor function while unloading the animal's weight during swimming in chronic SCI (Figures S12 and S13).

Seven weeks after SCI, activation of glutamatergic CnF neurons increased swimming speed and frequency (Figures S12A–S12D) and improved the trajectory of the hindlimb joints (Figure S12E), in addition to increasing the angular excursion of the hip in 60% of mice and of the knee and ankle in 80% of mice (Figure S12F). Furthermore, activation of the CnF significantly decreased the variability of hindlimb joints, thus improving fluidity of the movement during the power stroke and return stroke of the ipsilesional hindlimb in more than 80% of trials in the hip, knee, and ankle joints (Figure S12G). In contrast,

(F) Bilateral fore- and hindlimb coupling for all mice (each circle represents a mouse, anchored on the right forelimb and hindlimb, respectively).
(G) Example from one mouse of the mean and SD of hip, knee, and ankle joint angles of the ipsilesional hindlimb during swimming before and after genetic ablation of glutamatergic CnF neurons. CDF of the angular excursion of hindlimb joints (n = swim cycles, Mann-Whitney test or unpaired t test according to the normality of the distribution).
(H) Percentage of mice with significant decrease (detoriation), increase (improvement), or absence of change in the angular excursion, swim cycle frequency, and power stroke (each mouse was compared with its pre-DTA level using a Mann-Whitney test or unpaired t test according to the normality of the distribution).
(I) Coupling between the angle of the ipsilesional hip and ankle for all mice (each circle represents a mouse).
(J) Average speed of each mouse before and after genetic ablation (paired t tests, $p = 0.0006$ for the CnF group and $p = 0.77$ for the PPN group) and percentage of mice with significant decrease (i.e., deficit), increase (i.e., improvement), or absence of change in speed (Mann-Whitney test or unpaired t test according to the normality of the distribution). See also Figures S3 and S4.

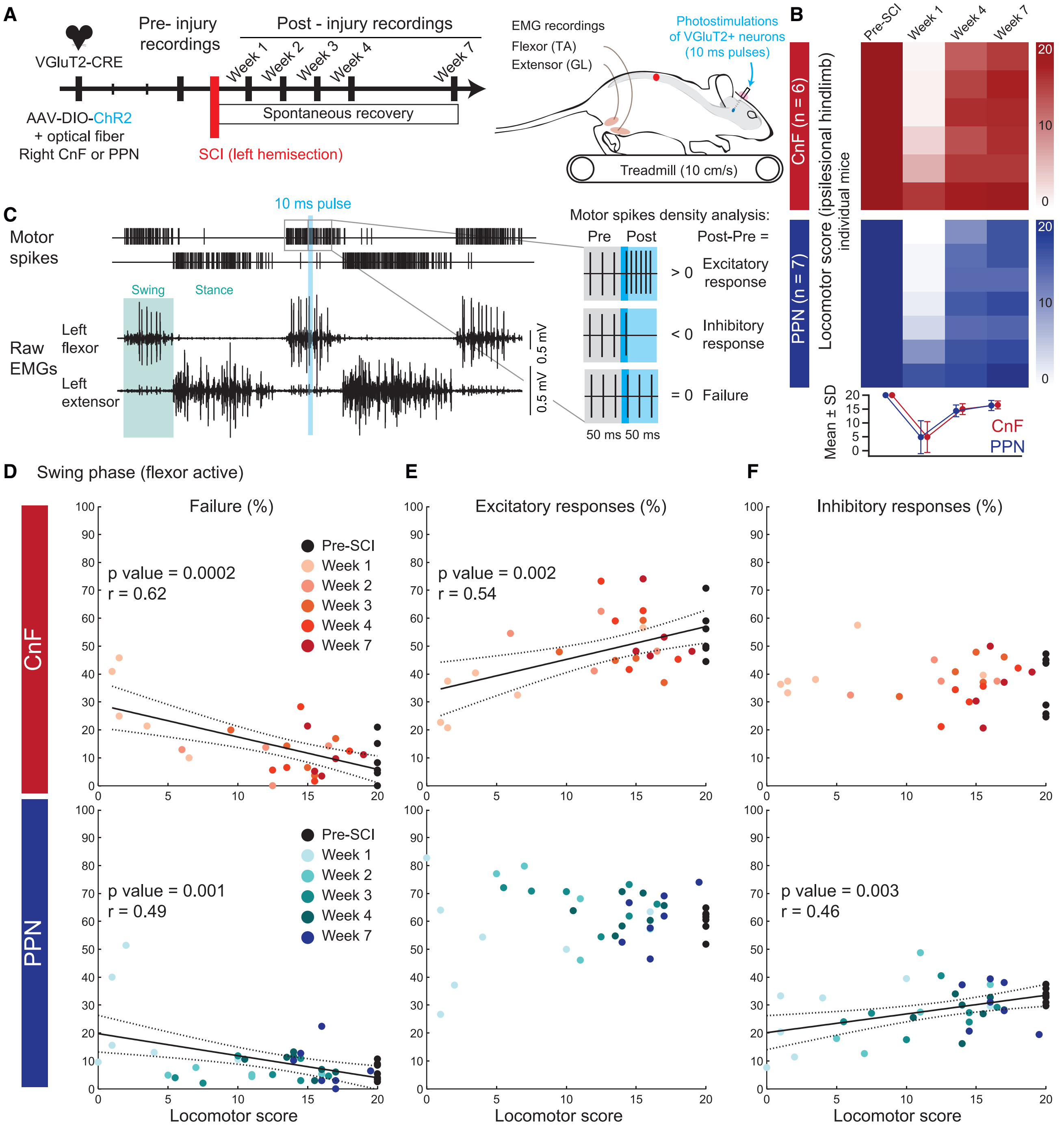

Figure 4. Glutamatergic neurons of the CnF contribute more efficiently than those of the PPN to spontaneous locomotor recovery of the ipsilesional hindlimb after SCI

(A) Combination of electromyographic (EMG) recordings with short pulses (10 ms) of photostimulation to probe changes in motor efficacy of glutamatergic CnF or PPN neurons to locomotor recovery after SCI.

(B) Color-coded matrices illustrating the locomotor score of the ipsilesional left hindlimb of mice before and after SCI. No significant difference between CnF and PPN mice (n = 6 CnF and n = 7 PPN mice, Mann-Whitney tests, p = 0.80 at week 1, p = 0.76 at week 4, and p = 0.95 at week 7).

(C) Example of background EMG activities of the ipsilesional flexor (tibialis anterior) and extensor (gastrocnemius lateralis) muscles during treadmill locomotion with a 10-ms pulse photostimulation. The difference in the density of motor spikes evoked post- vs. pre-photostimulation identified whether responses were excitatory, inhibitory, or absent (indicating a failure).

(D–F) Proportion of failure (D), excitatory (E), and inhibitory (F) motor responses evoked in the ipsilesional flexor muscle during the swing phase as a function of the locomotor score of the ipsilesional hindlimb over the course of spontaneous locomotor recovery after SCI. See also Figures S5–S8.

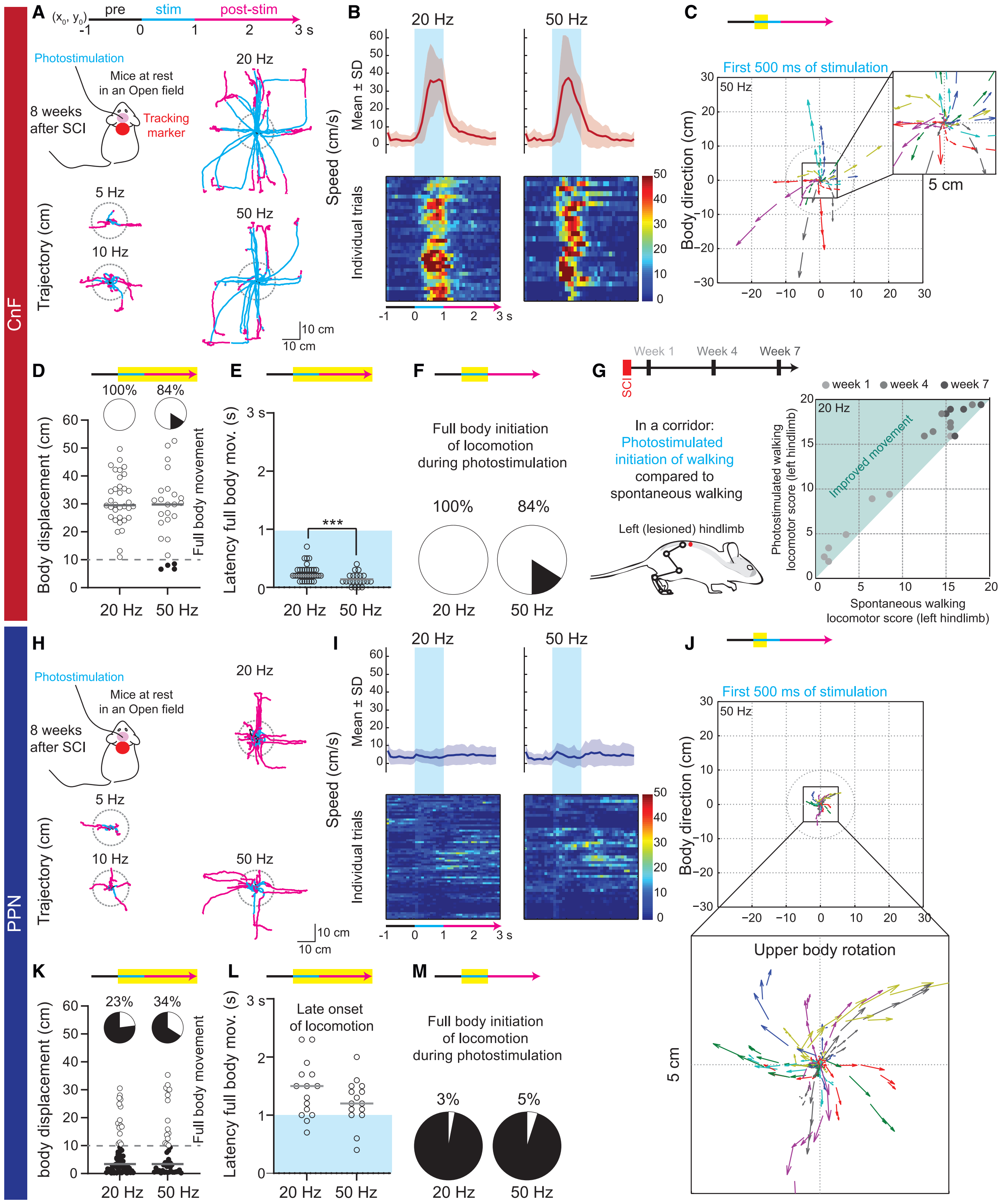

(legend on next page)

activation of glutamatergic neurons of the PPN decreased swimming speed and had very little effect on intralimb coordination of the ipsilesional hindlimb (Figure S13). Taken together, these results show that activation of glutamatergic CnF neurons promotes more efficient and smoother intralimb coordination of the ipsilesional hindlimb, thus improving functional locomotor recovery of the ipsilesional hindlimb of chronic SCI mice.

DISCUSSION

Our results reveal new concepts that fundamentally alter the understanding of the distinct contribution of MLR nuclei to functional locomotor recovery following SCI and their potential as DBS targets to promote rehabilitation in patients with chronic SCI: (1) despite an absence of neuroanatomic changes within the brainstem, (2) both glutamatergic neurons of the CnF and PPN contribute functionally differently to locomotor recovery; (3) glutamatergic neurons of the CnF are more important than those of the PPN for spontaneous locomotor recovery; (4) if glutamatergic PPN neurons contribute to the functional inhibitory drive in both flexor and extensor muscles of the ipsilesional hindlimb, only glutamatergic neurons of the CnF contribute to the functional excitatory drive of both flexor and extensor muscles; moreover (5) activation of glutamatergic neuronal populations of the CnF with 1-s trains of photostimulation at 20 Hz improve the intralimb coordination of the ipsilesional hindlimb, as well as gait and posture during basic locomotion while stepping on a treadmill and during voluntary locomotion while walking on the ground or swimming in a pool in chronic SCI. Overall, our results argue that clinical trials should consider targeting the CnF rather than the PPN, and especially glutamatergic neurons of the CnF, in patients with SCI.

The PPN is a suboptimal target for reliably generating locomotion

Using animal models, the PPN has been initially identified as an anatomic correlate of the MLR based on electrical stimulation and postmortem reconstructions.[47–49] Given that Parkinson's disease (PD) patients typically display difficulties in initiating and executing locomotor movements, with rigidity, tremor, and postural instability,[50,51] DBS of the PPN has been investigated in advanced parkinsonian patients with motor complications who are refractory to pharmacological treatments. Although PPN stimulation improves gait and postural adjustments in some parkinsonian patients,[52,53] the functional outcomes have been extremely variable across clinical studies.[54] Such variability, also reported in several animal models of PD,[55,56] has recently raised questions about the efficacy of the stimulation parameters and the anatomic correlates of the MLR.[57,58]

Although there is still no consensus regarding the most appropriate stimulation parameters, as little is known about the neural mechanisms activated by DBS in the vicinity of the PPN,[59–61] recent optogenetic studies of glutamatergic neurons of the PPN have shown functional discrepancies in animal models with locomotor initiation, deceleration, head rotation, and even anxious-like behaviors,[10,11,17,18] that could reflect the complexity of this nucleus. Indeed, in addition to the multiple pathways running through it, the PPN exhibits an extreme divergence in its presynaptic inputs and postsynaptic projections.[11,62] As shown by recent optogenetic studies, neuronal populations within the PPN can exhibit distinct functional effects according to their postsynaptic projection. If little is known about medullary-projecting PPN neurons, which will be the population most likely involved in locomotion, there is evidence that substantia nigra-projecting PPN neurons generate grooming and handling,[14] striatal-projecting PPN axons generate head rotation,[63] and spinally projecting PPN neurons induce locomotor arrest and rearing,[14] thus arguing that the PPN is a complex neurological structure with a wide range of functional motor outcomes.

Glutamatergic neurons of the CnF enable stepping ability after SCI without discomfort

Recently, electrical stimulation of the PPN has been shown to initiate bipedal locomotion of chronic SCI rats contused at the

Figure 5. Initiation of locomotion evoked by stimulation of glutamateric CnF neurons after chronic SCI

(A) Trajectories of full-body movement evoked upon long-train photostimulation of glutamatergic neurons of the CnF at different frequencies (n = 5 mice). Mice were tracked using a marker placed on their neck. Trajectories of all mice were centered at (x_0,y_0) 1 s prior to stimulation. A locomotor bout occurred if the mouse walked out of the circle (radius 10 cm) centered on the position of the body at the beginning of the stimulation.

(B) Mean and SD of locomotor speed evoked upon long trains of photostimulation of the CnF at 20 and 50 Hz. Color-coded matrices representing individual trials (100-ms bins).

(C) Vectors represent the upper body direction and the distance traveled (vector length) during the first 500 ms of photostimulation of the CnF at 50 Hz (highlighted in yellow on the timeline).

(D) Body displacement evoked by photostimulation of the CnF at 20 (34 trials) and 50 Hz (25 trials). A 10-cm displacement was considered as a full-body movement (i.e., an initiation of locomotion). All displacements occurring within the 3 s from the start of the stimulation were considered (highlighted on the timeline).

(E) Latency to initiate full-body movement (>10 cm) upon stimulation of the CnF at 20 (34 trials) and 50 Hz (21 trials; Mann-Whitney test, p = 0.001).

(F) Percentage of trials in which initiation of locomotion started within the first second of photostimulation of the CnF at 20 and 50 Hz.

(G) Locomotor score of the left ipsilesional hindlimb during spontaneous vs. stimulated locomotion of the CnF at 20Hz over time after SCI (n = 7 mice).

(H) Trajectories of full-body movement evoked upon photostimulation of glutamatergic neurons of the PPN (n = 7 mice).

(I) Mean and SD of locomotor speed evoked upon long trains of photostimulation of the PPN at 20 and 50 Hz. Color-coded matrices representing individual trials (100 ms bins).

(J) Vectors illustrate consistent upper body rotation ipsilateral to the stimulation site within a 5-cm radius; 50-Hz photostimulation of the PPN evoked consistent rapid motor movements with little displacement, considered as a postural rotation.

(K) Body displacement evoked by photostimulation of the PPN at 20 (67 trials) and 50 Hz (45 trials).

(L) Latency to initiate full-body movement (>10 cm) started after the end of the PPN photostimulation (20 Hz, 15 trials; 50 Hz, 15 trials).

(M) Percentage of trials in which initiation of locomotion started within the first second of photostimulation of the PPN at 20 and 50 Hz. See also Figure S9.

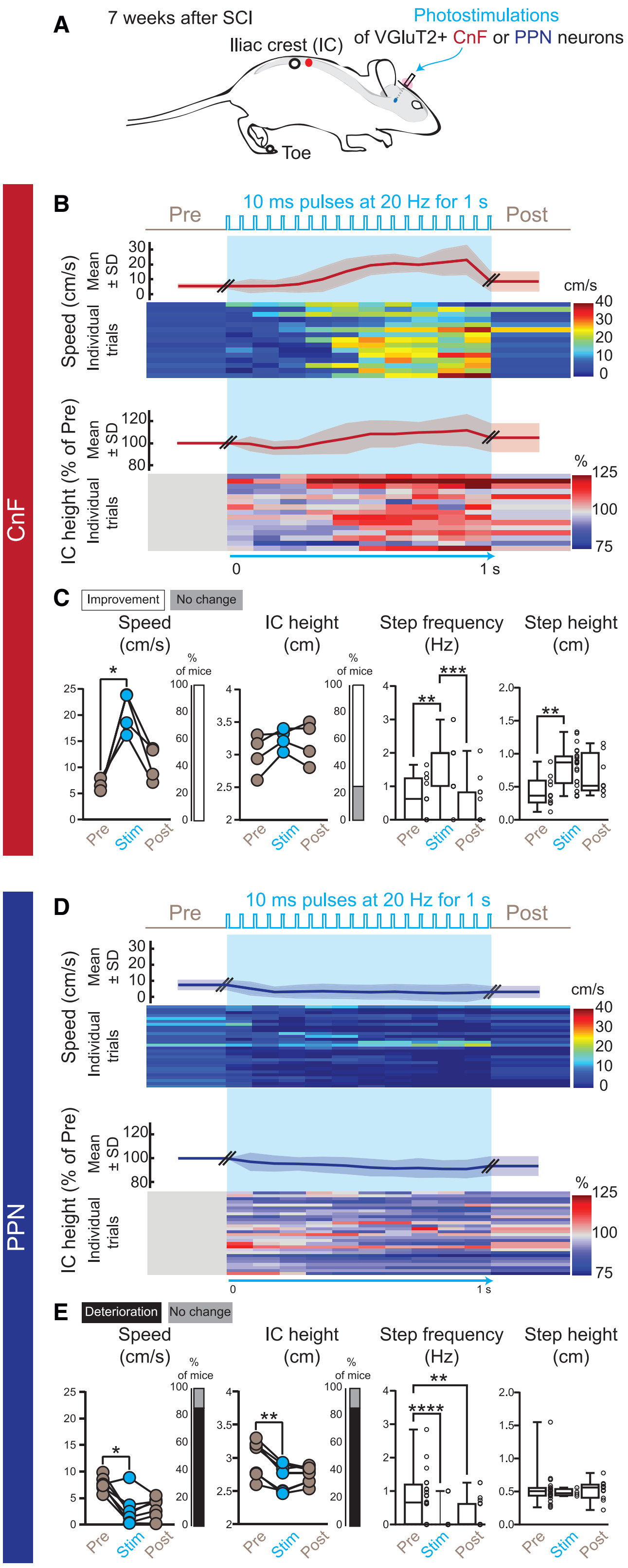

Figure 6. Functional improvement during overground locomotion upon long trains of photostimulation of glutamatergic CnF neurons after chronic SCI

(A) Trains of photostimulation (10-ms pulses at 20 Hz for 1 s) delivered in glutamatergic neurons of the CnF (n = 4) or PPN (n = 7) after chronic SCI (7 weeks after SCI).

(B) Time course of locomotor speed (mean and SD) and individual trials (below, 3–4 trials per mouse, 100-ms bins). Time course of the iliac crest height (mean and SD) and individual trials normalized on data prior to photostimulation (below).

(C) Mean of locomotor speed of individual mouse evoked upon photostimulation of glutamatergic CnF neurons (500 ms pre/stim/post periods, last 500 ms of the stimulation episode; Friedman test [p = 0.04] with Dunn's multiple comparison test, *p < 0.05). Percentage of mice exhibiting a significant increase (i.e., improvement) in speed upon photostimulation in comparison to the pre-stimulation period (Mann-Whitney test or unpaired t test according to the normality of the distribution). Mean height of the iliac crest of each mouse. Percentage of mice exhibiting a significant increase (i.e., improvement) in the height of the iliac crest upon photostimulation (Mann-Whitney test or unpaired t test according to the normality of the distribution). Distribution of the step frequency (15 trials combined, Kruskal-Wallis test [p = 0.0003] with Dunn's multiple comparisons test, ***p < 0.001, **p < 0.01). Step height of all step cycles (pre n = 11; stim n = 25; post n = 9; Kruskal-Wallis test [p = 0.009] with Dunn's multiple comparisons test, **p < 0.01).

(D) Same as (B).

(E) Mean of locomotor speed of individual mouse evoked upon photostimulation of glutamatergic PPN neurons (Friedman test [p = 0.02] with Dunn's multiple comparison test, *p < 0.05). Percentage of mice exhibiting a decrease (i.e., deficit) in speed upon photostimulation of the PPN (Mann-Whitney test or unpaired t test according to the normality of the distribution). Mean of the height of the iliac crest (Friedman test [p = 0.008] with Dunn's multiple comparison test, **p < 0.01). Percentage of mice exhibiting a decrease (i.e., deficit) in the height of the iliac crest upon photostimulation of the PPN (Mann-Whitney test or unpaired t test according to the normality of the distribution). Distribution of the step frequency (28 trials combined; Kruskal-Wallis test [p = 0.0001] with Dunn's multiple comparison test, **p < 0.01, ****p < 0.0001). Step height of residual steps occurring upon photostimulation of the glutamatergic PPN (pre n = 27; stim n = 4; post n = 11; Kruskal-Wallis test, p = 0.61). See also Figure S10.

thoracic level.[7] However, electrical PPN stimulation also appears to induce pain as reported by a grimace test in these chronic SCI animals. However, using optogenetic tools in the mouse, we found that photoactivation of glutamatergic neurons of the PPN did not induce any signs of discomfort in chronic SCI mice. Although we cannot exclude some discrepancies between both animal species and SCI models, electrical stimulation is not as specific as optogenetic activation in recruiting different neuronal populations, axons of passage, and unwanted neural structures in the vicinity of the electrode. By their caudal location to the PPN, electrical stimulation of the PPN might have also recruited the Kölliker/parabrachial nuclei involved in grimaces and aversive responses during pain.[64,65] In contrast, optogenetic stimulation of glutamatergic neurons, especially in the CnF, resulted in almost no or very little apparent discomfort in chronic SCI mice.

Electrical stimulation of the CnF has also been recently shown to promote quadrupedal locomotion of chronic rats with a severe thoracic section sparing only the ventromedial funiculus.[8] Using a less severe thoracic SCI, we also found that optogenetic photostimulation of glutamatergic neurons of the CnF with 1-s trains of 20 Hz increased postural tone and initiated locomotion in chronic SCI mice, with functionally better stepping ability and smoothness

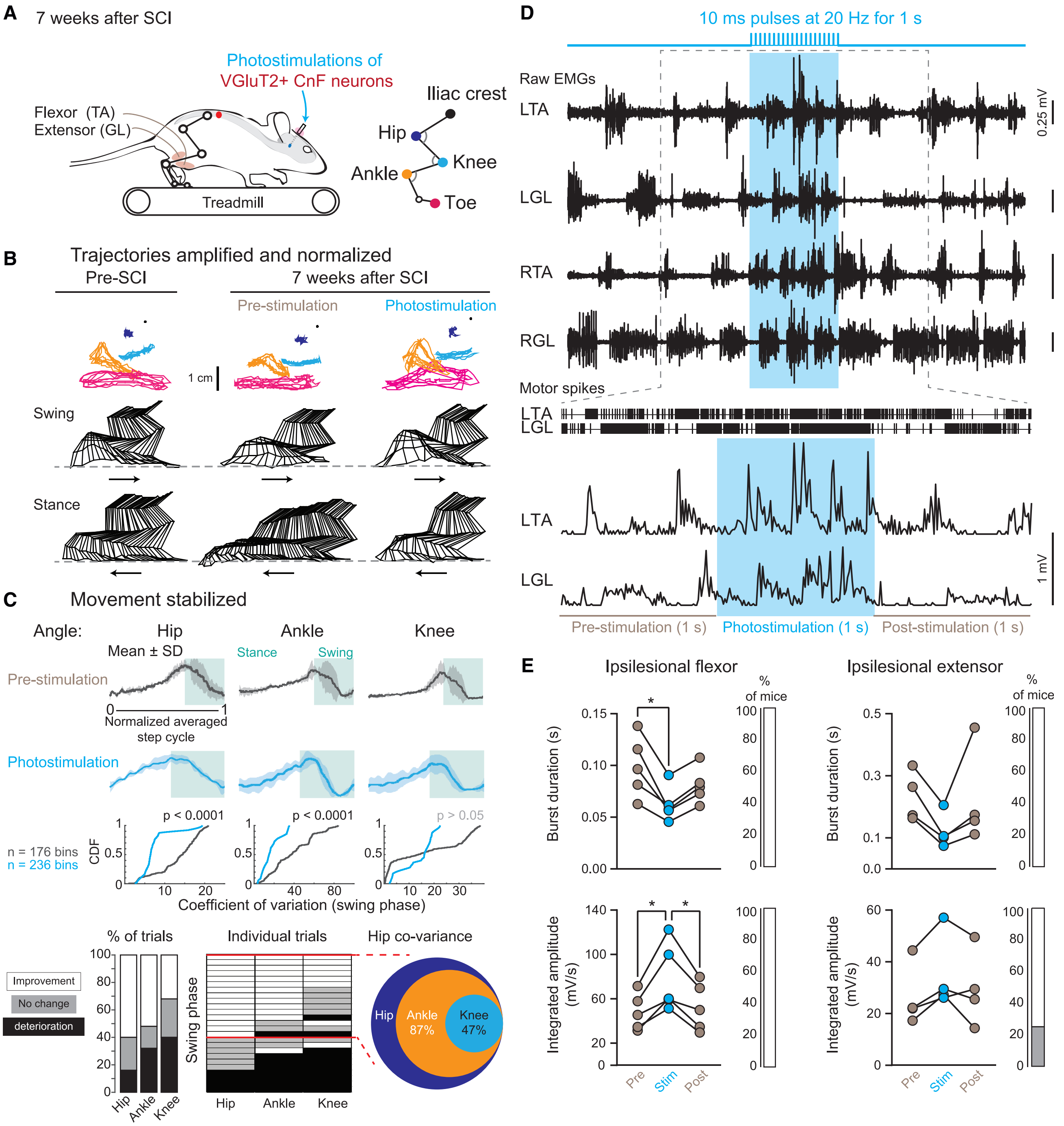

Figure 7. Functional improvement of intralimb coordination during treadmill locomotion upon long trains of photostimulation of glutamatergic CnF neurons after chronic SCI

(A) Kinematic and EMG recordings of the ipsilesional hindlimb evoked upon long trains of photostimulation (10 ms at 20 Hz for 1 s) of glutamatergic CnF neurons during treadmill locomotion 7 weeks after SCI (n = 5 mice).

(B) Cyclic trajectories of hindlimb joints (anchored on the iliac crest) during locomotion before and after chronic SCI. Stick diagrams illustrate the swing and stance phases (arrows show the direction of movement).

(C) Mean and SD of the hip, knee, and ankle joint angles during a normalized step cycle (512 bins). Green areas indicate the swing phase of the step cycle. Cumulative distribution function (CDF) of coefficients of variation of hindlimb joints during the swing phase (n = swing phase bins, pre vs. stim, Mann-Whitney test or unpaired t test according to the normality of the distribution). Percentage of trials in which photostimulation induced a decrease (improvement) or increase (deterioration) in coefficients of variation of hip, knee, and ankle joint angles during the swing phase (left). Individual trials (n = 25, five trials per mouse) were aligned

(legend continued on next page)

in locomotion than during spontaneous episode of locomotion. Although stimulation of glutamatergic neurons of the PPN usually failed to evoke locomotion with our standard stimulation protocol at 20 Hz, increasing the stimulation frequency up to 50 Hz induced systematic upper body rotation ipsilaterally to the stimulation followed by very slow locomotor movements. These locomotor bouts, however, were not reliable and occurred only at very long latency after the end of the stimulation before and after SCI, as previously shown in intact mice.[11] Interestingly, using trains of 40 Hz for 10 s, glutamatergic neurons of the PPN have been recently shown to initiate locomotion at long latency in intact and akinetic mouse models following a pharmacological block of dopaminergic transmission.[16] Similar results have also been reported upon photostimulation at 20 Hz for 10 s of glutamatergic neurons of the CnF in intact mice and after severe acute dopaminergic depletion.[12,13] Although we cannot exclude the possibility that long photostimulation for 10 s could also generate locomotion after SCI, stimulations of 20 Hz for 1 s in the CnF were sufficient to initiate locomotion and improve inter- and intralimb coordination after chronic SCI. Overall, our findings suggest that the CnF and especially glutamatergic neurons of the CnF are a better target than the PPN for promoting functional recovery of locomotor initiation in chronic SCI.

Glutamatergic CnF neurons are necessary and sufficient to improve functional recovery during locomotion

Genetic anterograde and retrograde tracing studies have shown a direct connection between glutamatergic neurons of both the CnF and PPN nuclei and the medulla.[11,14] Pharmacological, lesion, cooling, and genetic deletion studies have previously shown that reticulospinal pathways of the medullary reticular formation relay supraspinal MLR inputs to the spinal locomotor circuit.[29–31,35] Not surprisingly, reticulospinal pathways appear to contribute to functional locomotor recovery following SCI,[9,66–68] presumably by relaying MLR and/or cortical inputs. Although anatomic reorganization has been reported between medullary-projecting midbrain neurons following cervical SCI[9] and axonal midbrain sprouting in the medulla after thoracic SCI,[8] we found no anatomic changes in medullary-projecting CnF or PPN glutamatergic neurons after thoracic SCI. This discrepancy in retrogradely labeled neurons could reflect some changes in other genetically unidentified neurons, or more likely differences in SCI models. However, despite the absence of anatomic changes, we found functional behavioral and motor improvements when stimulating glutamatergic CnF neurons. Indeed, genetic deletion of glutamatergic neurons of the CnF or PPN impaired spontaneous locomotor recovery during walking and swimming, but deficits were stronger upon genetic ablation of CnF neurons than those of the PPN. Similarly, motor efficacy measurements also revealed that excitatory motor responses evoked in ipsilesional flexor and extensor muscles by glutamatergic neurons of the CnF correlated robustly with changes in locomotor recovery. This was not the case with the PPN, suggesting that glutamatergic neurons of the CnF contribute more efficiently than those of the PPN to spontaneous recovery of locomotor functions following SCI.

Glutamatergic CnF neurons promote functional locomotor recovery in chronic SCI mice

As previously shown before SCI,[10] activation of glutamatergic CnF neurons increased postural tone as well as locomotor pattern and rhythm in chronic SCI mice during treadmill locomotion, as well as during voluntary locomotion while walking in a corridor or swimming in a pool, whereas activation of glutamatergic PPN neurons usually slowed locomotor rhythm, eventually inducing locomotor arrests. Interestingly, similar decelerations and stops have also been recently reported upon photostimulation of PPN neurons projecting to the spinal cord in intact animals.[14]

Although far from complete, there is growing understanding of the neural brainstem networks underlying locomotion.[69,70] Recent optogenetic studies have shown that photostimulation of glutamatergic neurons of the LPGi initiates locomotion and increases locomotor rhythm,[35] presumably by relaying glutamatergic inputs of the CnF. In contrast, photoactivation of glutamatergic or glutamatergic V2a (Lhx3/Chx10) expressing neurons of the gigantocellular reticular nucleus (GI) reset locomotor rhythm and induce locomotor arrests,[33,34,71,72] presumably by relaying glutamatergic inputs of the PPN. Further studies are needed to genetically dissect the neural mechanisms of these medullary nuclei and their reticulospinal projections to motor recovery after SCI. Overall, our results support the hypothesis that glutamatergic CnF neurons are more efficient than those of the PPN in improving stepping ability of the ipsilesional hindlimb, in addition to enhancing gait and posture, in chronic SCI mice during stereotyped and voluntary locomotion.

Limitations of the study

With a current clinical trial assessing MLR DBS in patients with incomplete SCI,[21,73] our findings reveal that the CnF offers a better and more reliable neurological target in comparison with the PPN for promoting recovery of motor and locomotor functions, strengthening the importance of evaluating the role of the CnF and especially glutamatergic neurons of the CnF in patients suffering from SCI. Our results also highlight the importance of continuing genetic dissection of functional microcircuits within the PPN in biomedical research. Although optogenetic and optical technologies are still in their infancy regarding first-in-human clinical trials targeting more accessible neural structures to

according to the evolution of coefficients of variation of the hip angle (middle). Pie chart showing that ankle and knee stabilization occurred mainly when the hip was stabilized (right).

(D) Raw EMG recordings (top), raster of motor spikes of the LTA and LGL (middle), and rectified EMG traces of the LTA and LGL at higher magnification (below).

(E) Burst duration and integrated amplitude of background EMG bursts in the ipsilesional flexor (n = 5) and extensor (n = 4; burst duration of the ipsilesional flexor Friedman test [p = 0.009] with Dunn's multiple comparison test, *p < 0.05; integrated amplitude of the ipsilesional flexor, repeated measures one-way ANOVA [p = 0.008] with Tukey's comparison test, *p < 0.05). Percentage of mice showing a significant improvement (in white, decrease in burst duration, increase in burst amplitude) or no change (gray) upon photostimulation period compared with pre period (pre vs. stim periods, Mann-Whitney test or unpaired t test according to the normality of the distribution). For abbreviations, see Figure 1 legend. See also Figures S11–S13.

restore sensory loss,[74,75] advances are needed for control of deep brain neuronal populations to promote recovery of gait and posture in humans suffering from SCI or neurodegenerative diseases, such as PD or amyotrophic lateral sclerosis. In summary, our current findings in an animal model of SCI suggest that DBS of the CnF or optogenetic activation of glutamatergic CnF neurons should be further investigated in chronic SCI patients.

STAR★METHODS

Detailed methods are provided in the online version of this paper and include the following:

- KEY RESOURCES TABLE
- RESOURCE AVAILABILITY
  - Lead contact
  - Materials availability
  - Data and code availability
- EXPERIMENTAL MODEL AND SUBJECT DETAILS
- METHOD DETAILS
  - AAV injections and optical fiber implantations
  - Spinal cord injury
  - Retrograde tracing
  - Locomotor tests
  - Genetic ablation of glutamatergic neurons
  - Optogenetic and electrophysiological recordings
  - Kinematic analysis
  - EMG analysis
  - Perfusion
  - Immunochemistry
  - Stereology
- QUANTIFICATION AND STATISTICAL ANALYSIS

SUPPLEMENTAL INFORMATION

Supplemental information can be found online at https://doi.org/10.1016/j.xcrm.2023.100946.

ACKNOWLEDGMENTS

We thank L. Bihoues, Q. Bruxelles, A. Caussaint, G. Clain, G. Gélinas, H. Godet, S. Harvey, N. Karimi, and V. Langevin for their participation in animal handling, data acquisition, and analysis; Pr. J.-F. Lalonde, C. Panter, and P. Drapeau for technical assistance; and M.-C. Richer and S. Bernard for supporting our animal care. This work was supported by Wings for Life and Canadian Institutes of Health Research grants to F.B. F.B. was supported by a salary award from Fonds de Recherche Québec Santé (FRQS) and M.L. was supported by a Wings for Life postdoctoral fellowship.

AUTHOR CONTRIBUTIONS

F.B. conceived and designed the research; M.R., D.L.Z., N.J., and M.L. performed research and analyzed data; M.R. and F.B. drafted the manuscript; M.R., M.L., and F.B. edited and revised the manuscript; and all authors approved the final version of the manuscript.

DECLARATION OF INTERESTS

The authors declare no competing interests.

INCLUSION AND DIVERSITY

We support inclusive, diverse, and equitable conduct of research.

Received: August 23, 2022
Revised: December 9, 2022
Accepted: January 23, 2023
Published: February 21, 2023

REFERENCES

1. Bonizzato, M., Pidpruzhnykova, G., DiGiovanna, J., Shkorbatova, P., Pavlova, N., Micera, S., and Courtine, G. (2018). Brain-controlled modulation of spinal circuits improves recovery from spinal cord injury. Nat. Commun. *9*, 3015.
2. Capogrosso, M., Milekovic, T., Borton, D., Wagner, F., Moraud, E.M., Mignardot, J.B., Buse, N., Gandar, J., Barraud, Q., Xing, D., et al. (2016). A brain–spine interface alleviating gait deficits after spinal cord injury in primates. Nature *539*, 284–288.
3. Wagner, F.B., Mignardot, J.B., Le Goff-Mignardot, C.G., Demesmaeker, R., Komi, S., Capogrosso, M., Rowald, A., Seáñez, I., Caban, M., Pirondini, E., et al. (2018). Targeted neurotechnology restores walking in humans with spinal cord injury. Nature *563*, 65–71.
4. Wenger, N., Moraud, E.M., Gandar, J., Musienko, P., Capogrosso, M., Baud, L., Le Goff, C.G., Barraud, Q., Pavlova, N., Dominici, N., et al. (2016). Spatiotemporal neuromodulation therapies engaging muscle synergies improve motor control after spinal cord injury. Nat. Med. *22*, 138–145.
5. Bonizzato, M., and Martinez, M. (2021). An intracortical neuroprosthesis immediately alleviates walking deficits and improves recovery of leg control after spinal cord injury. Sci. Transl. Med. *13*, eabb4422.
6. Bachmann, L.C., Matis, A., Lindau, N.T., Felder, P., Gullo, M., and Schwab, M.E. (2013). Deep brain stimulation of the midbrain locomotor region improves paretic hindlimb function after spinal cord injury in rats. Sci. Transl. Med. *5*, 208ra146.
7. Bonizzato, M., James, N.D., Pidpruzhnykova, G., Pavlova, N., Shkorbatova, P., Baud, L., Martinez-Gonzalez, C., Squair, J.W., DiGiovanna, J., Barraud, Q., et al. (2021). Multi-pronged neuromodulation intervention engages the residual motor circuitry to facilitate walking in a rat model of spinal cord injury. Nat. Commun. *12*, 1925.
8. Hofer, A.S., Scheuber, M.I., Sartori, A.M., Good, N., Stalder, S.A., Hammer, N., Fricke, K., Schalbetter, S.M., Engmann, A.K., Weber, R.Z., et al. (2022). Stimulation of the cuneiform nucleus enables training and boosts recovery after spinal cord injury. Brain *145*, 3681–3697.
9. Zörner, B., Bachmann, L.C., Filli, L., Kapitza, S., Gullo, M., Bolliger, M., Starkey, M.L., Röthlisberger, M., Gonzenbach, R.R., and Schwab, M.E. (2014). Chasing central nervous system plasticity: the brainstem's contribution to locomotor recovery in rats with spinal cord injury. Brain *137*, 1716–1732.
10. Josset, N., Roussel, M., Lemieux, M., Lafrance-Zoubga, D., Rastqar, A., and Bretzner, F. (2018). Distinct contributions of mesencephalic locomotor region nuclei to locomotor control in the freely behaving mouse. Curr. Biol. *28*, 884–901.e3.
11. Caggiano, V., Leiras, R., Goñi-Erro, H., Masini, D., Bellardita, C., Bouvier, J., Caldeira, V., Fisone, G., and Kiehn, O. (2018). Midbrain circuits that set locomotor speed and gait selection. Nature *553*, 455–460.
12. van der Zouwen, C.I., Boutin, J., Fougère, M., Flaive, A., Vivancos, M., Santuz, A., Akay, T., Sarret, P., and Ryczko, D. (2021). Freely behaving mice can brake and turn during optogenetic stimulation of the mesencephalic locomotor region. Front. Neural Circuits *15*, 639900.
13. Fougère, M., van der Zouwen, C.I., Boutin, J., Neszvecsko, K., Sarret, P., and Ryczko, D. (2021). Optogenetic stimulation of glutamatergic neurons in the cuneiform nucleus controls locomotion in a mouse model of Parkinson's disease. Proc. Natl. Acad. Sci. USA *118*. e2110934118.

14. Ferreira-Pinto, M.J., Kanodia, H., Falasconi, A., Sigrist, M., Esposito, M.S., and Arber, S. (2021). Functional diversity for body actions in the mesencephalic locomotor region. Cell *184*, 4564–4578.e18.
15. Dautan, D., Kovács, A., Bayasgalan, T., Diaz-Acevedo, M.A., Pal, B., and Mena-Segovia, J. (2020). Modulation of motor behavior by the mesencephalic locomotor region. Cell Rep. *36*, 109594.
16. Masini, D., and Kiehn, O. (2022). Targeted activation of midbrain neurons restores locomotor function in mouse models of parkinsonism. Nat. Commun. *13*, 504.
17. Carvalho, M.M., Tanke, N., Kropff, E., Witter, M.P., Moser, M.-B., and Moser, E.I. (2020). A brainstem locomotor circuit drives the activity of speed cells in the medial entorhinal cortex. Cell Rep. *32*, 108123.
18. Kroeger, D., Ferrari, L.L., Petit, G., Mahoney, C.E., Fuller, P.M., Arrigoni, E., and Scammell, T.E. (2017). Cholinergic, glutamatergic, and GABAergic neurons of the pedunculopontine tegmental nucleus have distinct effects on sleep/wake behavior in mice. J. Neurosci. *37*, 1352–1366.
19. Lee, A.M., Hoy, J.L., Bonci, A., Wilbrecht, L., Stryker, M.P., and Niell, C.M. (2014). Identification of a brainstem circuit regulating visual cortical state in parallel with locomotion. Neuron *83*, 455–466.
20. Roseberry, T.K., Lee, A.M., Lalive, A.L., Wilbrecht, L., Bonci, A., and Kreitzer, A.C. (2016). Cell-type-specific control of brainstem locomotor circuits by basal ganglia. Cell *164*, 526–537.
21. Stieglitz, L.H., Hofer, A.-S., Bolliger, M., Oertel, M.F., Filli, L., Willi, R., Cathomen, A., Meyer, C., Schubert, M., Hubli, M., et al. (2021). Deep brain stimulation for locomotion in incomplete human spinal cord injury (DBS-SCI): protocol of a prospective one-armed multi-centre study. BMJ Open *11*, e047670.
22. Barrière, G., Leblond, H., Provencher, J., and Rossignol, S. (2008). Prominent role of the spinal central pattern generator in the recovery of locomotion after partial spinal cord injuries. J. Neurosci. *28*, 3976–3987.
23. Barrière, G., Frigon, A., Leblond, H., Provencher, J., and Rossignol, S. (2010). Dual spinal lesion paradigm in the cat: evolution of the kinematic locomotor pattern. J. Neurophysiol. *104*, 1119–1133.
24. Martinez, M., Delivet-Mongrain, H., Leblond, H., and Rossignol, S. (2012). Effect of locomotor training in completely spinalized cats previously submitted to a spinal hemisection. J. Neurosci. *32*, 10961–10970.
25. Martinez, M., Delivet-Mongrain, H., Leblond, H., and Rossignol, S. (2012). Incomplete spinal cord injury promotes durable functional changes within the spinal locomotor circuitry. J. Neurophysiol. *108*, 124–134.
26. Courtine, G., Song, B., Roy, R.R., Zhong, H., Herrmann, J.E., Ao, Y., Qi, J., Edgerton, V.R., and Sofroniew, M.V. (2008). Recovery of supraspinal control of stepping via indirect propriospinal relay connections after spinal cord injury. Nat. Med. *14*, 69–74.
27. Helgren, M.E., and Goldberger, M.E. (1993). The recovery of postural reflexes and locomotion following low thoracic hemisection in adult cats involves compensation by undamaged primary afferent pathways. Exp. Neurol. *123*, 17–34.
28. Kuhtz-Buschbeck, J.P., Boczek-Funcke, A., Mautes, A., Nacimiento, W., and Weinhardt, C. (1996). Recovery of locomotion after spinal cord hemisection: an X-ray study of the cat hindlimb. Exp. Neurol. *137*, 212–224.
29. Noga, B.R., Kriellaars, D.J., and Jordan, L.M. (1991). The effect of selective brainstem or spinal cord lesions on treadmill locomotion evoked by stimulation of the mesencephalic or pontomedullary locomotor regions. J. Neurosci. *11*, 1691–1700.
30. Noga, B.R., Kriellaars, D.J., Brownstone, R.M., and Jordan, L.M. (2003). Mechanism for activation of locomotor centers in the spinal cord by stimulation of the mesencephalic locomotor region. J. Neurophysiol. *90*, 1464–1478.
31. Shefchyk, S.J., Jell, R.M., and Jordan, L.M. (1984). Reversible cooling of the brainstem reveals areas required for mesencephalic locomotor region evoked treadmill locomotion. Exp. Brain Res. *56*, 257–262.
32. Bretzner, F., and Brownstone, R.M. (2013). Lhx3-Chx10 reticulospinal neurons in locomotor circuits. J. Neurosci. *33*, 14681–14692.
33. Lemieux, M., and Bretzner, F. (2019). Glutamatergic neurons of the gigantocellular reticular nucleus shape locomotor pattern and rhythm in the freely behaving mouse. PLoS Biol. *17*, e2003880.
34. Bouvier, J., Caggiano, V., Leiras, R., Caldeira, V., Bellardita, C., Balueva, K., Fuchs, A., and Kiehn, O. (2015). Descending command neurons in the brainstem that halt locomotion. Cell *163*, 1191–1203.
35. Capelli, P., Pivetta, C., Soledad Esposito, M., and Arber, S. (2017). Locomotor speed control circuits in the caudal brainstem. Nature *551*, 373–377.
36. Lavoie, B., and Parent, A. (1994). Pedunculopontine nucleus in the squirrel monkey: distribution of cholinergic and monoaminergic neurons in the mesopontine tegmentum with evidence for the presence of glutamate in cholinergic neurons. J. Comp. Neurol. *344*, 190–209.
37. Luquin, E., Huerta, I., Aymerich, M.S., and Mengual, E. (2018). Stereological estimates of glutamatergic, GABAergic, and cholinergic neurons in the pedunculopontine and laterodorsal tegmental nuclei in the rat. Front. Neuroanat. *12*, 34.
38. Wang, H.-L., and Morales, M. (2009). Pedunculopontine and laterodorsal tegmental nuclei contain distinct populations of cholinergic, glutamatergic and GABAergic neurons in the rat. Eur. J. Neurosci. *29*, 340–358.
39. Steinkellner, T., Yoo, J.H., and Hnasko, T.S. (2019). Differential expression of VGLUT2 in mouse mesopontine cholinergic neurons. eNeuro *6*. ENEURO.0161-19.2019.
40. Brown, A.R., and Martinez, M. (2018). Ipsilesional motor cortex plasticity participates in spontaneous hindlimb recovery after lateral hemisection of the thoracic spinal cord in the rat. J. Neurosci. *38*, 9977–9988.
41. Foster, E., Wildner, H., Tudeau, L., Haueter, S., Ralvenius, W.T., Jegen, M., Johannssen, H., Hösli, L., Haenraets, K., Ghanem, A., et al. (2015). Targeted ablation, silencing, and activation establish glycinergic dorsal horn neurons as key components of a spinal gate for pain and itch. Neuron *85*, 1289–1304.
42. Kaur, S., Wang, J.L., Ferrari, L., Thankachan, S., Kroeger, D., Venner, A., Lazarus, M., Wellman, A., Arrigoni, E., Fuller, P.M., and Saper, C.B. (2017). A genetically defined circuit for arousal from sleep during hypercapnia. Neuron *96*, 1153–1167.e5.
43. Easthope, C.S., Traini, L.R., Awai, L., Franz, M., Rauter, G., Curt, A., and Bolliger, M. (2018). Overground walking patterns after chronic incomplete spinal cord injury show distinct response patterns to unloading. J. Neuroeng. Rehabil. *15*, 102.
44. Sohn, W.J., Tan, A.Q., Hayes, H.B., Pochiraju, S., Deffeyes, J., and Trumbower, R.D. (2018). Variability of leg kinematics during overground walking in persons with chronic incomplete spinal cord injury. J. Neurotrauma *35*, 2519–2529.
45. Alcobendas-Maestro, M., Esclarín-Ruz, A., Casado-López, R.M., Muñoz-González, A., Pérez-Mateos, G., González-Valdizán, E., and Martín, J.L.R. (2012). Lokomat robotic-assisted versus overground training within 3 to 6 months of incomplete spinal cord lesion: randomized controlled trial. Neurorehabil. Neural Repair *26*, 1058–1063.
46. de Leon, R.D., and Dy, C.J. (2017). What did we learn from the animal studies of body weight–supported treadmill training and where do we go from here. J. Neurotrauma *34*, 1744–1750.
47. Garcia-Rill, E., Skinner, R.D., and Fitzgerald, J.A. (1985). Chemical activation of the mesencephalic locomotor region. Brain Res. *330*, 43–54.
48. Skinner, R.D., Kinjo, N., Henderson, V., and Garcia-Rill, E. (1990). Locomotor projections from the pedunculopontine nucleus to the spinal cord. Neuroreport *1*, 183–186.
49. Garcia-Rill, E., and Skinner, R.D. (1987). The mesencephalic locomotor region. I. Activation of a medullary projection site. Brain Res. *411*, 1–12.
50. Morris, M.E., Huxham, F., McGinley, J., Dodd, K., and Iansek, R. (2001). The biomechanics and motor control of gait in Parkinson disease. Clin. Biomech. *16*, 459–470.

51. Morris, M.E., Iansek, R., Matyas, T.A., and Summers, J.J. (1996). Stride length regulation in Parkinson's disease. Normalization strategies and underlying mechanisms. Brain *119*, 551–568.

52. Mazzone, P., Lozano, A., Stanzione, P., Galati, S., Scarnati, E., Peppe, A., and Stefani, A. (2005). Implantation of human pedunculopontine nucleus: a safe and clinically relevant target in Parkinson's disease. Neuroreport *16*, 1877–1881.

53. Plaha, P., and Gill, S.S. (2005). Bilateral deep brain stimulation of the pedunculopontine nucleus for Parkinson's disease. Neuroreport *16*, 1883–1887.

54. Thevathasan, W., Debu, B., Aziz, T., Bloem, B.R., Blahak, C., Butson, C., Czernecki, V., Foltynie, T., Fraix, V., Grabli, D., et al. (2018). Pedunculopontine nucleus deep brain stimulation in Parkinson's disease: a clinical review. Mov. Disord. *33*, 10–20.

55. Goetz, L., Piallat, B., Bhattacharjee, M., Mathieu, H., David, O., and Chabardès, S. (2016). On the role of the pedunculopontine nucleus and mesencephalic reticular formation in locomotion in nonhuman primates. J. Neurosci. *36*, 4917–4929.

56. Rauch, F., Schwabe, K., and Krauss, J.K. (2010). Effect of deep brain stimulation in the pedunculopontine nucleus on motor function in the rat 6-hydroxydopamine Parkinson model. Behav. Brain Res. *210*, 46–53.

57. Chang, S.J., Cajigas, I., Opris, I., Guest, J.D., and Noga, B.R. (2020). Dissecting brainstem locomotor circuits: converging evidence for cuneiform nucleus stimulation. Front. Syst. Neurosci. *14*, 64.

58. Chang, S.J., Cajigas, I., Guest, J.D., Noga, B.R., Widerström-Noga, E., Haq, I., Fisher, L., Luca, C.C., and Jagid, J.R. (2021). MR tractography-based targeting and physiological identification of the cuneiform nucleus for directional DBS in a Parkinson's disease patient with levodopa-resistant freezing of gait. Front. Hum. Neurosci. *15*, 676755.

59. Noga, B.R., Sanchez, F.J., Villamil, L.M., O'Toole, C., Kasicki, S., Olszewski, M., Cabaj, A.M., Majczyński, H., Sławińska, U., and Jordan, L.M. (2017). LFP oscillations in the mesencephalic locomotor region during voluntary locomotion. Front. Neural Circuits *11*, 34.

60. Lozano, A.M., Lipsman, N., Bergman, H., Brown, P., Chabardes, S., Chang, J.W., Matthews, K., McIntyre, C.C., Schlaepfer, T.E., Schulder, M., et al. (2019). Deep brain stimulation: current challenges and future directions. Nat. Rev. Neurol. *15*, 148–160.

61. Garcia-Rill, E., Saper, C.B., Rye, D.B., Kofler, M., Nonnekes, J., Lozano, A., Valls-Solé, J., and Hallett, M. (2019). Focus on the pedunculopontine nucleus. Consensus review from the May 2018 brainstem society meeting in Washington, DC, USA. Clin. Neurophysiol. *130*, 925–940.

62. Martinez-Gonzalez, C., Bolam, J.P., and Mena-Segovia, J. (2011). Topographical organization of the pedunculopontine nucleus. Front. Neuroanat. *5*, 22.

63. Assous, M., Dautan, D., Tepper, J.M., and Mena-Segovia, J. (2019). Pedunculopontine glutamatergic neurons provide a novel source of feedforward inhibition in the striatum by selectively targeting interneurons. J. Neurosci. *39*, 4727–4737.

64. Raver, C., Uddin, O., Ji, Y., Li, Y., Cramer, N., Jenne, C., Morales, M., Masri, R., and Keller, A. (2020). An amygdalo-parabrachial pathway regulates pain perception and chronic pain. J. Neurosci. *40*, 3424–3442.

65. Roeder, Z., Chen, Q., Davis, S., Carlson, J.D., Tupone, D., and Heinricher, M.M. (2016). The parabrachial complex links pain transmission to descending pain modulation. Pain *157*, 2697–2708.

66. Filli, L., Engmann, A.K., Zörner, B., Weinmann, O., Moraitis, T., Gullo, M., Kasper, H., Schneider, R., and Schwab, M.E. (2014). Bridging the gap: a reticulo-propriospinal detour bypassing an incomplete spinal cord injury. J. Neurosci. *34*, 13399–13410.

67. Engmann, A.K., Bizzozzero, F., Schneider, M.P., Pfyffer, D., Imobersteg, S., Schneider, R., Hofer, A.S., Wieckhorst, M., and Schwab, M.E. (2020). The gigantocellular reticular nucleus plays a significant role in locomotor recovery after incomplete spinal cord injury. J. Neurosci. *40*, 8292–8305.

68. Asboth, L., Friedli, L., Beauparlant, J., Martinez-Gonzalez, C., Anil, S., Rey, E., Baud, L., Pidpruzhnykova, G., Anderson, M.A., Shkorbatova, P., et al. (2018). Cortico–reticulo–spinal circuit reorganization enables functional recovery after severe spinal cord contusion. Nat. Neurosci. *21*, 576–588.

69. Ausborn, J., Shevtsova, N.A., Caggiano, V., Danner, S.M., and Rybak, I.A. (2019). Computational modeling of brainstem circuits controlling locomotor frequency and gait. Elife *8*, e43587.

70. Roussel, M., Lemieux, M., and Bretzner, F. (2020). Using mouse genetics to investigate supraspinal pathways of the brain important to locomotion. In The Neural Control of Movement (Elsevier), pp. 269–313.

71. Cregg, J.M., Leiras, R., Montalant, A., Wanken, P., Wickersham, I.R., and Kiehn, O. (2020). Brainstem neurons that command mammalian locomotor asymmetries. Nat. Neurosci. *23*, 730–740.

72. Usseglio, G., Gatier, E., Heuzé, A., Hérent, C., and Bouvier, J. (2020). Control of orienting movements and locomotion by projection-defined subsets of brainstem V2a neurons. Curr. Biol. *30*, 4665–4681.e6.

73. Stieglitz, L.H., and Prusse, A. (2017). Deep Brain Stimulation in Patients with Incomplete Spinal Cord Injury for Improvement of Gait. Available from: ClinicalTrials.gov. https://clinicaltrials.gov/ct2/show/NCT03053791.

74. Sahel, J.-A., Boulanger-Scemama, E., Pagot, C., Arleo, A., Galluppi, F., Martel, J.N., Esposti, S.D., Delaux, A., de Saint Aubert, J.B., de Montleau, C., et al. (2021). Partial recovery of visual function in a blind patient after optogenetic therapy. Nat. Med. *27*, 1223–1229.

75. Dieter, A., Keppeler, D., and Moser, T. (2020). Towards the optical cochlear implant: optogenetic approaches for hearing restoration. EMBO Mol. Med. *12*, e11618.

76. Lee, B.B., Cripps, R.A., Fitzharris, M., and Wing, P.C. (2014). The global map for traumatic spinal cord injury epidemiology: update 2011, global incidence rate. Spinal Cord *52*, 110–116.

77. Datto, J.P., Bastidas, J.C., Miller, N.L., Shah, A.K., Arheart, K.L., Marcillo, A.E., Dietrich, W.D., and Pearse, D.D. (2015). Female rats demonstrate improved locomotor recovery and greater preservation of white and gray matter after traumatic spinal cord injury compared to males. J. Neurotrauma *32*, 1146–1157.

78. Cao, Z., Hidalgo, G., Simon, T., Wei, S.E., and Sheikh, Y. (2021). OpenPose: realtime multi-person 2D pose estimation using Part Affinity fields. IEEE Trans. Pattern Anal. Mach. Intell. *43*, 172–186.

79. Langford, D.J., Bailey, A.L., Chanda, M.L., Clarke, S.E., Drummond, T.E., Echols, S., Glick, S., Ingrao, J., Klassen-Ross, T., Lacroix-Fralish, M.L., et al. (2010). Coding of facial expressions of pain in the laboratory mouse. Nat. Methods *7*, 447–449.

80. Lemieux, M., Josset, N., Roussel, M., Couraud, S., and Bretzner, F. (2016). Speed-dependent modulation of the locomotor behavior in adult mice reveals attractor and transitional gaits. Front. Neurosci. *10*, 42.

81. Paxinos, G., and Franklin, K.B.J. (2019). Paxinos and Franklin's the Mouse Brain in Stereotaxic Coordinates (Elsevier).

82. VanderHorst, V.G.J.M., and Ulfhake, B. (2006). The organization of the brainstem and spinal cord of the mouse: relationships between monoaminergic, cholinergic, and spinal projection systems. J. Chem. Neuroanat. *31*, 2–36.

83. Mesulam, M.M., Mufson, E.J., Wainer, B.H., and Levey, A.I. (1983). Central cholinergic pathways in the rat: an overview based on an alternative nomenclature (Ch1-Ch6). Neuroscience *10*, 1185–1201.

84. Mena-Segovia, J., Micklem, B.R., Nair-Roberts, R.G., Ungless, M.A., and Bolam, J.P. (2009). GABAergic neuron distribution in the pedunculopontine nucleus defines functional subterritories. J. Comp. Neurol. *515*, 397–408.

85. Schindelin, J., Arganda-Carreras, I., Frise, E., Kaynig, V., Longair, M., Pietzsch, T., Preibisch, S., Rueden, C., Saalfeld, S., Schmid, B., et al. (2012). Fiji: an open-source platform for biological-image analysis. Nat. Methods *9*, 676–682.

STAR★METHODS

KEY RESOURCES TABLE

REAGENT or RESOURCE	SOURCE	IDENTIFIER
**Antibodies**		
Anti-Choline Acetyltransferase polyclonal antibody	Chemicon-millipore	Cat# AB144P; RRID: AB_2079751
Anti-Cre recombinase monoclonal antibody	Millipore	Cat# MAB3120; RRID: AB_2085748
Anti-NeuN polyclonal antibody	Chemicon-Millipore	Cat# ABN78; RRID: AB_10807945
**Bacterial and virus strains**		
AAV2/9 EF1-DIO-hChR2(H134R)-mCherry	Canadian neurophotonics platform	RRID: SCR_016477
AAV2/9 EF1-DIO-hChR2(H134R)-YFP	Canadian neurophotonics platform	RRID: SCR_016477
AAV2/9-EF1a-mCherry-flex-DTA	Canadian neurophotonics platform	RRID: SCR_016477
**Chemicals, peptides, and recombinant proteins**		
Fast Blue	Polysciences	Cat# 17740-5
**Experimental models: Organisms/strains**		
VGlut2-IRES-Cre	The Jackson Laboratory	RRID: IMSR_JAX:016963
**Software and algorithms**		
Graphpad Prism v9.5.0	Graphpad software	https://www.graphpad.com/scientific-software/prism/; RRID: SCR_002798
MATLAB R2013b	Mathworks	https://www.mathworks.com/products/matlab.html; RRID: SCR_001622
Spike 2 v8.06	Cambridge electronic devices	http://ced.co.uk/products/spkovin; RRID: SCR_000903
Zen	Zeiss	http://www.zeiss.com/microscopy/en_us/products/microscope-software/zen.html#introduction; RRID: SCR_013672
Streampix, Digital Video Camera Recording software	Norpix	https://www.norpix.com/products/streampix/streampix.php
MATLAB scripts	Bretzner's lab (for this study)	https://github.com/Malem83/LocoMUA_MLR_SCI

RESOURCE AVAILABILITY

Lead contact
Further information and requests for resources and reagents should be directed to and will be fulfilled by the lead contact, Frederic Bretzner (frederic.bretzner.1@ulaval.ca).

Materials availability
This study did not generate new unique reagents.

Data and code availability
- All data reported in this paper will be shared by the lead contact upon request.
- All original code has been deposited at https://github.com/Malem83/LocoMUA_MLR_SCI and is publicly available as of the date of publication. DOIs are listed in the key resources table.
- Any additional information required to reanalyze the data reported in this paper is available from the lead contact upon request.

EXPERIMENTAL MODEL AND SUBJECT DETAILS

VGlut2-IRES-Cre (RRID: IMSR_JAX:016,963) mouse strain was maintained on a mixed genetic background (129/C57Bl6). Given the prevalence and severity of spinal cord injury in human and rodent males[76] and the weaker functional recovery in comparison to female rodents,[77] adult (≥60 days) male mice weighing approximately 30 g were used in this study. Before experiments, mice were housed in groups with a maximum of 5 per cage. Mice with brain implants were housed individually after surgery to avoid implant damage. AAV2/9 EF1-DIO-hChR2(H134R)-mCherry (titer 9E12 GC/mL to 1.2E13 GC/mL), AAV2/9 EF1-DIO-hChR2(H134R)-YFP (titer 9E12 GC/mL), or AAV2/9-EF1a-mCherry-flex-DTA (titer 1.2E13 CC/mL) was injected in VGlut2-IRES-Cre mice to induce a restricted cre-lox recombination (Canadian Neurophotonics Platform Viral Vector Core Facility (RRID: SCR_016477)). Housing, surgery, behavioral experiments, and euthanasia were performed in compliance with the guidelines of the Canadian Council on Animal Care and approved by the local committee of Université Laval (CPAUL3).

METHOD DETAILS

AAV injections and optical fiber implantations

Under isoflurane (1.5%–2% O2) anesthesia, the mouse was installed in a stereotaxic frame; a craniotomy was performed for AAV injection and chronic implantation of a unilateral optical fiber (diameter: 200 μm) above the nucleus of interest in photostimulated groups. The targets were the cuneiform nucleus (CnF; anteroposterior from the bregma (AP), −4.6 mm; mediolateral (ML), 1.25 mm; depth, 2.2 mm) or the pedunculopontine nucleus (PPN; AP, −4.6 mm; ML, 1.25 mm; depth, 3.25 mm). Chronically implanted groups underwent the AAV injection (100 nL) and the subsequent implantation of an optical fiber in the same surgery. A glass micropipette (WPI, ID: 0.53 and OD: 1.19 mm) was backfilled with mineral oil and fixed on a nano-injector (Nanoliter 2010 Injector, WPI). The pipette was lowered slowly into the nucleus of interest. After a 5-min period, the AAV was injected at a rate of 50 nL/min. To avoid any leakage of the AAV, the glass pipette was held in place for 5 min following the injection before being slowly retracted. The optical fiber (200 μm) was held in place with dental acrylic (cat# 525000 and 526,000, A-M Systems) and machine screw (cat#19010-10, FST, North Vancouver, Canada).

Spinal cord injury

Under isoflurane (1.5%–2% O2) anesthesia, the mouse was installed in a stereotaxic frame; a laminectomy was performed with or without (SHAM group) spinal lesion. The enlargement of vertebra T7 was used as a landmark and T7 was removed using a Friedman-Pearson rongeur to access the T8-T9 spinal segments. In SCI animals, the left spinal cord was transected dorso-ventrally (the medio-lateral landmark was the posterior spinal vein) using a 30G needle. To prevent regeneration, a piece of absorbable cellulose gauze hemostat was inserted in the lesion site. Axial muscles and skin were replaced and sutured. For all surgical procedures, analgesics (Buprenorphine hydrochloride SR: 5 mg/kg) were provided at the end of the surgery for long duration release. After a 1-week recovery, mice were tested in the laboratory.

Retrograde tracing

10 adult VGlut2-Cre mice were exposed to a laminectomy and 16 VGlut2-Cre mice to a laminectomy followed by a lateral thoracic hemisection. 8 weeks after SCI or sham surgery, under isoflurane (1.5%–2% O2) anesthesia, the mouse was installed in a stereotaxic frame; a craniotomy was performed for retrograde tracer injection: Fast Blue (Polysciences, 17,740-5, 50 nL, 2% suspension in phosphate buffer with 2% dimethyl sulfoxide) in the contralesional medulla: AP = 5.5 mm; L = 0.5 mm; D = 5.5 mm. A 2 μL Hamilton neuro syringe was used to perform this injection. To avoid leakage, the neuro syringe was kept in place for 2 min and removed slowly after the injection. Mice were perfused 4 days after the Fast Blue injection. Mice with SCI extending over the other side of the spinal cord and Fast Blue injections leaking in the ipsilesional brainstem were excluded from our analysis. We analyzed a total of 5 SCI and 5 sham mice for our tracing experiments.

Locomotor tests

Mice were tested during the day in a room dedicated to behavioral experiments. Before any surgery, mice were trained to swim and walk on a treadmill. During swimming, treadmill, and spontaneous walking, reflective markers were placed on each joint of the left ipsilesional hindlimb (iliac crest, hip, knee, ankle, and toe). Mice were filmed on both sides with high-frequency cameras (Genie HM640, Dalsa Teledyne; 250 frames/s). In the open field test, mice were filmed from above (100 frames/s) and a reflective marker was placed on their neck to track body movements. Videos were digitized with StreamPix 6.0 (Norpix) and analyzed offline using custom-designed software, OpenPose,[78] and MATLAB.

Mice learned to swim in straight lines and reach a platform at the end of a transparent corridor (length: 53 cm, width: 5 cm, height: 18 cm). To prevent hypothermia, the temperature of the water was monitored and maintained between 23 and 25°C. Three to four laps were analyzed per mouse (with or without photostimulation).

We tested spontaneous overground walking in a transparent corridor (length: 60, width: 5 cm, height: 25) connecting two open boxes—one transparent and one painted black. Mice were placed in the brighter box and crossed the corridor to find refuge in the darker box. Three to four passages were analyzed per mouse (with or without photostimulation).

Kinematics and EMGs recordings were performed during two separate sessions on a treadmill (Exer 3/6 treadmill, Columbus instruments). During EMG experiments, mice were filmed from both sides at 100 frames/s without any markers on the hindlimb. Five photostimulations were analyzed per mouse. During open field experiments (a rectangular treadmill surface (38 × 43 cm), mice were videotaped from above.

A locomotor score was performed blindly by an experimenter to monitor spontaneous motor recovery after lateral thoracic hemisection.[40] Hindlimbs were scored individually each week after SCI. This non-linear and summative score on a 20-point scale takes into consideration the articular coordination, the weight support, the digit position, the foot placement while stepping, the tail position, and the fore-hindlimb coordination. One experimenter evaluated stress and discomfort before and during 1 s trains of photostimulation at rest using a 10-point grimace scale scoring five facial expressions: orbital tightening, nose bulge, cheek bulge, ear position, and whisker change.[79]

Genetic ablation of glutamatergic neurons

A total of 11 mice were used. Chronic SCI mice were split evenly according to their locomotor score 8 weeks after SCI into two groups prior to genetic ablation of glutamatergic neurons of the right CnF or PPN.[41,42] 100 nL injection of AAV2/9-EF1a-mCherry-flex-DTA (titer 1.2E13 CC/mL) was injected under isoflurane anesthesia during stereotaxic surgery in either the right CnF (n = 6 mice) or PPN (n = 5 mice). mCherry labeling was used to assess the extent of the injection site and confirm that they were circumscribed to the targeted nucleus. The number of NeuN+ and ChAT + labeled neurons was counted over 5 sections at the level of the injection site in both left and right CnF and PPN nuclei (see immunochemistry section). For behavioral tests, 7 to 10 consecutive step cycles per mouse were analyzed for treadmill locomotion before and after AAV-DTA injection; 3 swimming laps per mouse were combined for analysis of 30–45 swim cycles (∼11 swim cycles per lap) before and after AAV-DTA injection.

Optogenetic and electrophysiological recordings

A total of 14 adult mice were assessed upon optogenetic manipulation before SCI. 2 to 3 mice lost their implant in the CnF within the 7-week period of recording after SCI. So, 5/7 mice were assessed during treadmill locomotion and 4/7 during the corridor test upon optogenetic CnF stimulation until 7 weeks after SCI. 7/7 mice were assessed for all locomotor tests upon optogenetic PPN stimulation. Before and after SCI and throughout all locomotor tests, optical manipulations of Channelrhodopsin-2.0 (ChR2)-expressing neurons were undertaken in freely behaving mice. The pattern and timing of optical manipulations were controlled using a mechanical shutter (Connectorized Mechanical Shutter Adapters; Doric, Canada) and controller (SR470 Laser Shutter Controller; Stanford Research Systems, California, USA) synchronized online during kinematic and EMG recordings. Channelrhodopsin-2.0-expressing neurons (ChR2) were photostimulated by using a blue laser (50 mW power, 473nm wavelength, Laserglow Technologies, ON, Canada). Before SCI, a threshold was determined to reliably evoke behavioral responses in each mouse for each locomotor test in 3 separate sessions of photostimulation, 1 week apart each. During EMG recordings, the laser power threshold was determined using motor responses evoked upon 10 ms pulse photostimulations delivered to the animal at rest. Pulses were delivered during treadmill locomotion at the defined threshold (between 1 and 18 mW) at steady locomotor speed (10 cm/s). Regarding the timing of stimulations, continuous 10 ms pulses were delivered every 3 s to assess changes in locomotor pattern (total of 150 pulses); trains of 10 ms pulses at 5-10-20-50 Hz were also used for a duration of 1 s every 5 s.

EMG activity of the tibialis anterior (TA, ankle flexor), gastrocnemius lateralis (GL, ankle extensor) muscles were recorded using acute electrodes. Mice were anesthetized with isoflurane (1.5–2%) to insert dual core wires into muscles of interest, as previously described elsewhere.[10,33] Recordings were made when mice were fully awake and ready to walk. EMG signals were amplified (x 1000), band-pass filtered (0.1–10 kHz), sampled at 10 kHz, and digitally converted (Power 1401; CED, Cambridge, UK) using Spike2 version 8 (CED, Cambridge, UK). EMG signals were high-pass filtered, rectified, and analyzed offline using custom-designed software and MATLAB.

Kinematic analysis

As previously described,[10,33] joint markers of the iliac crest, hip, ankle, and toe were detected. To avoid skin slippage, the knee was inferred by triangulation using the length of the femur and the tibia. Hindlimb joints were detected using the machine learning software OpenPose.[78] All OpenPose predictions were manually verified and corrected if needed on custom-designed software.[80] Custom MATLAB scripts were used to analyze joints kinematics. Using custom-made software, the beginning of the stance (contact on the ground) and the beginning of the swing (lift from the ground) phases of the hindlimbs during treadmill locomotion were manually tagged. Intralimb angular coupling (i.e., hip angle versus ankle angle) was defined as a phase of the maximum of the ankle angle according to the maximum of the hip angle. Regarding swimming, the swim cycle was divided into a power and return stroke which were defined using the evolution of the hip angle during swimming. The power stroke ranges from the minimum to the maximum of the hip angle; the return stroke ranges from the maximum to the minimum angle.

EMG analysis

Motor spikes were extracted from raw EMG recordings using a threshold of 5 times the mean of the background signal at rest. Then, a custom-made MATLAB script was used to identify the beginnings and endings of step cycles using time-sensitive changes in motor spike density. For each hindlimb, the reference muscle was the tibialis anterior. The step cycle was divided in 5 equal epochs, the first

two corresponding to the active phase of the flexor (associated with the swing phase of the limb) and the last three corresponding to the active phase of the extensor (associated with the stance phase of the limb). Changes in motor spike density and mean amplitude were assessed as the difference between a 50 ms time window before and after 10 ms photostimulation. For the analysis of trains of photostimulation or unstimulated locomotion, the burst of activity of the muscles (and the step cycles) were selected manually in Spike2.

Perfusion

Mice were deeply anesthetized and transcardially perfused with 10 mL saline (0.9% NaCl) followed by 20 mL paraformaldehyde (4% PFA). Tissues (spinal cord and brain) were harvested and post-fixed overnight in 4% PFA, then in 30% sucrose until saturation. Tissues (spinal cords and brains) were frozen in Leica tissue freezing medium, then cut on a Leica cryostat (20 μm slices, Leica CM1860, Germany).

Immunochemistry

Immunostainings were performed on brainstem slices to confirm the position of the optical probe, the extent of the cre-lox recombination, and analyzed retrogradely labeled neurons from tracing experiments. The following primary antibodies were used: anti-choline acetyltransferase (ChAT) 1:100 (Chemicon-Millipore, AB144P), anti-Cre recombinase (CRE) 1:1000 (EMD Millipore, MAB3120), and the anti-NeuN 1:500 (Chemicon-Millipore, ABN78). The following secondary antibodies were used: donkey anti-mouse-AF594 1:1000 (Thermofisher Scientific, A-21203), donkey anti-goat-AF488 1:1,000 (Abcam, AB150129) donkey anti-rabbit-AF594 (Invitrogen, A21207), and donkey anti-rabbit-AF350 (Invitrogen, A10039).

To confirm optical probe location and cre-lox recombination extent, images were taken on an Axio Imager M2 microscope connected to an AxioCam camera using ZEN2 software (Zeiss, Germany). Low-magnification reconstructions were generated to delineate the extent of the cre-lox recombination and determine the stereotaxic coordinates of the tip of the optical cannula according to anatomical landmarks (superior cerebellar peduncle, inferior colliculus, and the periaqueductal gray) and anatomical atlases.[81,82] As previously reported.[38,83,84] cholinergic immunostaining was used to identify and localize the cholinergic PPN. The cholinergic staining and the extent of cre-lox recombination were evaluated by outlining the area on low-magnification reconstructions to determine whether the stimulation site was located within the CnF or PPN. Spinal cord lesions were imaged using brightfield microscopy. Using Fiji,[85] the extents of the spinal cord lesion were evaluated on three subsequent slices at the epicenter of the lesion. The area of spared tissue was compared to adjacent intact sections, the extent of the lesion ranged from 40% to 65%.

Stereology

For tracing experiments, animals in which Fast Blue injection was circumscribed to the right medulla including the gigantocellular reticular nucleus (GI), the lateral paragigantocellular reticular nucleus (LPGi), the alpha and ventral pars of the gigantocellular reticular nucleus (GiV/α) we included for analysis. Every two slices were imaged using an epifluorescence microscope (Olympus BX51, Tokyo, Japan) associated with stereology software (Stereo Investigator, MicroBrightField Bioscience, Colchester, VT). Using anatomical landmarks, the borders of the CnF and PPN were traced. A random subsampling was used, and neurons were counted in selected sections (50–150 μm^2 squares). Sizes of the sections were determined by the surface on the slices and the volume of the nucleus. Taking in consideration the number of counted cells, the interval between sections, the area of the nucleus, and the thickness of the slices, the number of counted cells was estimated. In some experiments, all neurons were counted manually every other section enabling a 3D representation of the PPN and CnF (Figure 2C). Custom MATLAB scripts enabled pooling all mice together and representing the spatial distribution (medio-lateral and dorsoventral) of retrogradely labeled neurons.

QUANTIFICATION AND STATISTICAL ANALYSIS

Information about mice numbers, statistical tests, and data representation can be found in Fig. legends. Data are represented as mean ± SD and statistical difference was indicated by asterisks (*$p \leq 0.05$, **$p < 0.01$, ***$p < 0.001$, ****$p < 0.0001$). Before every analysis, the normality of the data distribution was assessed using a Shapiro-Wilk test. To test statistical difference from a specified value, we used a one-sample t-test if the distribution was normal or a Mann-Whitney test if the distribution was not normal. To compare groups, a one-way ANOVA was performed followed by a Dunnett's post-test if the distribution was normal. Otherwise, if the distribution was not normal, a Kruskall-Wallis test was performed with a Dunn's multiple comparison post-test. To compare paired groups, we used repeated measures one-way ANOVA followed by Tukey's comparison test for normally distributed values and a Friedman test followed by Dunn's multiple comparisons test otherwise. Boxplots represent the 25th percentile, the median, the 75th percentile, and the whiskers the maximum and minimum values.

In order to evaluate step-by-step variability (Figures 7C and S12G), joint angles of subsequent step (or swim) cycles were averaged. Each step cycle duration was normalized and divided in 512 bins. Photostimulation changed the dynamics of the step cycle (shortening the stance phase and the step cycle duration) thus preventing a bin-to-bin comparison between pre- and photostimulated conditions. In order to focus on the swing phase variability, the coefficient of variation was calculated for each bin located between the maximal opening of the hip angle (beginning of the swing) and the end of the step cycle (foot contact). Coefficients of variation of swing phase bins were pooled and treated as a parameter and compared between pre- and photostimulated conditions with either a

one-sample t-test or a Mann-Whitney according to normality of data. Variability for both return and power strokes were analyzed during swimming.

For quantification of the percentage of mice showing improvement, deficit, or absence of change in locomotor parameters, significant increases were considered as an improvement when there was a gain of locomotor function (i.e., an increase in angular excursion, stride length, step height, power-stroke, swim cycle frequency and swim speed in Figure 3; speed, iliac crest height, step height in Figure 6; integrated amplitude in Figures 7 and S11; speed, locomotor frequency, angular excursion in Figures S12 and S13). In the case of burst duration (Figures 7 and S11) and coefficient of variation (Figures 7 and S12), significant decreases were also considered as an improvement. In contrast, a significant decrease in impairing or reducing locomotor functions was considered as a deficit.

Cell Reports Medicine, Volume *4*

Supplemental information

Functional contribution of mesencephalic locomotor region nuclei to locomotor recovery after spinal cord injury

Marie Roussel, David Lafrance-Zoubga, Nicolas Josset, Maxime Lemieux, and Frederic Bretzner

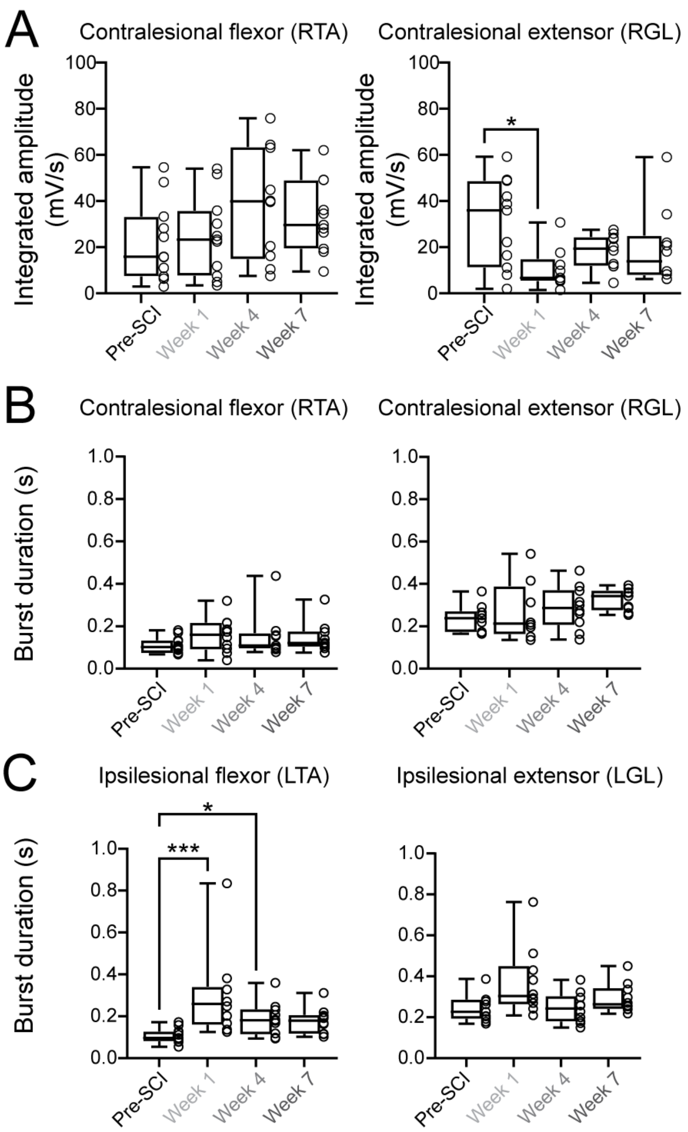

Fig. S1: Background EMG activity in the contralesional flexor and extensor muscles after SCI. Related to Figure 1.

(A) Integrated amplitude of the contralesional right flexor and extensor (n = 11 mice, Kruskal-Wallis test [$p = 0.049$] with Dunn's multiple comparisons test, *$p < 0.05$).

(B-C) Burst duration of the contra- (B) and ipsilesional (C) flexor and extensor muscles (n = 11 mice, LTA Kruskal-Wallis test [$p = 0.0007$] with Dunn's multiple comparisons test, *$p < 0.05$, ***$p < 0.001$; LGL Kruskal-Wallis test [$p = 0.0428$] with Dunn's multiple comparisons test).

Abbreviations: LTA /RTA, left/right tibialis anterior, LGL/RGL, left/right gastrocnemius lateralis.

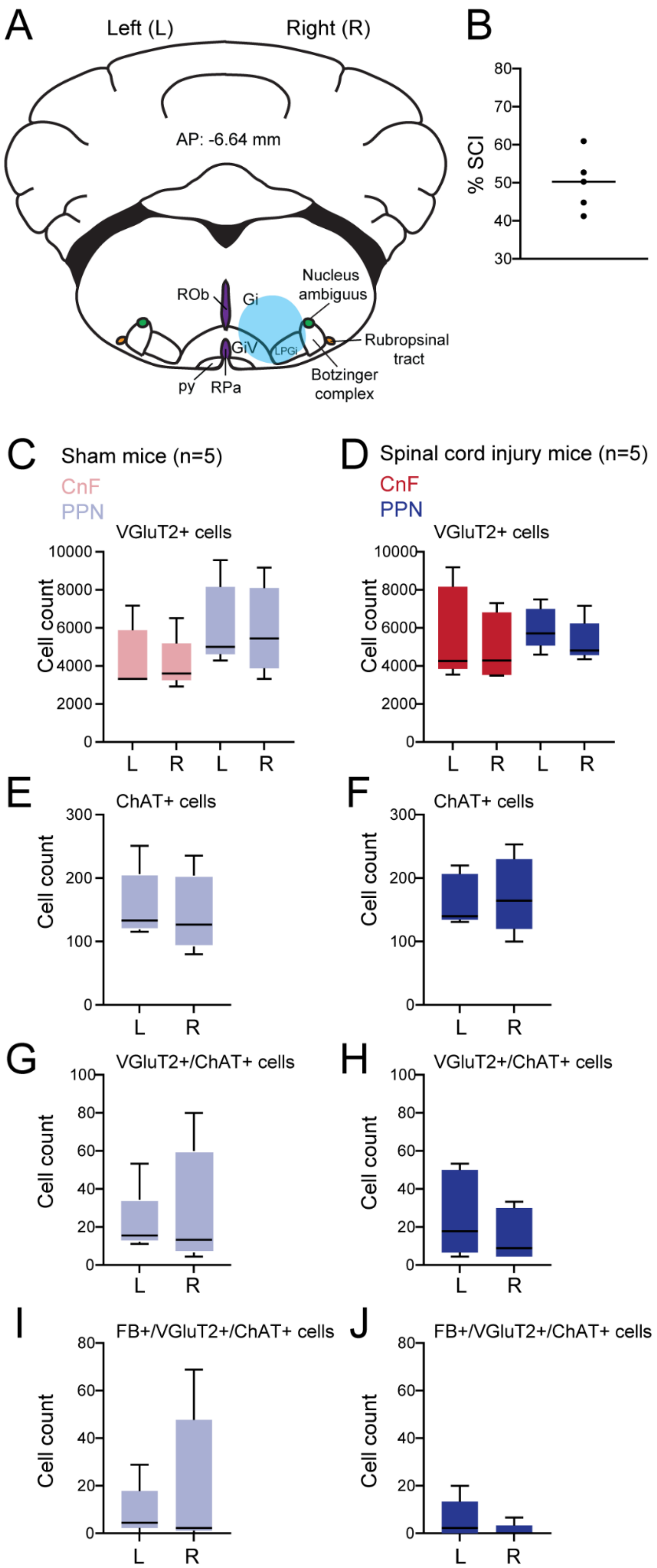

A
Left (L)
Right (R)
AP: -6.64 mm
ROb
Gi
Nucleus ambiguus
GiV
LPGi
Rubropsinal tract
py
RPa
Botzinger complex
B
% SCI
C
Sham mice (n=5)
CnF
PPN
VGluT2+ cells
Cell count
D
Spinal cord injury mice (n=5)
CnF
PPN
VGluT2+ cells
E
ChAT+ cells
F
ChAT+ cells
G
VGluT2+/ChAT+ cells
H
VGluT2+/ChAT+ cells
I
FB+/VGluT2+/ChAT+ cells
J
FB+/VGluT2+/ChAT+ cells
L
R

Fig. S2: Cell counts of glutamatergic, cholinergic, and glutamatergic/cholinergic CnF and PPN neurons after chronic SCI. Related to Figure 2.

(A) Fast Blue injections in the gigantocellular reticular nucleus (Gi), the lateral paragigantocellular reticular nucleus (LPGi), and the alpha and ventral pars of the gigantocellular reticular nucleus (GiV/α).

(B) Extent of the spinal cord injury.

(C-D) Number of VGluT2:cre+ neurons in left (L) ipsi- and right (R) contralesional CnF and PPN of Sham (C) and SCI (D) mice.

(E-F) Number of ChAT+ neurons in left (L) ipsi- and right (R) contralesional PPN of Sham (E) and SCI (F) mice.

(G-H) Number of VGluT2:cre+/ChAT+ neurons in left (L) ipsi- and right (R) contralesional PPN of Sham (G) and SCI (H) mice.

(I-J) Number of Fast-Blue+/VGluT2:cre+/ChAT+ neurons in left (L) ipsi- and right (R) contralesional PPN of Sham (I) and SCI (J) mice.

Mann-Whitney tests (C-J), L (left) vs R (right), $p > 0.05$.

Abbreviations: Gi: gigantocellular reticular nucleus, GiV: gigantocellular reticular nucleus ventral part, LPGi: lateral paragigantocellular nucleus, L: left, R: right, py: pyramidal tract, ROb: raphe obscurus nucleus, RPa: raphe pallidus nucleus.

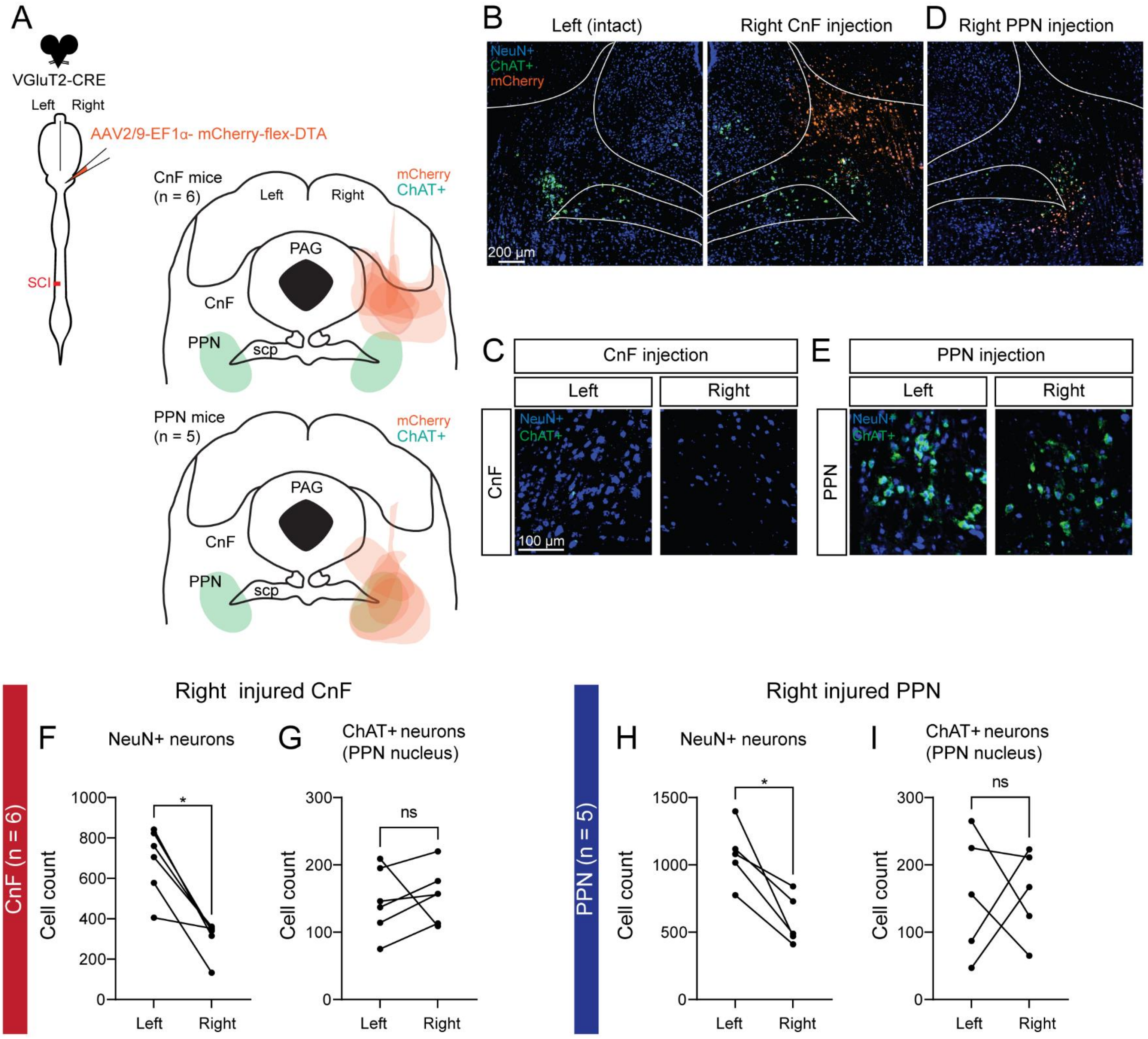

Fig. S3: Genetic ablation of glutamatergic CnF or PPN neurons. Related to Figure 3.

(A) Injection of AAV-EF1a-mCherry-flex-DTA in the right CnF or PPN of VGlut2-cre mice 9 weeks after SCI. Overlap of mCherry expression (orange), neuronal nuclear protein (NeuN in blue), and Choline AcetylTransferase (ChAT in green) staining.

(B) Low magnification images illustrating sections at the level of the left and right MLR nuclei upon genetic ablation of glutamatergic neurons of the right CnF. mCherry expression (orange) illustrates the extent of the AAV injection, NeuN (blue) staining identifies all neurons, and ChAT (green) staining identifies cholinergic PPN neurons.

(C) High magnification images of B with immunostaining against NeuN (blue) and ChAT (cholinergic neurons, green) at the level of the CnF.

(D) Low magnification image illustrating a section of right MLR nuclei upon genetic ablation of glutamatergic neurons of the right PPN.

(E) High magnification images of D with immunostaining against NeuN (blue) and ChAT (cholinergic neurons, green) at the level of the PPN.

(F) Number of NeuN positive neurons in the left and right CnF upon injection of the right CnF ($n = 6$ mice, one-tailed test, Wilcoxon matched-pairs signed rank test, $p = 0.02$).

(G) Number of cholinergic neurons in the left and right PPN upon injection of the right CnF ($n = 6$ mice, one-tailed test, Wilcoxon matched-pairs signed rank test, $p = 0.22$).

(H) Number of NeuN positive neurons in the left and right PPN upon injection of the right PPN ($n = 5$ mice, one-tailed test, Wilcoxon matched-pairs signed rank test, $p = 0.03$).

(I) Number of cholinergic neurons in the left and right PPN upon injection of the right PPN ($n = 5$ mice, one-tailed test, Wilcoxon matched-pairs signed rank test, $p = 0.5$).

Abbreviations: ChAT: Choline Acetyltransferase, CnF: Cuneiform nucleus, PPN: Pedunculopontine nucleus, PAG: Periaqueductal Gray, SCP: Superior Cerebellar Peduncle.

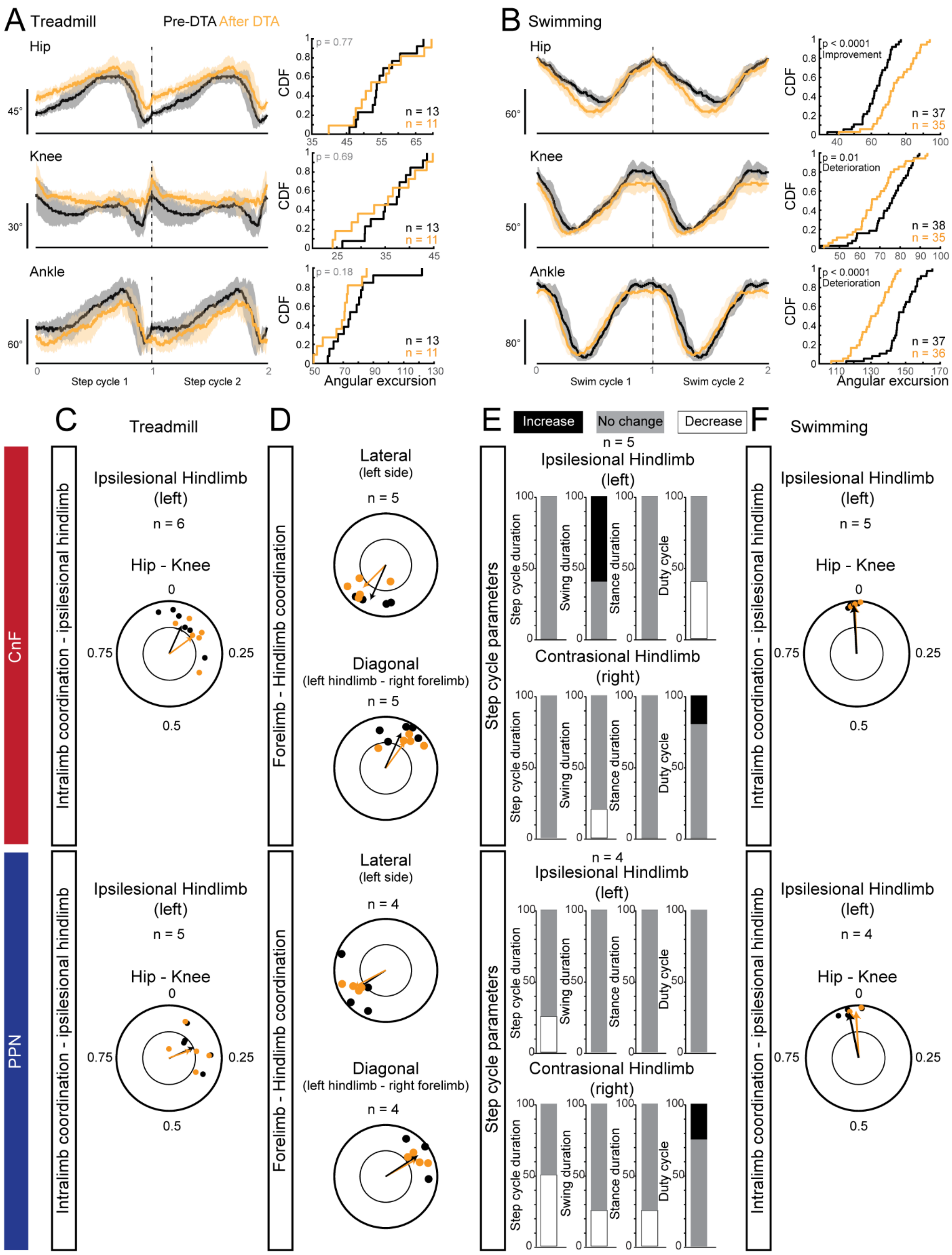
A Treadmill
Pre-DTA After DTA
Hip
Knee
Ankle
45°
30°
60°
Step cycle 1
Step cycle 2
CDF
p = 0.77
p = 0.69
p = 0.18
n = 13
n = 11
Angular excursion
B Swimming
60°
50°
80°
Swim cycle 1
Swim cycle 2
p < 0.0001
Improvement
p = 0.01
Deterioration
p < 0.0001
Deterioration
n = 37
n = 35
n = 38
n = 35
n = 37
n = 36
C Treadmill
D
E
Increase
No change
Decrease
F Swimming
CnF
PPN
Intralimb coordination - ipsilesional hindlimb
Ipsilesional Hindlimb (left)
n = 6
n = 5
n = 4
Hip - Knee
0
0.25
0.5
0.75
Forelimb - Hindlimb coordination
Lateral (left side)
Diagonal (left hindlimb - right forelimb)
Step cycle parameters
Contrasional Hindlimb (right)
Step cycle duration
Swing duration
Stance duration
Duty cycle

Fig. S4: Intralimb coordination during locomotion upon genetic ablation. Related to Figure 3.

(A) Mean and SD of hip, knee, and ankle joint angles of the ipsilesional hindlimb during treadmill locomotion before and after genetic ablation of glutamatergic PPN neurons in a mouse. Cumulative distribution function (CDF) of the angular excursion of hindlimb joints (n = step cycles, Mann-Whitney test or unpaired t-test according to the normality of the distribution).

(B) Mean and SD of hip, knee, and ankle joint angles of the ipsilesional hindlimb during swimming before and after genetic ablation of glutamatergic PPN neurons (same mouse as in A). Cumulative distribution function (CDF) of the angular excursion of hindlimb joints (n = swim cycles, Mann-Whitney test or unpaired t-test according to the normality of the distribution).

(C) Intralimb coupling between the hip and knee during treadmill locomotion before and after genetic ablation of glutamatergic neurons of the CnF or PPN. Each circle in polar plots represents a mouse.

(D) Lateral coupling between left fore-and hindlimb (anchored on the left forelimb) and diagonal coupling between left hindlimb and right forelimb (anchored on the right forelimb). Each circle in polar plots represents a mouse.

(E) Percentage of mice exhibiting a significant increase, decrease, or absence of change in step cycle duration, swing and stance phase duration, and duty cycle of the stance phase (Mann-Whitney test or unpaired t-test according to the normality of the distribution).

(F) Intralimb coupling between the hip and knee during swimming. Each circle in polar plots represents a mouse.

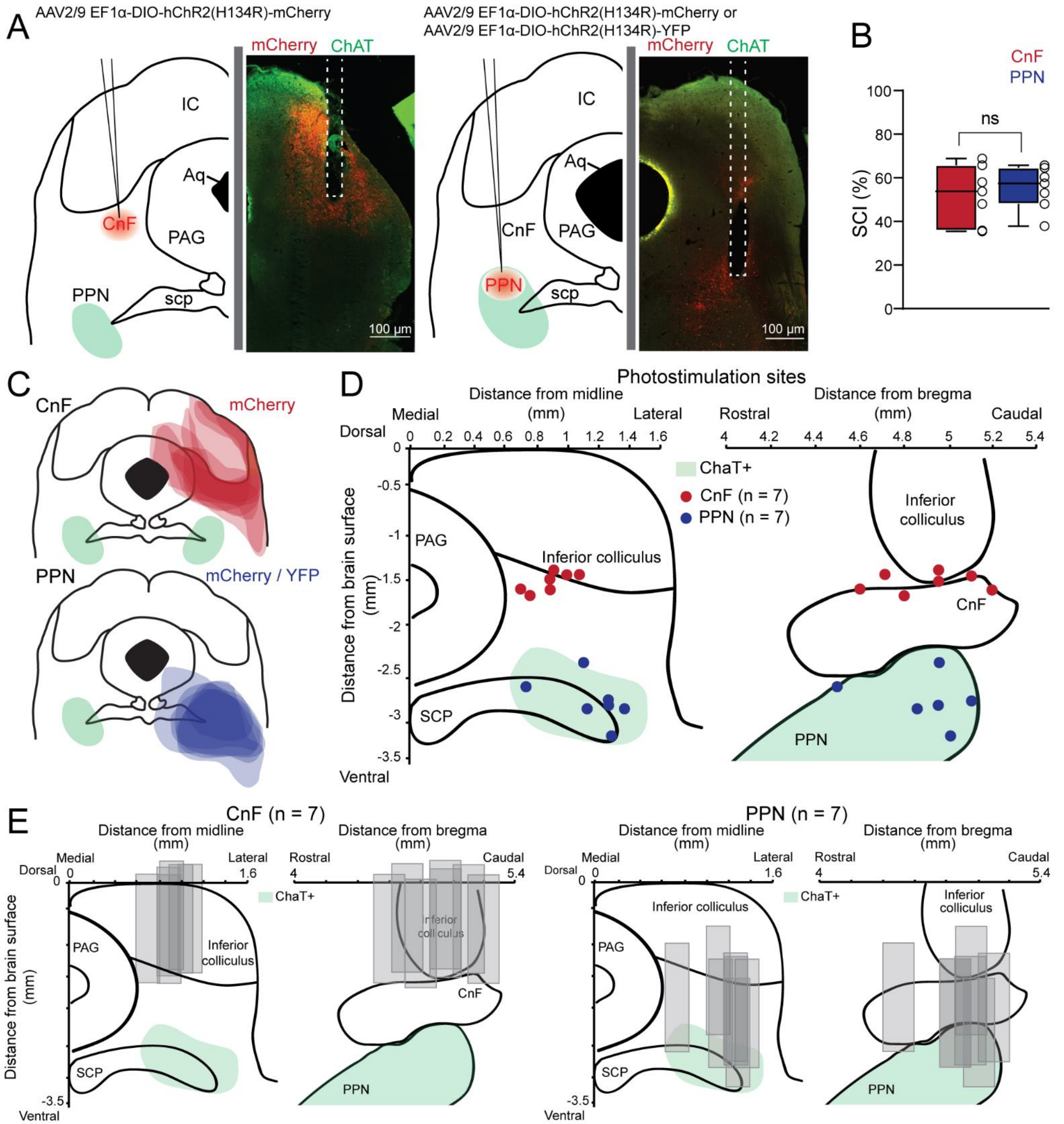
A
AAV2/9 EF1α-DIO-hChR2(H134R)-mCherry
mCherry ChAT
IC
Aq
CnF
PAG
PPN
scp
100 µm
AAV2/9 EF1α-DIO-hChR2(H134R)-mCherry or
AAV2/9 EF1α-DIO-hChR2(H134R)-YFP
B
CnF
PPN
ns
SCI (%)
C
mCherry
mCherry / YFP
D
Photostimulation sites
Distance from midline (mm)
Medial
Lateral
Dorsal
Ventral
Distance from brain surface (mm)
ChaT+
CnF (n = 7)
PPN (n = 7)
Inferior colliculus
SCP
Distance from bregma (mm)
Rostral
Caudal
E
CnF (n = 7)
PPN (n = 7)

Fig. S5: Extent of the cre-lox recombination, site of optical cannulas, and extent of the lesion site. Related to Figure 4.

(A) Examples of injection sites with AAV-EF1a-DiO-ChR2-mCherry/YFP in the CnF or PPN showing the extent of the cre-lox recombination with mCherry or YFP reporter expression.

(B) Lesion as percentage of the surface of the transverse section at the thoracic spinal level (n = 7 CnF and 7 PPN, unpaired t-test, p = 0.64).

(C) Mapping of injection spread. For each mouse (n = 7 CnF and 7 PPN), the infection site was identified and segmented as a series of coordinates forming contour maps. Maps were superimposed with overlapping areas showing a darker shade. Mice were injected with AAV-EF1-DiO-ChR2-mCherry or AAV-EF1-DiO-ChR2-YFP to target the ventral PPN (blue), and the CnF (red).

(D) Anatomical localization of the tip of the optical fibers at the level of the CnF and the caudal PPN.

(E) Anatomical fiber localization for the cohort presented in D. Fiber tips are represented for each mouse.

Abbreviations: Aq: Aqueduct, ChAT: Choline Acetyltransferase, CnF: Cuneiform nucleus, C: Inferior Colliculus, PPN: Pedunculopontine nucleus, PAG: Periaqueductal Gray, SCP: Superior Cerebellar Peduncle, YFP: Yellow Fluorescent Protein.

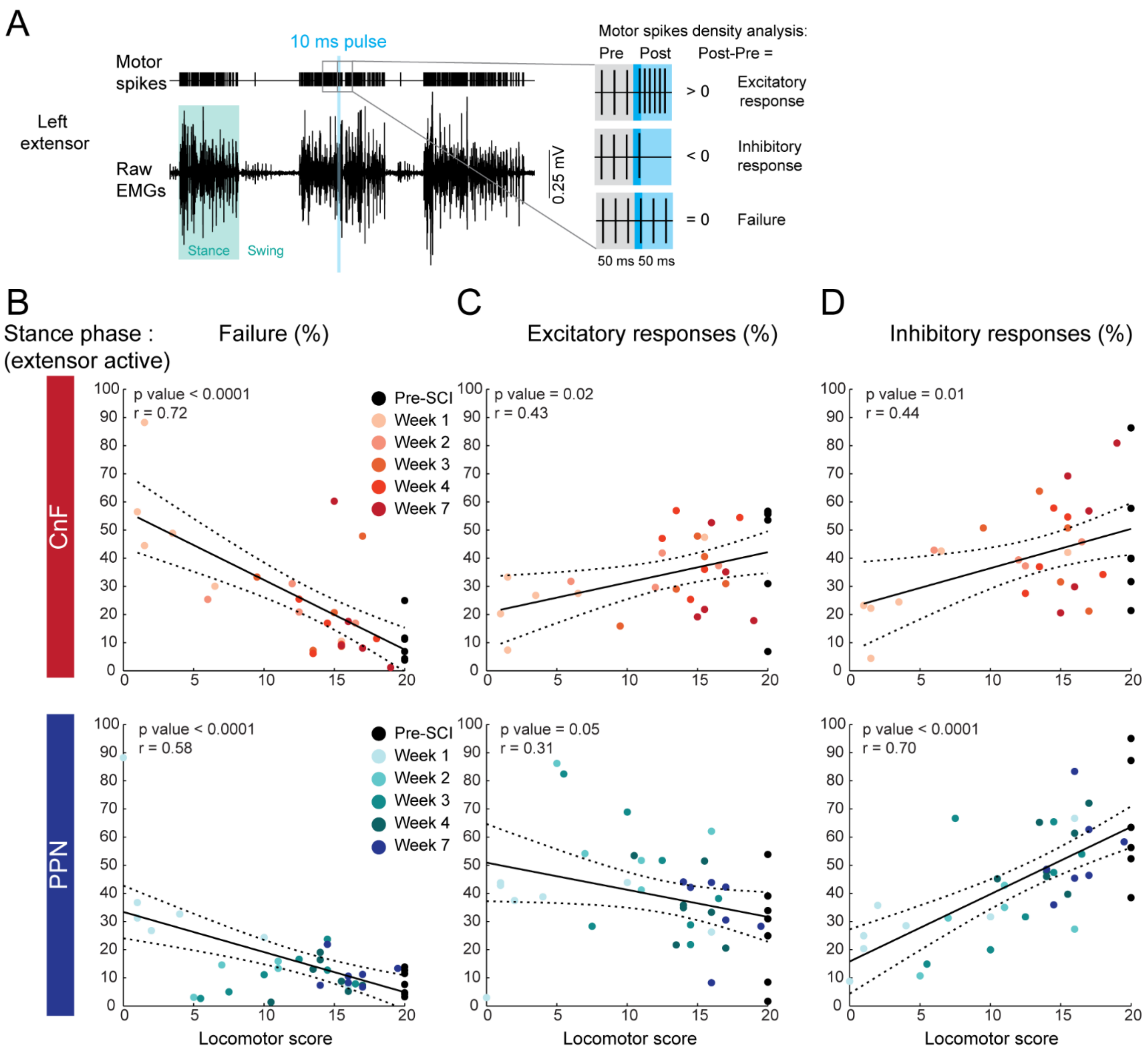

Fig. S6: Changes in the proportion of failure, excitatory, and inhibitory motor responses in the ipsilesional extensor muscle during the stance phase after SCI. Related to Figure 4.

(A) Example of changes in motor spike density upon photostimulation.

(B-D) Proportion of failure (B), excitatory (C), and inhibitory (D) motor responses evoked in the ipsilesional extensor during the stance phase as a function of the locomotor score of the ipsilesional hindlimb over the course of recovery after SCI (n = 6 CnF and n = 7 PPN per week, Linear regressions (solid lines) and 95% confidence interval (dashed lines)).

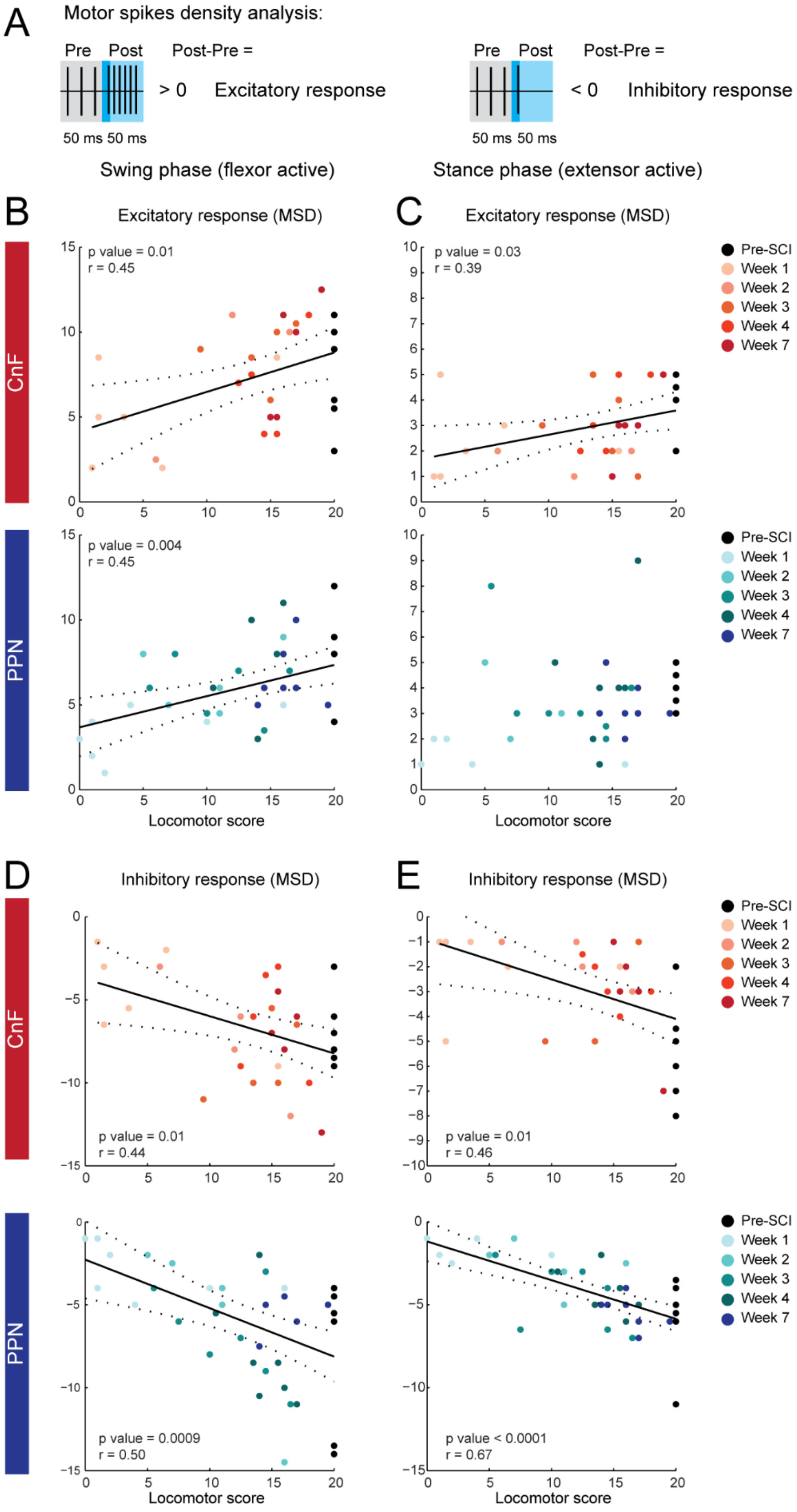
A
Motor spikes density analysis:
Pre
Post
Post-Pre =
> 0
Excitatory response
50 ms 50 ms
Pre
Post
Post-Pre =
< 0
Inhibitory response
50 ms 50 ms
Swing phase (flexor active)
Stance phase (extensor active)
B
Excitatory response (MSD)
C
Excitatory response (MSD)
CnF
p value = 0.01
r = 0.45
p value = 0.03
r = 0.39
Pre-SCI
Week 1
Week 2
Week 3
Week 4
Week 7
PPN
p value = 0.004
r = 0.45
Locomotor score
Locomotor score
D
Inhibitory response (MSD)
E
Inhibitory response (MSD)
p value = 0.01
r = 0.44
p value = 0.01
r = 0.46
p value = 0.0009
r = 0.50
p value < 0.0001
r = 0.67
Locomotor score
Locomotor score

Fig. S7: Changes in motor spike density of excitatory and inhibitory motor responses evoked in the ipsilesional hindlimb before and after SCI. Related to Figure 4.

(A) Example of changes in motor spike density upon photostimulation.

(B) Motor spike density of excitatory motor responses evoked in the ipsilesional flexor muscle during the swing phase upon short pulse photostimulation delivered above glutamatergic CnF or PPN neurons as a function of the locomotor score of the ipsilesional hindlimb over the course of recovery after SCI (n = 6 CnF and n = 7 PPN per week, Linear regressions (solid lines) and 95% confidence interval (dashed lines)).

(C) Motor spike density of excitatory motor responses evoked in the ipsilesional extensor muscle during the stance phase upon short pulse photostimulation delivered above glutamatergic CnF or PPN neurons as a function of the locomotor score of the ipsilesional hindlimb over the course of recovery (n = 6 CnF and n = 7 PPN per week).

(D) Motor spike density of inhibitory responses evoked in the ipsilesional flexor muscle during the swing phase upon short-pulse photostimulation of glutamatergic CnF or PPN neurons as a function of locomotor score (n = 6 CnF and n = 7 PPN per week).

(E) Motor spike density of inhibitory responses evoked in the ipsilesional extensor muscle during the stance phase upon short-pulse photostimulation of glutamatergic CnF or PPN neurons as a function of locomotor score (n = 6 CnF and n = 7 PPN per week).

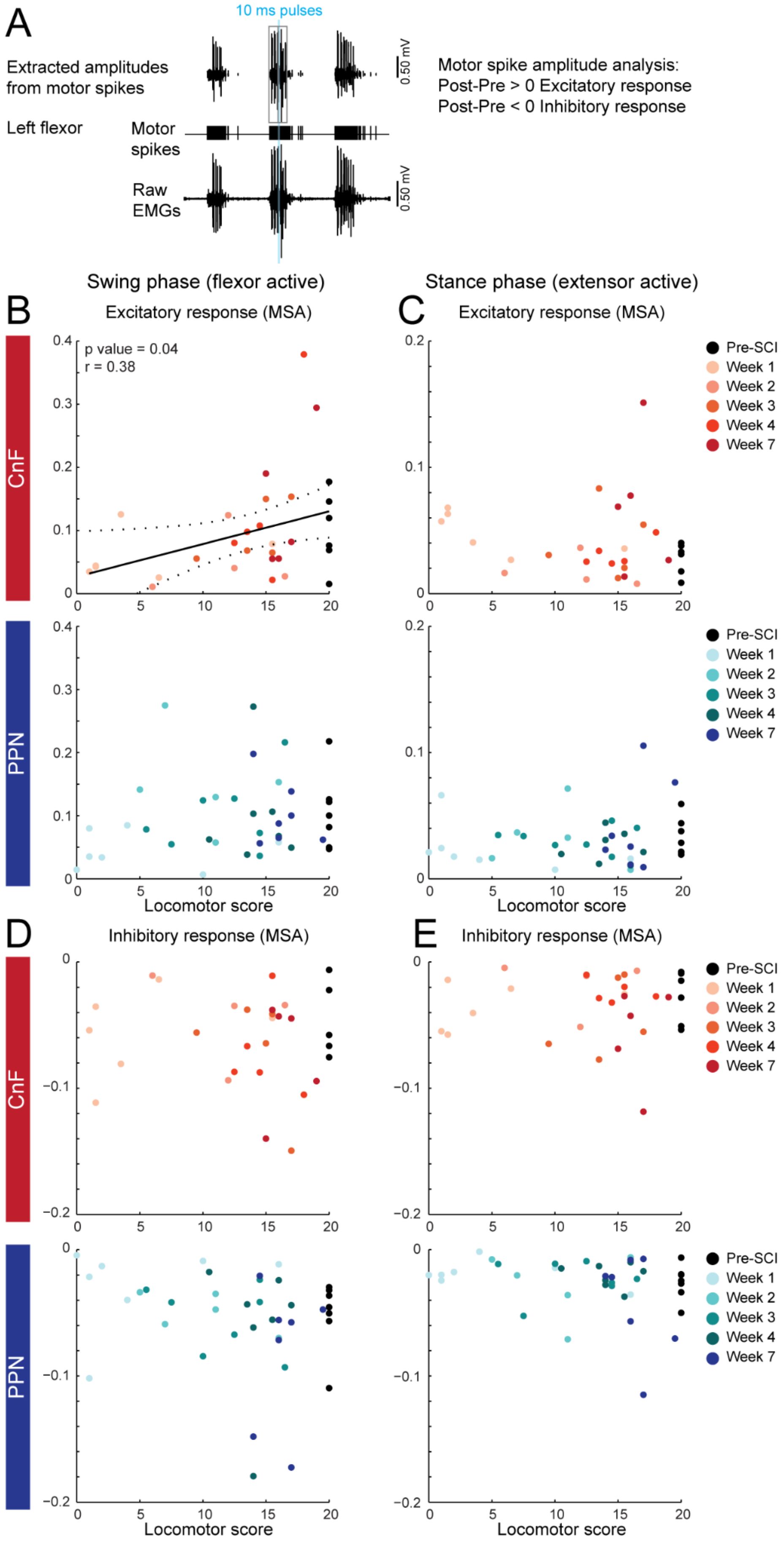

A
10 ms pulses
Extracted amplitudes from motor spikes
0.50 mV
Motor spike amplitude analysis:
Post-Pre > 0 Excitatory response
Post-Pre < 0 Inhibitory response
Left flexor
Motor spikes
Raw EMGs
0.50 mV
Swing phase (flexor active)
Stance phase (extensor active)
B
Excitatory response (MSA)
C
Excitatory response (MSA)
p value = 0.04
r = 0.38
CnF
PPN
Pre-SCI
Week 1
Week 2
Week 3
Week 4
Week 7
Locomotor score
D
Inhibitory response (MSA)
E
Inhibitory response (MSA)

Fig. S8: Changes in motor spike amplitude of excitatory and inhibitory motor responses evoked in the ipsilesional hindlimb before and after SCI. Related to Figure 4.

(A) Example of changes in motor spike amplitude upon photostimulation.

(B) Motor spike amplitude of excitatory responses evoked in the ipsilesional flexor muscle during the swing phase upon short-pulse photostimulation of glutamatergic CnF or PPN neurons as a function of locomotor score (n = 6 CnF and n = 7 PPN per week, linear regression (solid line) and 95% confidence interval (dashed lines)).

(C) Motor spike amplitude of excitatory responses evoked in the ipsilesional extensor muscle during the stance phase upon short-pulse photostimulation of glutamatergic CnF or PPN neurons as a function of locomotor score (n = 6 CnF and n = 7 PPN per week).

(D) Motor spike amplitude of inhibitory responses evoked in the ipsilesional flexor muscle during the swing phase upon short-pulse photostimulation of glutamatergic CnF or PPN neurons as a function of locomotor score (n = 6 CnF and n = 7 PPN per week).

(E) Motor spike amplitude of inhibitory responses evoked in the ipsilesional extensor muscle during the stance phase upon short-pulse photostimulation of glutamatergic CnF or PPN neurons as a function of locomotor score (n = 6 CnF and n = 7 PPN per week).

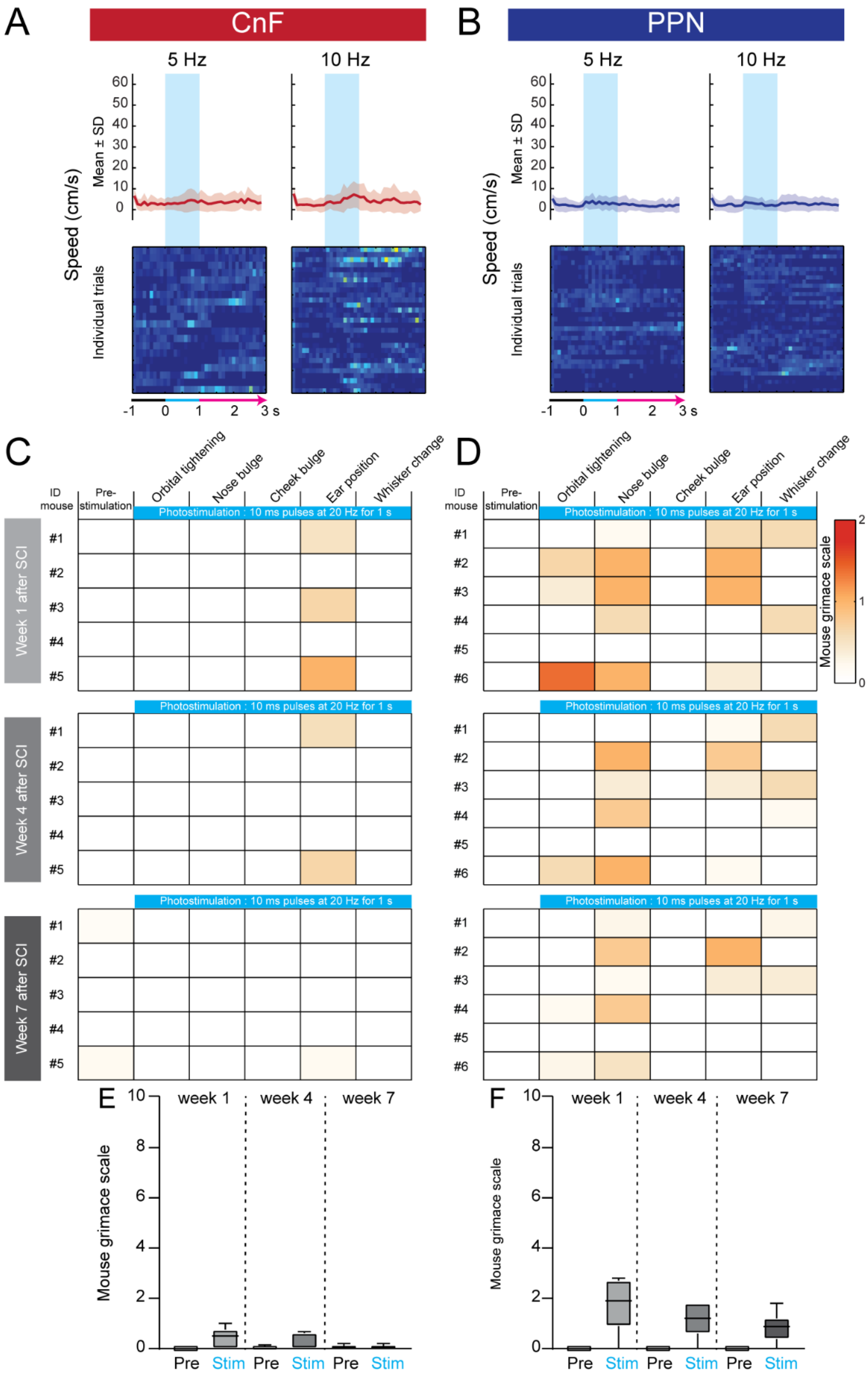
A
CnF
5 Hz
10 Hz
Speed (cm/s)
Mean ± SD
60
50
40
30
20
10
0
Individual trials
-1
0
1
2
3 s
B
PPN
5 Hz
10 Hz
Speed (cm/s)
Mean ± SD
Individual trials
C
ID mouse
Pre-stimulation
Orbital tightening
Nose bulge
Cheek bulge
Ear position
Whisker change
Photostimulation : 10 ms pulses at 20 Hz for 1 s
Week 1 after SCI
Week 4 after SCI
Week 7 after SCI
#1
#2
#3
#4
#5
D
ID mouse
Pre-stimulation
Orbital tightening
Nose bulge
Cheek bulge
Ear position
Whisker change
Photostimulation : 10 ms pulses at 20 Hz for 1 s
#1
#2
#3
#4
#5
#6
Mouse grimace scale
2
1
0
E
Mouse grimace scale
week 1
week 4
week 7
10
8
6
4
2
0
Pre
Stim
F
Mouse grimace scale
week 1
week 4
week 7
Pre
Stim

Fig. S9: No locomotor initiation upon low frequency photostimulation after chronic SCI and no reliable signs of stress or pain upon 20 Hz photostimulation after SCI. Related to Figure 5.

(A-B) Mean and SD of locomotor speed evoked upon 1 s trains of photostimulation of glutamatergic neurons of the CnF (A, n = 5) or PPN (B, n = 7) at 5 and 10 Hz 8 weeks after SCI. Color-coded matrices represent individual trials (A, 5 Hz: 20 trials, 10 Hz: 29 trials; B, 5 Hz: 31 trials, 10 Hz: 35 trials; 100 ms bins). Unlike 20 and 50 Hz, 5 and 10 Hz photostimulation of glutamatergic CnF neurons failed to initiate locomotor bouts.

(C-D) Grimace scale features scored prior and during photostimulation of glutamatergic neurons of the CnF (C) or PPN (D) at 20 Hz at 1, 4, and 7 weeks after SCI at rest.

(E-F) Average mouse grimace scale evoked by stimulation of the CnF (E) and PPN (F) (Pre versus stim / week / group, Wilcoxon matched-pairs signed rank tests, $p > 0.05$).

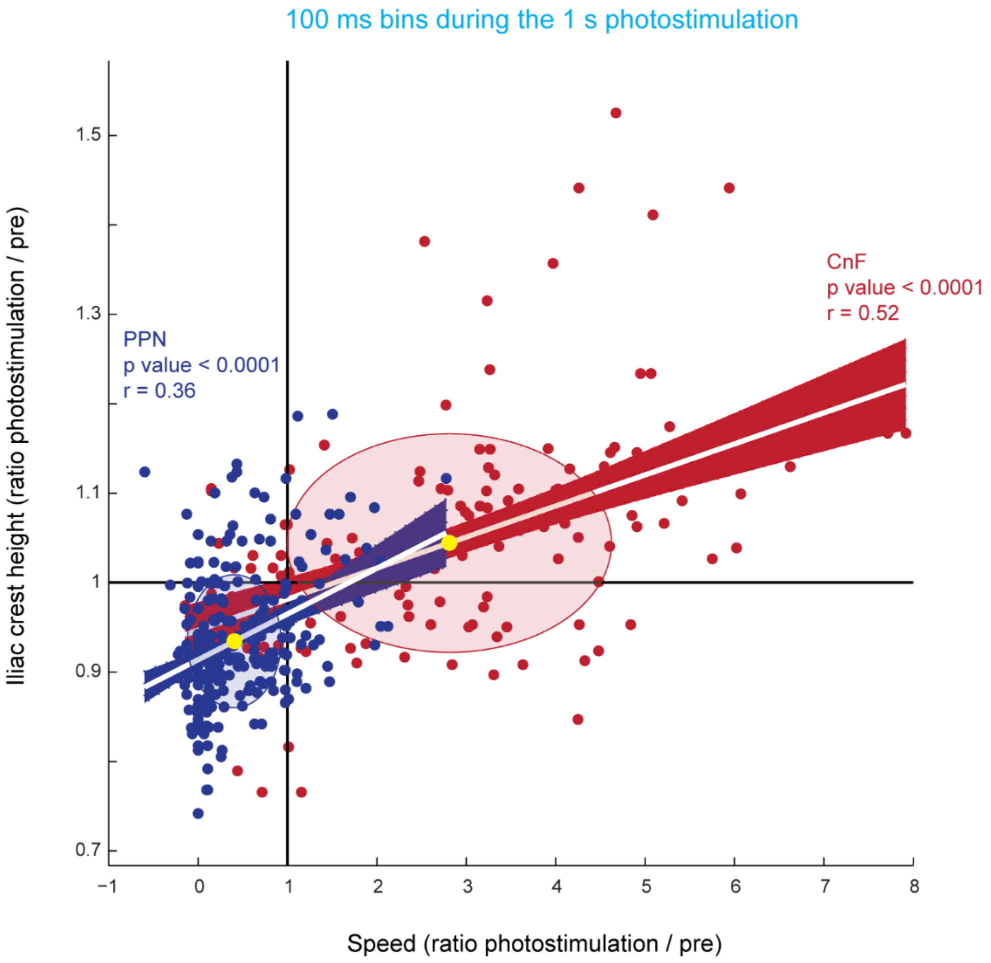

Fig. S10: Speed and posture upon 1 s trains of photostimulation of glutamatergic CnF versus PPN neurons after chronic SCI. Related to Figure 6.

Iliac crest height as a function of speed normalized on pre-stimulation data. 1 s photostimulation period was divided in 100 ms bins (10 bins). Each circle represents a bin, a tenth of trial. Significant linear regressions (white line, 95% confidence interval in red or blue) between both parameters upon photostimulation of the CnF or PPN (n = 150 bins for 4 CnF mice and 280 bins for 7 PPN mice, 100 ms bins). Yellow circles represent the mean of the iliac crest height and speed ratios; ellipses represent interpolation of the SD in the 2-dimensions. Negative ratios for speed represent rare cases of backward locomotion.

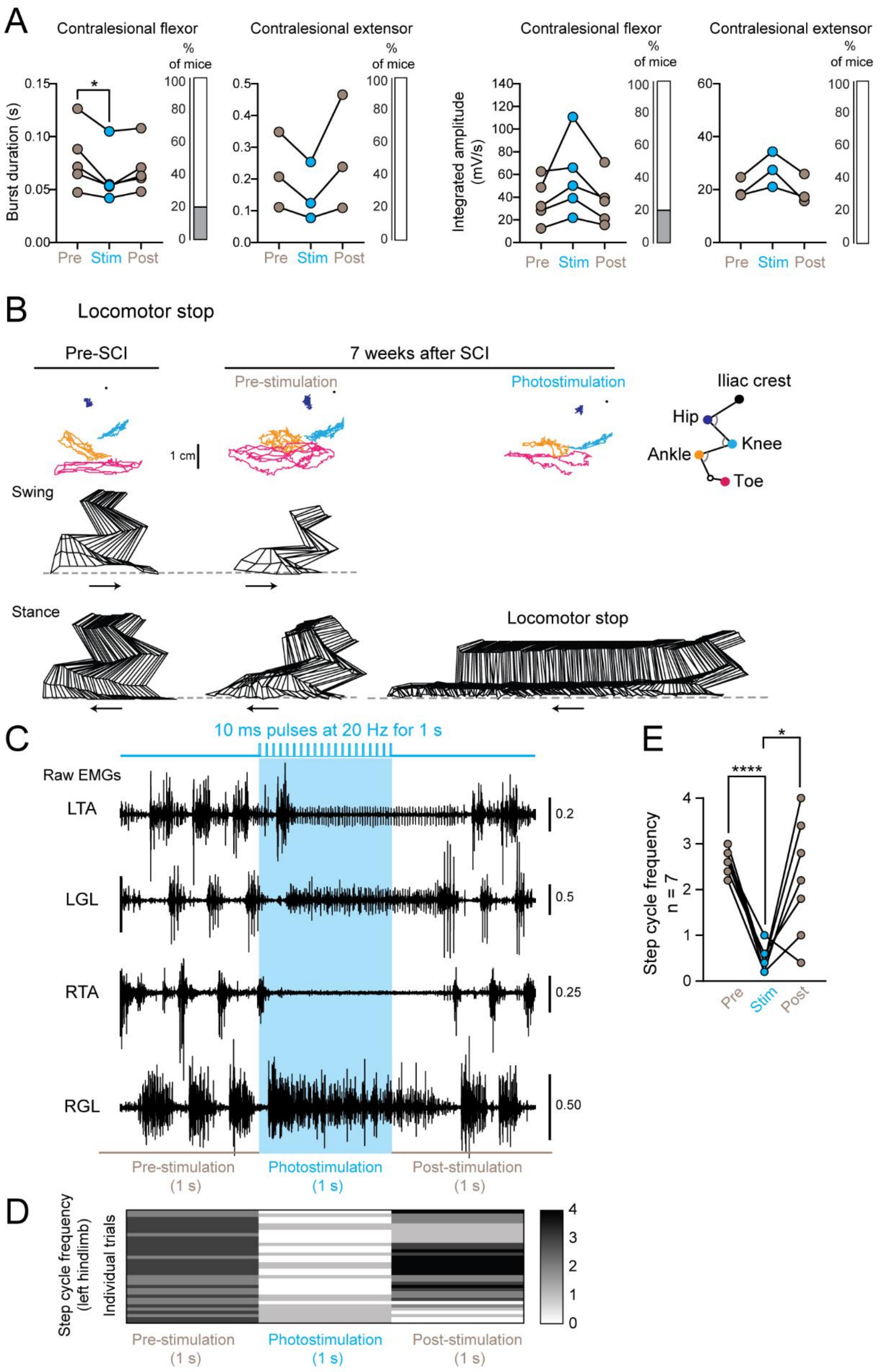
A
Contralesional flexor
Contralesional extensor
% of mice
Burst duration (s)
Integrated amplitude (mV/s)
Pre
Stim
Post
B
Locomotor stop
Pre-SCI
7 weeks after SCI
Pre-stimulation
Photostimulation
Iliac crest
Hip
Knee
Ankle
Toe
1 cm
Swing
Stance
Locomotor stop
C
10 ms pulses at 20 Hz for 1 s
Raw EMGs
LTA
LGL
RTA
RGL
0.2
0.5
0.25
0.50
Pre-stimulation (1 s)
Photostimulation (1 s)
Post-stimulation (1 s)
D
Step cycle frequency (left hindlimb)
Individual trials
E
Step cycle frequency n = 7
****
*

Fig. S11: 1 s trains of photostimulation of glutamatergic CnF or PPN neurons after chronic SCI during treadmill locomotion. Related to Figure 7.

(A) 1 s trains of photostimulation of glutamatergic CnF neurons after chronic SCI. Burst duration and integrated burst amplitude of background EMG activity in contralesional right flexor and extensor muscles (n = 5 for the contralesional flexor and n = 3 for the contralesional extensor, Friedman test [p = 0.009] with Dunn’s multiple comparison post hoc test, *p < 0.05). Percentage of mice showing a significant decrease (i.e., improvement in white) or no change (gray) in the burst duration and percentage of mice showing a significant increase (i.e., improvement in white) or no change (gray) in the integrated amplitude (n = 5 for the contralesional flexor and n = 3 for the contralesional extensor, Mann-Whitney test or unpaired t-test according to the normality of the distribution).

(B-E) 1 s trains of photostimulation of glutamatergic PPN neurons after chronic SCI. (B) Cyclic trajectories of hindlimb joints (anchored on the iliac crest) during locomotion before and after chronic SCI (same PPN mouse). Stick diagrams illustrate the swing and stance phases (arrows show the direction of movement). (C) Raw EMG recordings upon a 1 s train of photostimulation. (D) Step cycle frequency colormap of pre/stim/post periods of 35 trials (7 mice, 5 trials/mouse). (E) Mean step cycle frequency of each mouse (n = 7, repeated measures one-way ANOVA [p = 0.0065] with Tukey's multiple comparisons test, ****p < 0.0001, *p < 0.05).

Abbreviations: see Figure 1 legend.

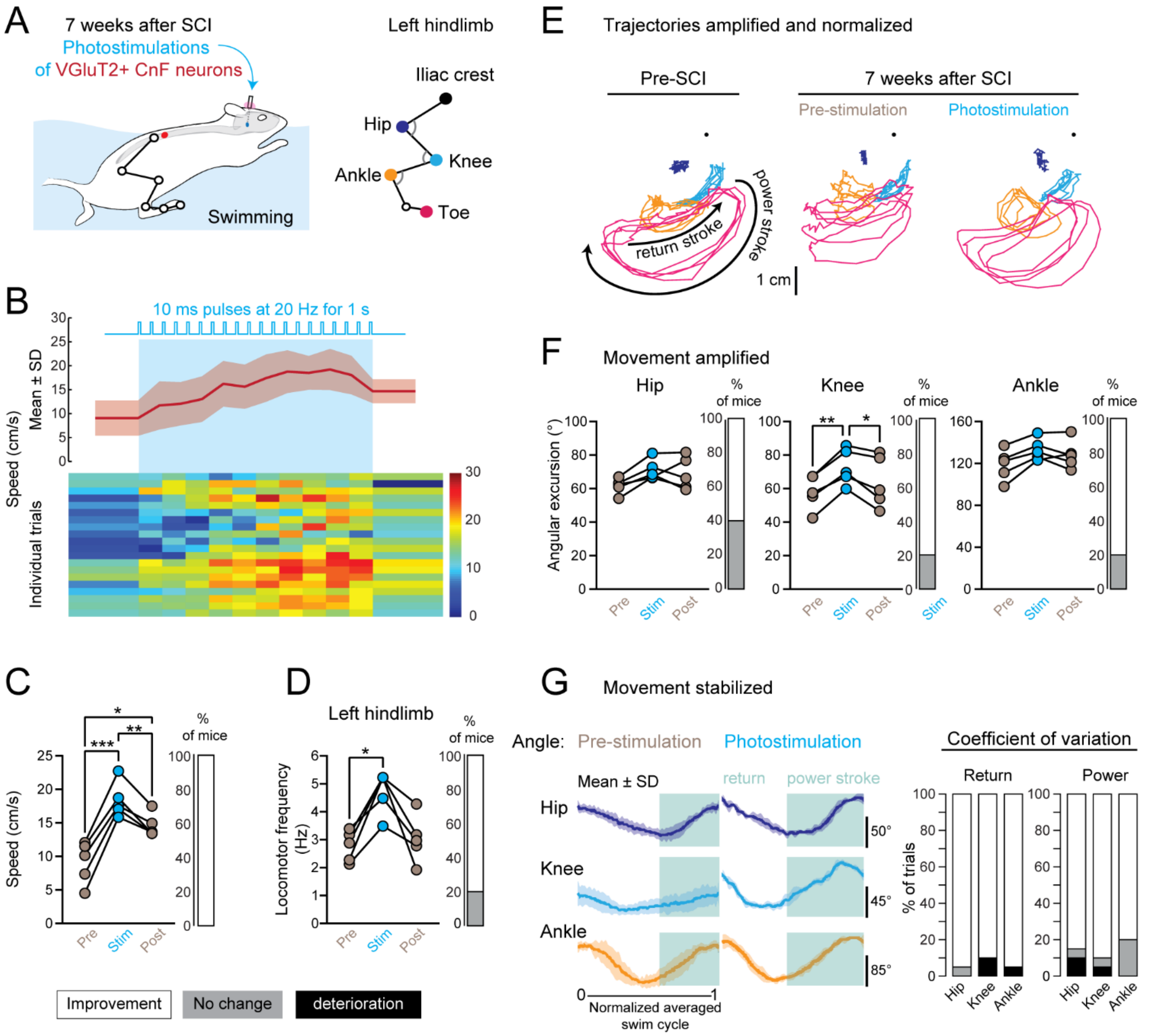

Fig. S12: Functional improvement of swimming upon long trains of photostimulation of glutamatergic CnF neurons after chronic SCI. Related to Figure 7.

(A) Kinematic analysis of the ipsilesional hindlimb evoked upon long trains of photostimulation (10 ms at 20 Hz for 1 s) of glutamatergic CnF neurons during swimming 7 weeks after SCI (n = 5 mice, 4 trials per mouse).

(B) Mean and SD of the swimming and individual trials color-coded as a function of speed (100 ms bins, 4 trials per mouse).

(C) Mean swimming speed (500 ms pre/stim/post periods, last 500 ms of the 1 s stimulation; repeated measures one-way ANOVA [$p = 0.0004$] with Tukey's multiple comparisons test, *$p < 0.05$, **$p < 0.01$, ***$p < 0.001$). 100% of mice exhibited increased speed upon photostimulation (Mann-Whitney test or unpaired t-test according to the normality of the distribution).

(D) Mean swimming frequency upon photostimulation (Friedman test [$p = 0.02$] with Dunn's multiple comparisons test, *$p < 0.05$). Percentage of mice exhibiting increased locomotor frequency upon photostimulation (80%, Mann-Whitney test or unpaired t-test according to the normality of the distribution).

(E) Trajectories of the hip, knee, ankle, and toe anchored to the iliac crest during a swimming bout before and 7 weeks after SCI.

(F) Mean angular excursion of the ipsilesional hip, knee, and ankle (knee: repeated measures one-way ANOVA [$p = 0.004$] with Tukey's multiple comparisons test, *$p < 0.05$, **$p < 0.01$). Percentage of mice exhibiting a significant increase in their angular excursion upon photostimulation (60% for the hip and 80% for the knee and ankle; Mann-Whitney test or unpaired t-test according to the normality of the distribution).

(G) Mean and SD of joint angles during normalized swim cycles upon photostimulation (512 bins). Percentage of trials ($n = 20$) exhibiting a significant decrease in coefficients of variation (i.e., improvement) of joint angles during return and power strokes upon photostimulation (pre bins vs. stim, Mann-Whitney test or unpaired t-test according to the normality of the distribution).

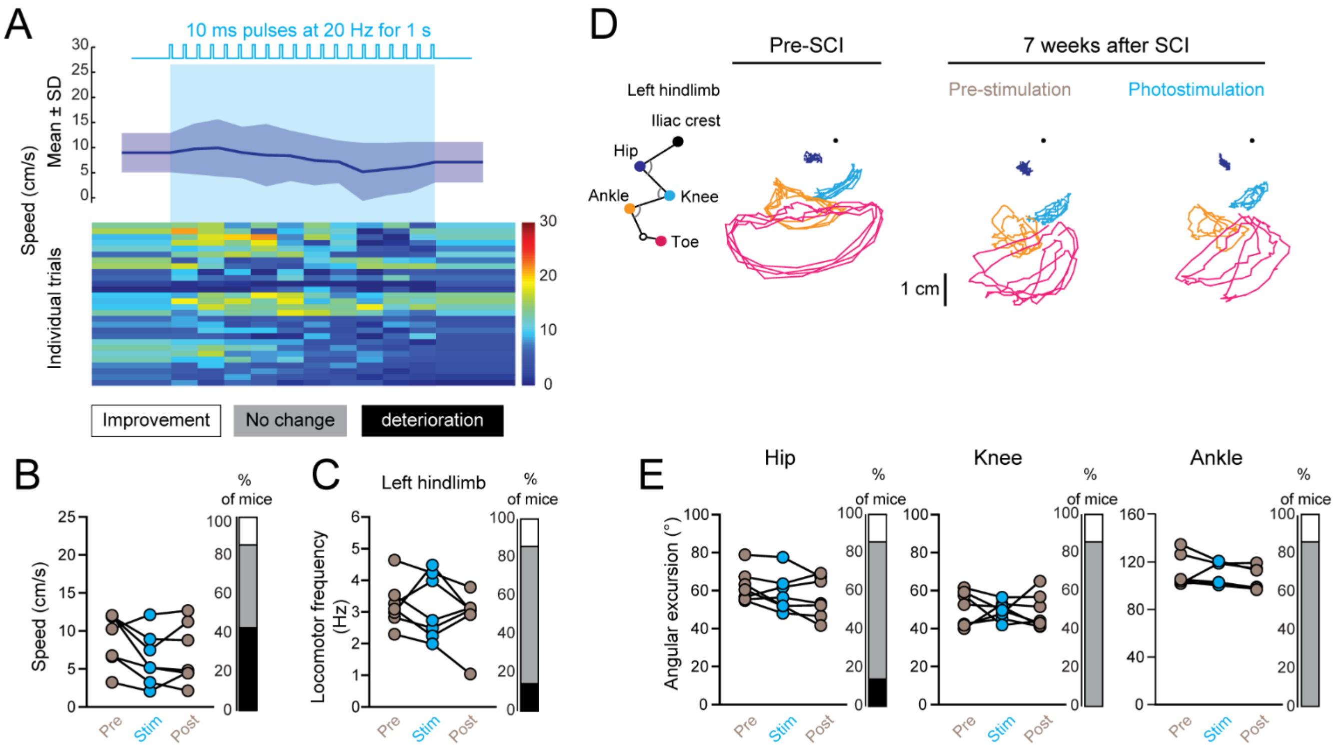

Fig. S13: 1 s trains of photostimulation of glutamatergic PPN neurons failed to improve swimming after chronic SCI. Related to Figure 7.

(A) Mean and SD of the swimming speed and individual trials color-coded as a function of speed (n = 7 mice, 100 ms bins, 4 trials per mouse).

(B) Mean swimming speed (500 ms pre/stim/post periods, last 500 ms of the 1 s stimulation. Percentage of mice exhibiting an increase (i.e., improvement) or decrease (i.e., deterioration) in speed upon photostimulation (Mann-Whitney test or unpaired t-test according to the normality of the distribution).

(C) Mean swimming frequency upon photostimulation. Percentage of mice exhibiting an increase (i.e., improvement) or decrease (i.e., deterioration) in locomotor frequency upon photostimulation (Mann-Whitney test or unpaired t-test according to the normality of the distribution).

(D) Trajectories of the hip, knee, ankle, and toe anchored to the iliac crest during a swimming bout before and 7 weeks after SCI.

(E) Mean angular excursion of the ipsilesional hip, knee, and ankle. Percentage of mice exhibiting a significant increase (i.e., improvement) or decrease (i.e., deterioration), or absence of changes in their angular excursion upon photostimulation (Mann-Whitney test or unpaired t-test according to the normality of the distribution).
